# Neutron Scattering Studies of Heterogeneous Catalysis

**DOI:** 10.1021/acs.chemrev.3c00101

**Published:** 2023-06-14

**Authors:** Xinbin Yu, Yongqiang Cheng, Yuanyuan Li, Felipe Polo-Garzon, Jue Liu, Eugene Mamontov, Meijun Li, David Lennon, Stewart F. Parker, Anibal J. Ramirez-Cuesta, Zili Wu

**Affiliations:** † Chemical Sciences Division, Oak Ridge National Laboratory, Oak Ridge, Tennessee 37381, United States; ‡ Neutron Scattering Division, Oak Ridge National Laboratory, Oak Ridge, Tennessee 37831, United States; § Manufacturing Science Division, Oak Ridge National Laboratory, Oak Ridge, Tennessee 37831, United States; ∥ School of Chemistry, Joseph Black Building, University of Glasgow, Glasgow G12 8QQ, United Kingdom; ⊥ ISIS Pulsed Neutron and Muon Facility, STFC Rutherford Appleton Laboratory, Chilton, Didcot, Oxon OX11 0QX, United Kingdom; # Neutron Technologies Division, Oak Ridge National Laboratory, Oak Ridge, Tennessee 37831, United States; ¶ Center for Nanophase Materials Sciences, Oak Ridge National Laboratory, Oak Ridge, Tennessee 37831, United States

## Abstract

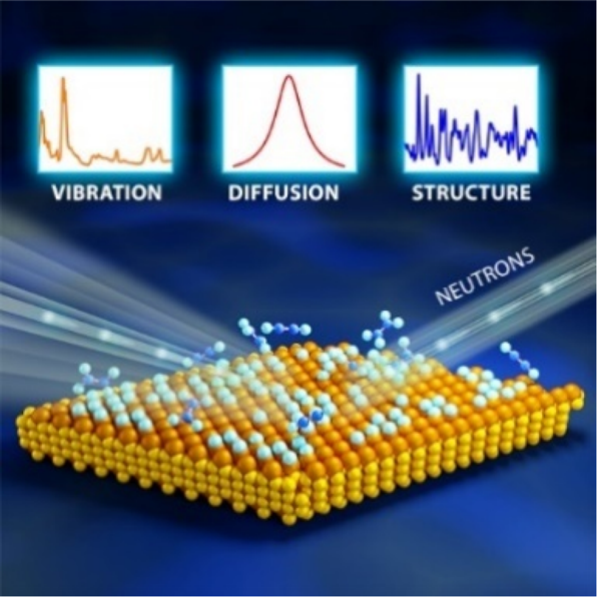

Understanding the structural dynamics/evolution of catalysts and the related surface chemistry is essential for establishing structure–catalysis relationships, where spectroscopic and scattering tools play a crucial role. Among many such tools, neutron scattering, though less-known, has a unique power for investigating catalytic phenomena. Since neutrons interact with the nuclei of matter, the neutron–nucleon interaction provides unique information on light elements (mainly hydrogen), neighboring elements, and isotopes, which are complementary to X-ray and photon-based techniques. Neutron vibrational spectroscopy has been the most utilized neutron scattering approach for heterogeneous catalysis research by providing chemical information on surface/bulk species (mostly H-containing) and reaction chemistry. Neutron diffraction and quasielastic neutron scattering can also supply important information on catalyst structures and dynamics of surface species. Other neutron approaches, such as small angle neutron scattering and neutron imaging, have been much less used but still give distinctive catalytic information. This review provides a comprehensive overview of recent advances in neutron scattering investigations of heterogeneous catalysis, focusing on surface adsorbates, reaction mechanisms, and catalyst structural changes revealed by neutron spectroscopy, diffraction, quasielastic neutron scattering, and other neutron techniques. Perspectives are also provided on the challenges and future opportunities in neutron scattering studies of heterogeneous catalysis.

## 1 Introduction

Achieving the Net Zero carbon goals by 2050 set by many countries requires aggressive decarbonization, which entails both the reduction in, and capture of CO_2_ emissions from the use of fossil fuels.^1^ Increasing the use of renewable energy sources such as wind, solar, hydropower, and biomass will help to reduce CO_2_ emissions but currently makes up only 1/3 of the global power capacity. In the intermediate and foreseeable future, fossil fuels are still the primary energy resources for our society. Reduction of the consumption of fossil fuels can effectively decrease CO_2_ emissions but requires improving the energy and chemical efficiency of the processes so that the societal demand for energy and fuels can still be met. In both aspects, catalysis can play a significant role in decarbonization by improving the process efficiency in terms of activity and selectivity and transforming renewal resources, including biomass and wastes such as plastics and CO_2_, into products that are otherwise derived from fossil fuels.^2^ Realizing the potential of catalysis in decreasing the carbon footprint presents a grand challenge in developing new catalytic materials with unprecedented efficiency. A fundamental understanding of the structure–catalysis relationships in these conventional and new chemical reactions is indispensable for designing such new catalysts and thus addressing the decarbonization challenge.

In the pursuit of understanding the chemical transformations of catalytic reactions and the structural evolution of catalysts at the molecular level, a suite of advanced experimental methods has been developed in the studies of catalysis science, particularly for heterogeneous thermal catalysis.^3−6^ These include the use of photons (diffraction and spectroscopy using X-ray, infrared (IR), Raman, ultraviolet–visible (UV–vis)),^6−12^ electrons (microscopy, surface science methods),^13−15^ and neutrons.^16,17^ While each method provides valuable information on some aspects of catalysis, multiple approaches, often termed multimodal, are desired to provide a complete picture of the working catalysts under reaction conditions.

Among the many characterization approaches, neutron scattering can provide not only complementary catalytic information to other scattering techniques, typically photons and electrons, but also unique insights into catalyst structures and reaction mechanisms of light elements such as hydrogen, nitrogen, and oxygen, or neighboring elements that are difficult or not possible to interrogate by other methods. Neutrons interact with nuclei rather than electrons. Thus, to a neutron, most matter is empty space allowing penetration deep into a catalyst bed or through the thick reactor walls necessary for reactions in extreme conditions. Due to the weak interaction, neutron irradiation does not alter the catalyst, unlike X-ray techniques, where sample damage can be problematic. Additionally, each isotope has its unique neutron scattering cross section. This unusual isotopic sensitivity allows contrast variation and potential discrimination of elements with similar atomic numbers.^18−20^

**Figure 1:**
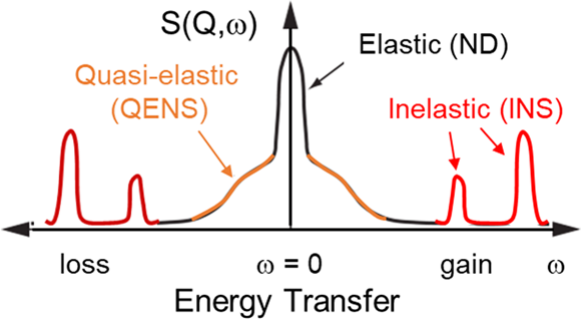
An illustration of typical neutron scattering methods for heterogeneous catalysis investigations. *S* – intensity, *Q* – momentum transfer, ω – energy transfer

For catalysis research, neutron methods can be classified as elastic (diffraction and imaging) and inelastic (spectroscopic) techniques, as depicted in Figure 1, which are used to investigate where atoms are (structure) and what atoms do (function). Neutron diffraction (ND) has significantly contributed to catalysis as a complement to X-ray-based techniques, especially in revealing the location of light elements in the structure of the catalyst under reaction conditions.^20−24^ Through investigation of the broadening of the diffraction peak, quasi-elastic neutron scattering (QENS) provides information on molecular motions (diffusion and rotational) on a range of time scales (picosecond to nanosecond) at Ångstrom length scales,^25^ complementary to the typical dynamic information from nuclear magnetic resonance (NMR) studies.^26,27^ Upon a change in the energy of the scattered neutrons, inelastic neutron scattering (INS), a neutron analog to optical vibrational spectroscopy, provides access to all types of vibrational and translational modes without any selection rules; thus affording chemical spectroscopy information on the catalyst and surface species.^16,28,29^ Advantageous over conventional optical spectroscopy techniques, INS can bestow catalytic mechanistic insights into opaque materials and especially spectroscopic modes in the low energy range (typically below 1000 cm^–1^), which is difficult for infrared (inaccessible) and Raman (dominated by phonon modes) spectroscopies.

There have been some excellent reviews^17,29−36^ and book chapters^16,28,37−40^ on the application of neutron scattering to heterogeneous catalysis. Most of these have focused on a single technique, especially INS, with limited comprehensive reviews^36,37^ on various neutron techniques. With the recent advancement of neutron sources, beamlines, and environmental reactor cell setups, neutron studies of catalysis have seen significant progress. Hence, a timely review of neutron scattering studies of heterogeneous catalysis is warranted. This work will overview the applications, especially in the past decade or so, of different neutron scattering techniques in heterogeneous catalysis with a focus on studies of thermal catalysis. After a brief introduction to the fundamentals of neutron scattering, neutron spectroscopy, diffraction, and other techniques will be reviewed regarding the theory, reactor designs, and catalysis studies. Multimodal approaches combining two or more techniques, either all neutron methods or a mixture with other approaches, will also be briefly reviewed in catalysis research. The review will conclude with a summary and perspective on neutron scattering studies of heterogeneous catalysis.

## 2 A Brief Introduction to Neutron Scattering

Smaller (subatomic) particles are often used as probes to measure the atomic level structure and dynamics. Commonly used techniques include X-ray scattering, Raman/infrared spectroscopy, and transmission electron microscopy. Compared to the above methods using photons or electrons, neutron scattering (i.e., using neutrons as the probe particles) may sound less familiar to many readers. The neutron is an elementary particle with zero electric charge, a rest mass close to that of the proton, and a magnetic moment.^41^ When a neutron strikes an atom, it interacts with the nucleus and can be either absorbed or scattered. Absorption, which leads to nuclear transformation and is the basis of all neutron detectors, is not of interest in the applications to be discussed here. Instead, we learn the structure and dynamics of the material from the scattered neutrons.^42^ The scattering can be elastic or inelastic because a neutron has mass; all scattering results in a change of direction, and hence momentum, of the neutron. There is no energy exchange for elastic scattering, and the neutrons change direction upon collision. In the case of inelastic scattering, the neutron exchanges energy and momentum with the scattering nucleus (the scatterer). The scattered neutron may gain or lose energy (Figure 1). The energy transferred from the neutron appears as the scatterer’s rotational, vibrational, or translational energy.^28^ The scattering can also be coherent or incoherent. Incoherent scattering measures the correlation between the positions of an atom at time zero and at a later time, with each atom contributing independently to the total scattering by simple summation. In contrast, coherent scattering describes interference between waves from the scattering of a single neutron from all nuclei—examples are the Bragg peaks seen in diffraction or the phonon dispersion seen in an inelastic scattering. In 1994, the Nobel Prize in Physics was awarded “for pioneering contributions to the development of neutron scattering techniques for studies of condensed matter” jointly to Bertram N. Brockhouse “for the development of neutron spectroscopy” and to Clifford G. Shull “for the development of the neutron diffraction technique”.^43^

Neutron scattering shares many similarities with X-ray scattering, but there are also significant differences. Neutrons interact with matter via the relatively weak neutron–nucleon interaction at a very short-range (∼fm). X-rays interact with matter via electromagnetic interaction, which scales with electron density. Because the atomic nucleus is only ∼1/1000th of the diameter of an atom, to a neutron, most matter is empty space. Consequently, a principal advantage of neutrons over X-rays is that neutrons are highly penetrating. This capability of neutrons ensures that the results obtained from neutron scattering are representative of the bulk. For neutron scattering, the scattering cross section is both element- and isotope-dependent, while for X-ray scattering, it is a monotonic function of atomic number.

**Figure 2:**
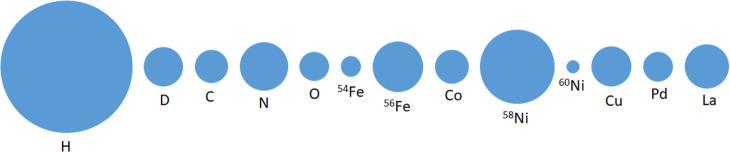
Neutron scattering cross sections of selected elements (isotopes)^44^ that are relevant to catalysis research.

Moreover, neutron scattering cross sections for light elements are relatively large compared to the much heavier metals. These features are illustrated in Figure 2, which indicates that 1) neutron scattering is ideal for studying catalytic systems involving light elements, mainly hydrogen, which could not be directly detected by X-rays; 2) it is possible for neutron scattering to distinguish adjacent elements in the periodic table since they may have different neutron cross sections; 3) one can use isotope labeling in a neutron scattering experiment to resolve structural or dynamical information that is otherwise inaccessible; 4) more complicated sample environment equipment can be used in a neutron scattering experiment, such as high-pressure and high-temperature sample cells made of metals, without blocking the incident/scattered beam.^28,40^ There are also disadvantages. Neutron sources have much lower flux than X-rays; thus, much longer data acquisition time and larger sample quantities are required. Neutrons are expensive and difficult to produce. Consequently, while X-ray-based facilities can be easily accessed, there are relatively few neutron facilities worldwide where neutron scattering experiments can be carried out.

Neutrons can be produced by fission or spallation.^45^ The fission approach requires a nuclear reactor, where controlled chain reactions happen in nuclear fuels (containing ^235^U) to release neutrons. The neutron beams produced this way are continuous (i.e., steady state) with high average flux. The spallation approach is entirely different. It uses a particle accelerator to generate a high-energy proton beam directed onto a “target” made of heavy metals such as Ta, W, or Hg. The interaction of a high-energy proton with the nucleus results in highly excited nuclear states. One of the decay mechanisms is the “evaporation” of neutrons. A significant distinction of this approach is that the beam can be easily pulsed, meaning that the protons, thus the produced neutrons, can be tuned to come in/out as pulses/packets at specific frequencies.

**Figure 3:**
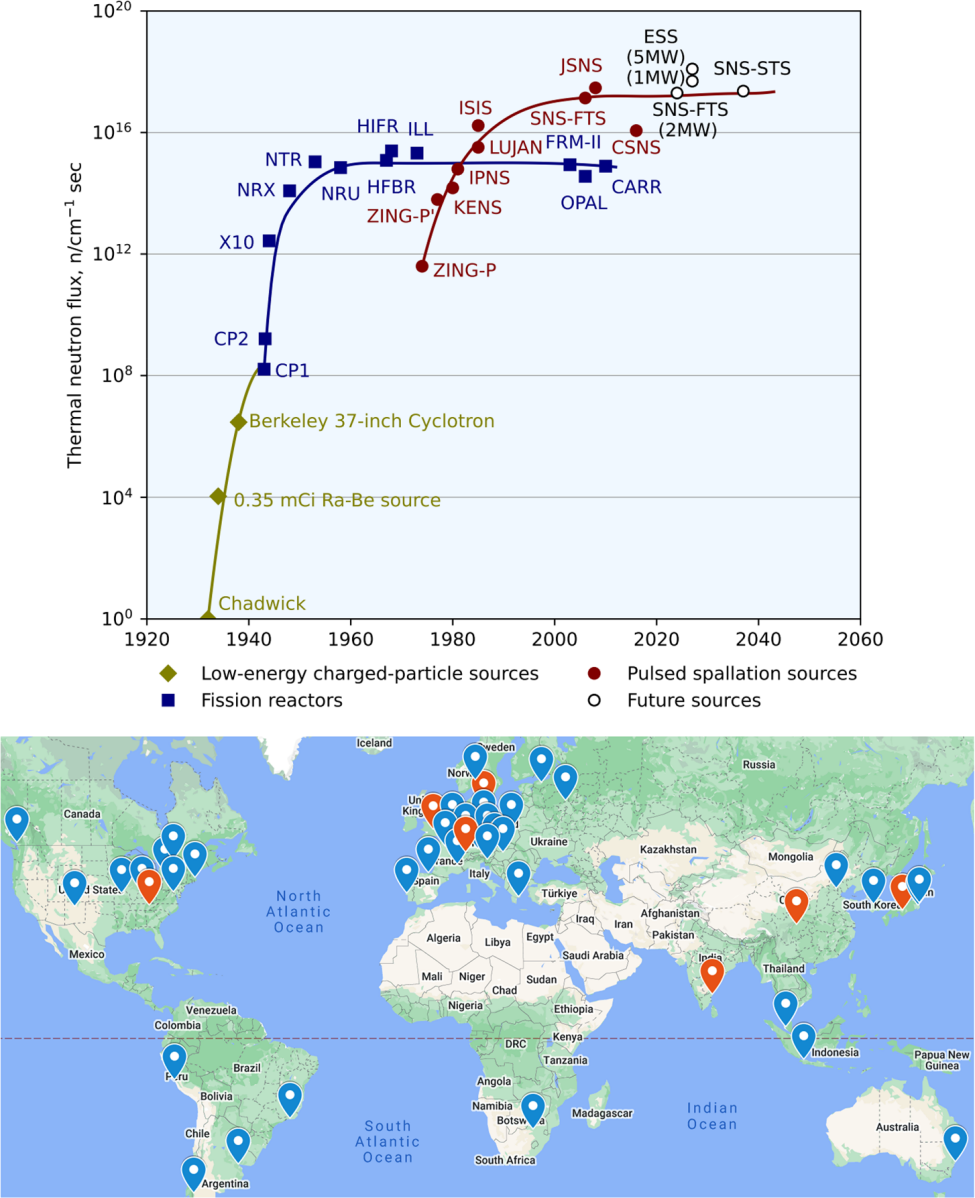
(Top) Evolution of thermal-neutron sources (reactor-based and spallation). Adapted with permission from ref (46). Copyright 2006 AIP Publishing. (Bottom) The location of neutrons sources around the world. Those marked in red are spallation sources, and the rest are reactors. Detailed information about the neutron sources can be found in Table S1 in the Supporting Information.

Most spallation sources are operated in a pulsed mode, which enables the energy or wavelength of the neutrons to be measured by time-of-flight (ToF).^45^ Specifically, since the emission of the neutrons is pulsed and time-stamped, one can measure the time needed for a neutron to travel from the source to the detector and then convert that to velocity and thus energy or wavelength. Modern spallation sources have high peak flux, brightness, and efficiency. A history and locations of neutron sources are presented in Figure 3,^46^ which also highlights some of the currently running spallation sources including ISIS^47^ in the UK, J-PARC^48^ in Japan, and SNS^49^ in the US. There are also newer spallation sources shown in Figure 3, including the China Spallation Neutron Source (CSNS)^50^ that has recently been commissioned, as well as the European Spallation Source (ESS)^51^ and the Second Target Station (STS)^52^ of SNS that are presently being built/designed.

In a neutron scattering experiment (assuming the incident neutron flux is fixed), the scattering intensity (the number of scattered neutrons) depends on three variables: the cross section of the scattering atom (the scatterer), the number of scattering atoms in the neutron beam, and the atomic positions and displacements due to vibrations. The scattering intensity is expressed by the scattering law and can, in principle, be calculated rigorously, assuming all the above information is known.^40,42^ Since the atomic structure and dynamics, which are often the goal of a neutron scattering experiment, can be difficult to extract directly from the experimental data, an atomistic model (or a series of models) is often used to simulate the neutron scattering data, compare with experiment, and then help to interpret the data and obtain insight. For example, when analyzing the complicated diffraction pattern measured on a disordered or nanomaterial, the reverse Monte Carlo method^53,54^ is often used to search for a structural model that can reproduce the total scattering intensities. When assigning the peaks observed in an inelastic neutron scattering experiment, lattice dynamics or molecular dynamics based on density functional theory^55−57^ is a widely used tool to find the structural or dynamical origin (e.g. physical adsorption, chemical reaction, or phase instability) behind the experimental observations.^58−62^ In addition to the commonly used software packages for atomic simulations, tools have been developed to bridge neutron scattering experiments and atomistic models.^54,58,59,61−64^ Nowadays, advanced computing and simulation play an increasingly important role in analyzing and interpreting neutron scattering data.

## 3 Neutron Spectroscopy of Catalysis

Neutron vibrational spectroscopy (NVS), i.e., INS, has dominated the use of neutron techniques for heterogeneous catalysis research thanks to its distinct sensitivity to hydrogen and its involvement in many catalytic reactions. This section will start with an introduction of neutron spectroscopy fundamentals, followed by an overview of applications of NVS for studying different hydrogen and hydrogen-containing species from adsorption and reactions, and a few case studies of other light elements such as oxygen- and nitrogen-containing species in catalysis. For the convenience of the readers, representative examples of the catalysts, adsorbates, and reaction species studied by INS are briefly summarized in Table S2.

### 3.1 Introduction to Neutron Spectroscopy

**Figure 4:**
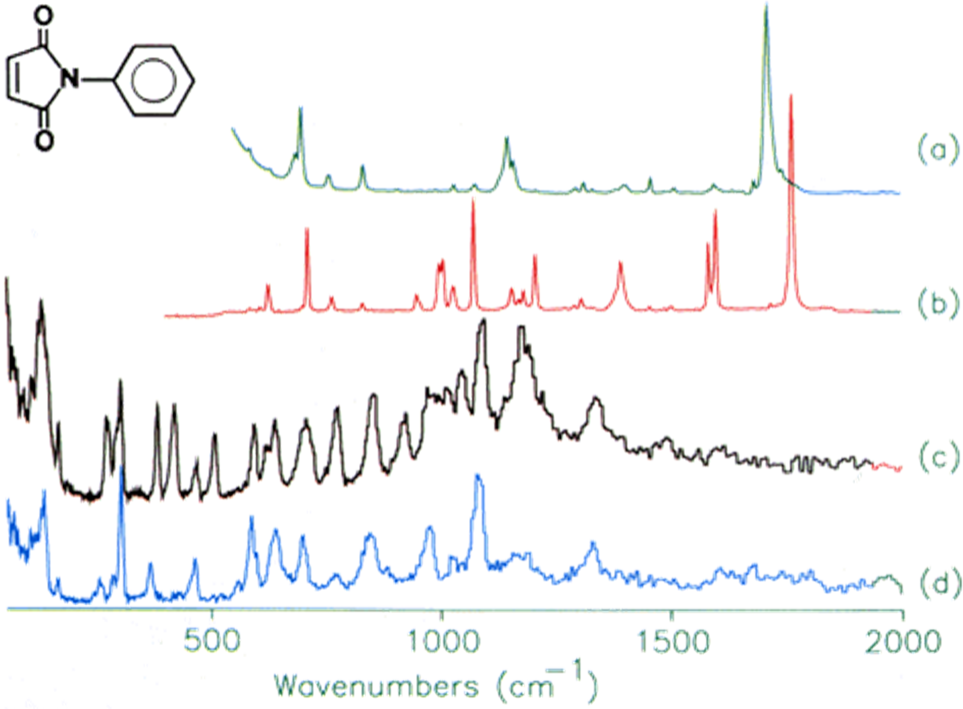
A comparison of the (a) infrared, (b) Raman, and (c) INS spectra measured on the same material (*N*-phenylmaleimide, as shown in the inset). (d) INS spectrum of *N*-(perdeuterophenyl) maleimide. Reproduced with permission from ref (65). Copyright 2006 Elsevier.

INS can be considered the neutron analog of Raman and infrared spectroscopy. Instead of using photons as the probing beam, a neutron beam is directed onto the sample. When INS occurs, one can determine molecular excitations (corresponding to vibrational modes) from the energy loss/gain spectra. INS and Raman/infrared spectroscopy are highly complementary, as summarized in Table 1 and illustrated in Figure 4.

**Table 1:** Unique Capabilities of INS That Make It an Ideal Complementary Technique to Conventional Raman and Infrared Spectroscopy^a^

NVS (INS)	Raman/Infrared spectroscopy
Measures dynamics of nuclei (direct)	Measures response of electrons (indirect)
Not restricted by selection rules, can detect Raman/infrared-inactive modes	Selection rules apply
Great sensitivity to H	Cannot always detect H
High penetration (bulk probe)	Lower penetration degree (surface + bulk)
Easy access to low energy range (librational and translational modes)	Low energy cutoff usually applies (especially for infrared spectroscopy)
*Q* trajectories in the (*Q*, ω)* map; averaging over the Brillouin zone	Γ point (Brillouin zone center) only
Intensity weighted by neutron scattering cross section	Intensity weighted by change in polarizability or dipole moment
Easy to simulate/calculate spectra	More effort to simulate/calculate spectra
Weak interaction, no energy deposition in sample	Potential sample damage via heating, photochemistry etc. under laser irradiation (Raman)
Low sensitivity: 10–100 mg of hydrogenous sample, >1 g of non-hydrogenous material, 3–50 g of catalyst	Highly sensitive: 1–10 mg of sample, 10–1000 mg of catalyst

a *Q*: momentum transfer; ℏω: the energy transfer; ω: angular frequency.

#### 3.1.1 Instrumentation

**Figure 5:**
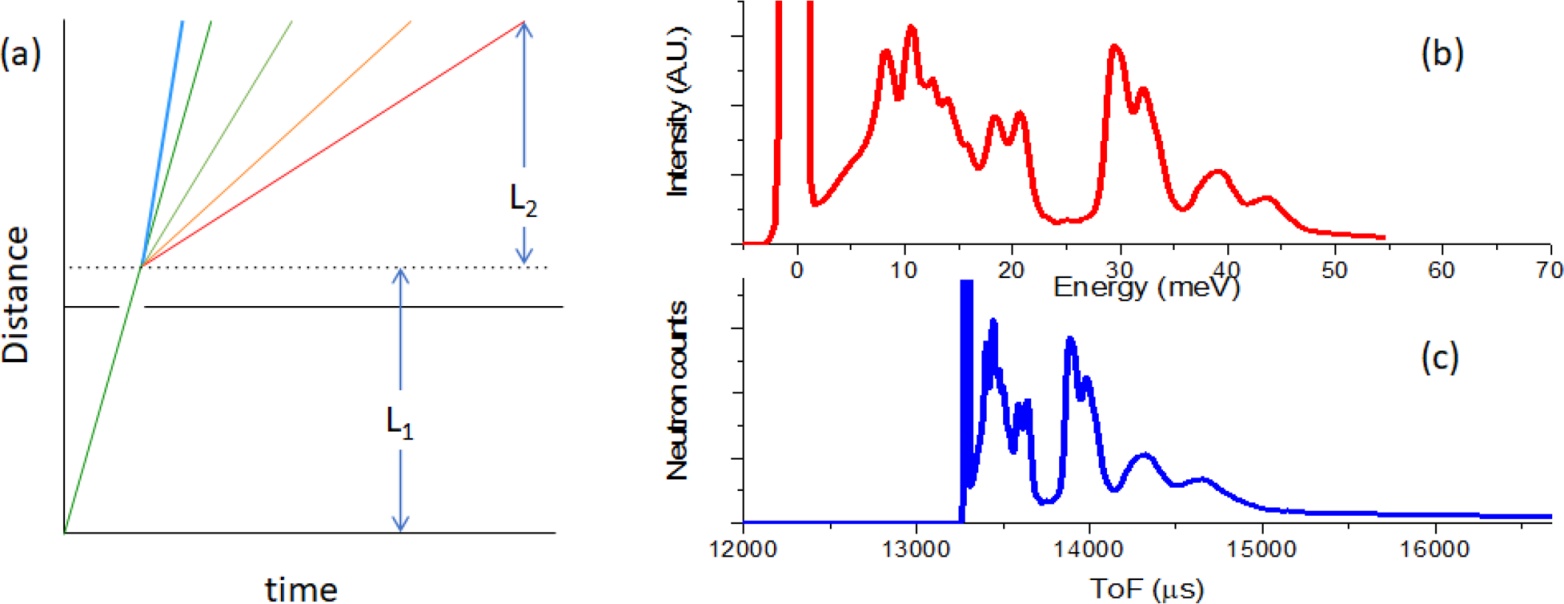
Working mechanism of a DGS. (a) Distance–time plot of the neutrons. L_1_ is the length of the primary flight path from the moderator to the sample, and L_2_ is the length of the secondary flight path from the sample to the detector. (b) the measured INS spectrum derived from (c) after conversion of ToF to energy transfer. (c) the raw ToF spectrum.

For INS spectroscopy, the quantity of interest is generally the energy transfer, i.e., the difference in energy between the incident and scattered neutron. At a pulsed neutron source, the neutron time-of-flight (ToF) is recorded and then converted to energy. There are two modes of operation of a ToF neutron spectrometer: direct geometry and indirect (or inverted) geometry.^45^ In a direct geometry spectrometer (DGS), the incident neutron beam is chopped by a Fermi chopper to select the initial speed/energy. The sample then scatters the monochromatic beam in different directions with different energy loss/gain. An array of detectors is then used to capture the scattered neutrons and record their positions (scattering angle) and total ToF from the neutron source. Since the incident energy is fixed and known, the initial ToF from source to sample can be calculated. Thus, the ToF on the secondary path (between sample and detector) can be derived, from which the final energy can be calculated. The neutrons are histogrammed by their energy transfer, adequately normalized, and the INS spectrum is obtained. This mechanism is illustrated in Figure 5. ToF methods can also be used at continuous sources such as reactors or the Swiss Spallation Neutron Source (SINQ).^66^ The MAPS, MERLIN, and MARI instruments at ISIS, ARCS, and SEQUOIA at SNS are examples of DGSs at pulsed sources. Panther (and its predecessor IN4) at the ILL and DCS at NIST are examples of DGSs at continuous sources.^67,68^ Both of these instruments require complex chopper systems to convert a steady state beam into a pulsed beam. They are also both on thermal sources, so have a maximum energy transfer of ∼1000 cm^–1^.

**Figure 6:**
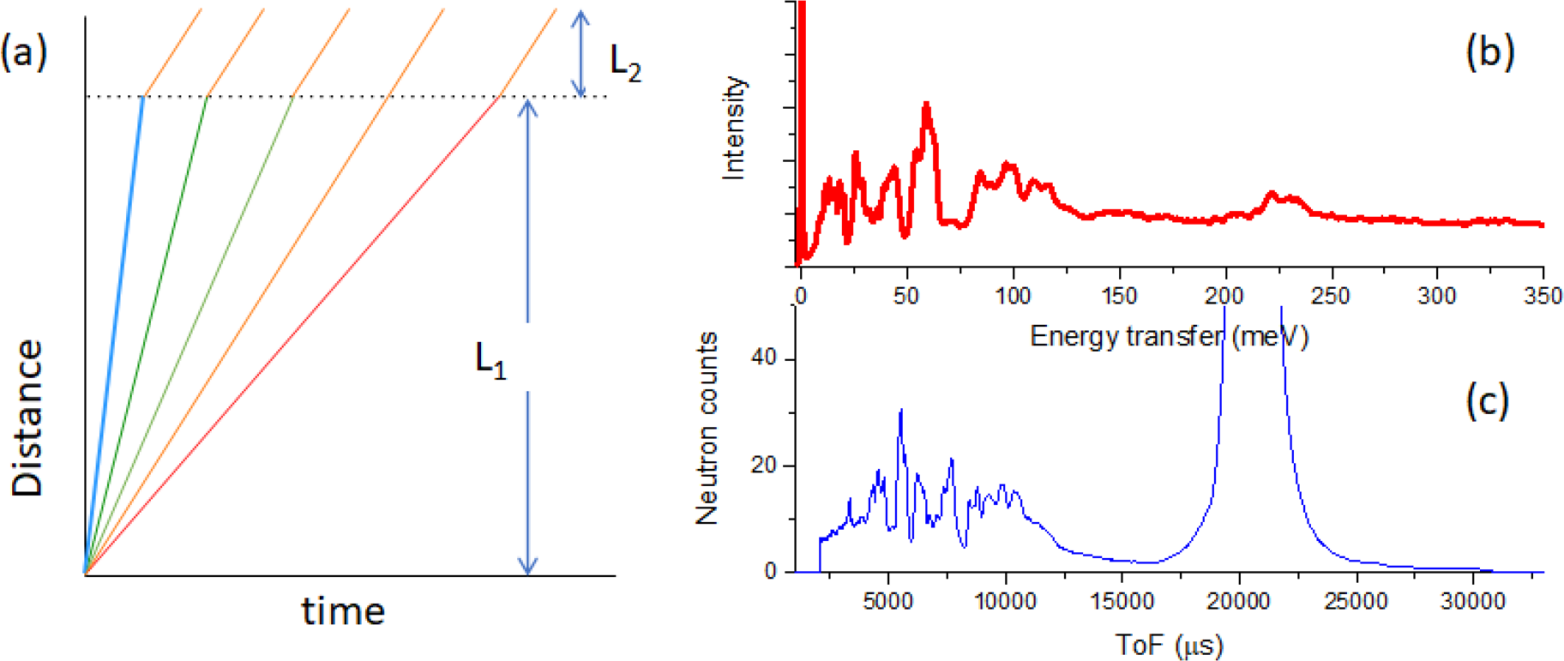
Working mechanism of an IGS. (a) Distance–time plot of the neutrons. L_1_ is the length of the primary flight path from the moderator to the sample, and L_2_ is the length of the secondary flight path from the sample to the detector. (b) The measured INS spectrum derived from (c) after conversion of ToF to energy transfer. (c) the raw ToF spectrum.

An indirect geometry spectrometer (IGS) is the opposite. The incident beam is a white neutron beam consisting of neutrons of a wide range of speed/energy, typically ∼0–1500 meV or higher. A crystal analyzer reflects the scattered neutron beam at a particular reflection angle, by which Bragg’s law selects neutrons with specific wavelengths (thus speed/energy). The reflected beam is then passed through a filter that scatters and absorbs the higher-order reflections, leaving only neutrons with a fixed final energy to reach the detector. This mechanism is illustrated in Figure 6.

At a steady state source, an IGS is somewhat different to that at a pulsed facility. In this case, the instruments are simplified versions of the first type of inelastic spectrometer: the triple axis spectrometer (TAS) invented by Bertram Brockhouse in 1952.^43,69^ A TAS consists of three elements: (i) a monochromator for the incident beam, (ii) the sample, and (iii) a monochromator in the scattered beam. This results in an instrument that, in principle, can access any point in (*Q*,ω) space. However, because it is a point-by-point method it is very slow and the use of two monochromators means that the detected flux is very low. It was realized very early on that replacing the second (analyzing) monochromator by a beryllium filter would greatly increase the detected flux. This type of instrument was installed at NIST (BT4) and the ILL (IN1-BeF) in the late 1970s and for many years were the workhorses of INS of catalysts (see Section 3.2.6). Both instruments have been upgraded: BT4 to FANS and IN1-BeF to Lagrange.^70,71^ Lagrange is unusual for a spectrometer at a reactor, in that it views a hot source (a graphite block heated to ∼2000 °C) so one can access the full 0–4000 cm^–1^ range. Most reactor instruments are limited to ∼1000 cm^–1^ because they view a thermal (i.e., room temperature) source. Lagrange is also unusual in that it uses a combination of a Be filter and a graphite analyzer to improve the resolution over that obtainable with just a Be filter as the analyzer. Presently, the most frequently used IGSs in catalysis research are TOSCA at ISIS, VISION at SNS, and Lagrange at the ILL.

**Figure 7:**
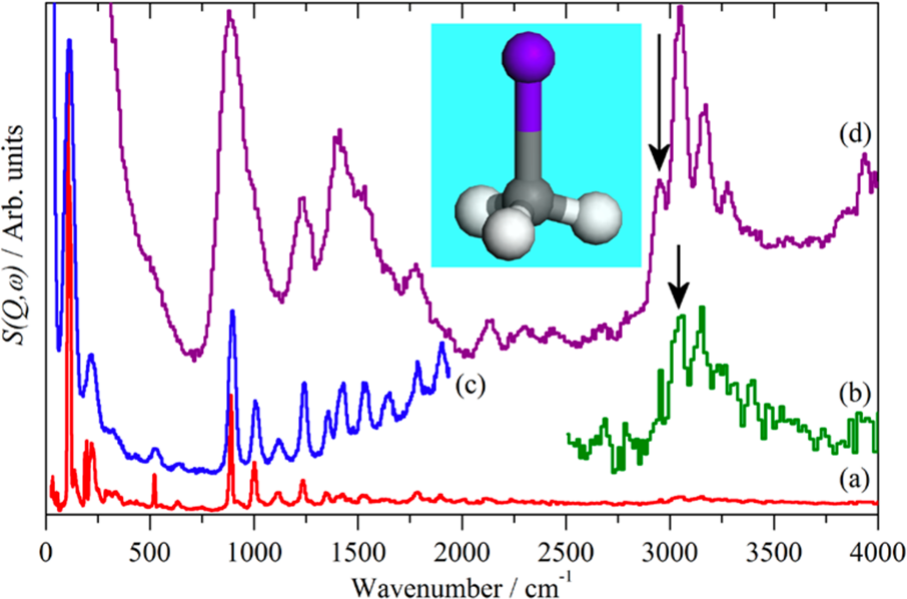
INS spectra of iodomethane recorded at 20 K on (a) TOSCA, (b) TOSCA × 10 ordinate expansion of the 2500–4000 cm^–1^ region, (c) and (d) MAPS with incident energies of 4840 and 2017 cm^–1^, respectively. Reproduced from ref (72). Open access.

DGS and IGS both have their advantages and disadvantages. Typically, a DGS offers more flexibility to cover selected regions in the *S*(*Q,E*) map, but a wider range coverage sacrifices resolution at low energy transfer. An IGS, however, can cover a wide energy range in a single scan with excellent resolution at low energy transfer. The spectra of the same sample collected at DGS and IGS are compared in Figure 7. With its wide dynamic range and good resolution in the important energy range, IGS is suitable as a generic instrument for chemical spectroscopy. If there is a specific feature of interest, the DGS can focus on that feature. Also, because DGS provides the possibility of accessing the high *E* and low *Q* area when a suppressed Debye–Waller factor is needed (such as at elevated temperature or the C–H/N–H/O–H stretch region is of interest), DGS is a better choice and thus might be more suitable for *in situ* catalysis studies.

#### 3.1.2 Sample Environment

**Figure 8:**
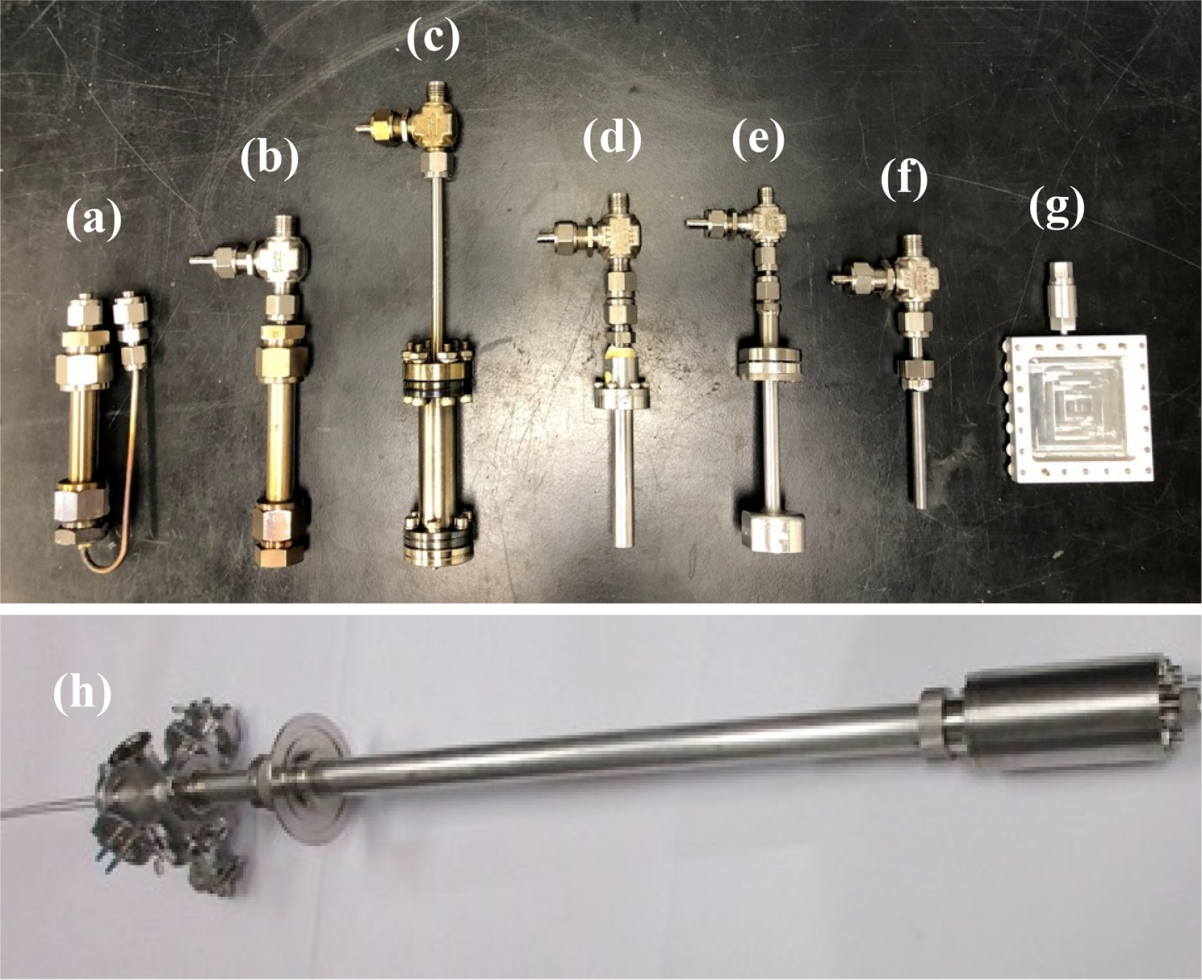
Various reactors used for catalysis neutron scattering experiments. (a, b) Stainless steel Swagelok reactors for flow-through and cyclic gas loading, respectively. (c) A ConflatTM stainless steel can. (d) An aluminum cylindrical can with a diameter of half inch. (e) An aluminum pressure cell that can hold pressure up to 100 bar. (f) is a vanadium cylindrical can, and (g) is an aluminum flat can. Bottom panel (h) shows a sample stick-reactor system developed at SNS for *in situ* catalytic reactions.

Apart from the choice of the spectrometer type, the sample environment (reactor cells) is also a critical factor for heterogeneous catalysis studies where the gas atmosphere, temperature, and pressure need to be controlled. Taking advantage of the high penetration power of neutrons, reactors can be made from various materials ranging from quartz to more durable variants such as steel or copper–beryllium. For example, stainless steel cells are usually used for reactions requiring high temperature and/or high pressure, where there is no need for optically transparent windows (often required in optical spectroscopy and with X-rays). Aluminum produces a lower background and is thus preferred when the reaction conditions are moderate. It is also possible to use quartz cells when it is important to be able to see the sample during the experiment (e.g., to monitor the color change). The reactor can be designed with a separate inlet and outlet for flow-through reactions or a single inlet/outlet for cyclic gas loading. When the sample is highly neutron absorbing or scattering, it is beneficial to use a flat sample holder rather than a cylindrical one. Depending on the maximum reaction temperature, the sample holders can be sealed with indium, lead, or gold wires, as well as aluminum foil or copper gaskets. Some of the reactors used at ISIS and SNS are shown in Figure 8.

In the case of IGS, the spectral measurements typically require a very low temperature (<50 K) to suppress the Debye–Waller factor. This makes it almost impossible to carry out *operando* INS study of heterogeneous catalysis, as most reactions typically require above-ambient temperatures. Therefore, most catalysis studies in INS have adopted a “react and quench” approach, where the sample reactor/stick is taken out of the instrument to perform the high-temperature adsorption and reactions and then returned to the spectrometer for spectral acquisition at low temperatures without exposure to ambient atmosphere. This limitation is less of a problem for diffractometers and DGS. One of the few examples of measuring neutron spectra at reaction temperatures (*in situ* spectroscopy) is the room temperature CO oxidation over a model Pd catalyst investigated on the MAPS spectrometer,^73^ thanks to the spectrometer’s high sensitivity and ability to access low momentum transfer.

#### 3.1.3 INS Spectral Interpretation Aided with Computational Modeling

In an INS spectrum, several features are of interest to the catalytic systems studied: scattering intensity, energy range, peak positions, peak intensity, and peak shape. The scattering intensity is proportional to the number of scattered neutrons versus the energy transferred from the neutrons to the scatterer. The INS spectrum can provide information in the low-energy region. For IGS, the lower limit is usually 10–20 cm^–1^; for DGS, it depends on the incident energy, and it can be as low as ∼1 cm^–1^. Even lower energy can be accessed with quasielastic neutron scattering. However, particularly for IGS, features in INS tend to become weak and poorly resolved above 1600 cm^–1^ due to multiple factors, including the neutron flux, Debye–Waller factor, and recoil. The peak positions of INS bands are signatures of a molecule’s structure and intramolecular forces that determine the atomic displacements; thus, different transitions show different intensities. In theory, the vibrational modes will be observed at the same energies in the INS, infrared, and Raman spectra (see the example shown in Figure 4). Thus, one could obtain complementary information through different types of vibrational spectroscopy. So, it is the best practice that infrared and Raman studies are carried out to obtain complementary data before the INS experiment. The INS peak intensity, the integrated area under the peak, is proportional to the amplitude of motion during the vibration. Since INS is not limited by selection rules, the shape of the INS peak shows additional structural and dynamic information. The integrated spectral intensity is also directly proportional to the total neutron scattering cross section in the beam (in the case of hydrogenous material, it roughly scales with the amount of hydrogen).^28^

Theoretically modeling is often required to analyze and understand the obtained INS spectrum. Neutron scattering shows the cumulative effect on the energies and numbers of the scattered neutrons of all collisions with the catalyst and any adsorbed species. The challenge is to separate the spectrum into the different scattering species using modeling. Using computer simulations, phonon information can be obtained from either quantum or classical calculations such as density functional theory (DFT) and molecular dynamics (MD). In these calculations, experimental information such as the instrument geometry, resolution, and the nature of the sample is considered. The OCLIMAX program^58^ developed at the VISION beamline allows one to calculate the full INS spectrum, including coherent effects and temperature effects for various INS instruments and arbitrary trajectories in the momentum and energy transfer (*Q*-ω) space. The models used to fit the spectrum can provide information on the vibrational modes and dynamics, and indicators of the structure of the catalysts.

### 3.2 INS Studies of Hydrogen-Containing Species

#### 3.2.1 INS from Dihydrogen

**Figure 9:**
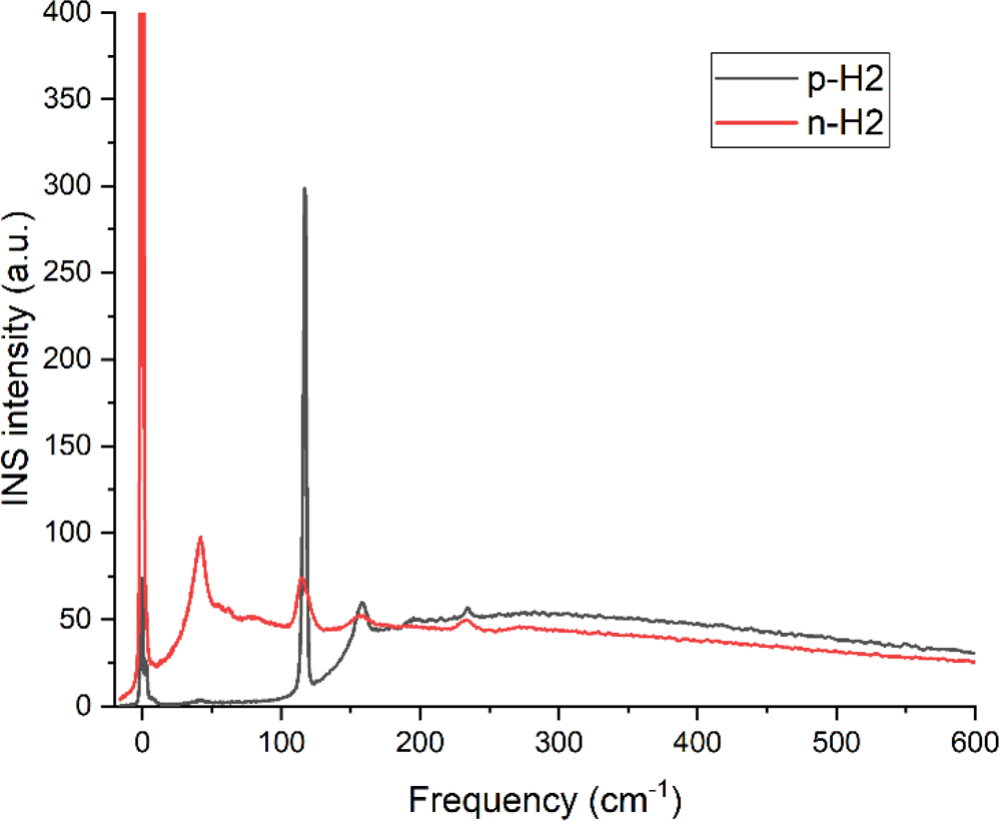
INS spectra of solid *p*-H_2_ (black) and *n*-H_2_ (red) measured at 5 K on VISION. The small elastic intensity for *p*-H_2_ is due to the aluminum sample holder.

A free dihydrogen (H_2_) molecule can be considered as a rigid quantum rotor, with its rotational energy levels expressed as *E* = *BJ*(*J* + 1), where *J* is the rotational quantum number and *B* is the rotational constant, which is 7.35 meV (59.3 cm^–1^, 1 meV = 8.066 cm^–1^) for H_2_ with an H–H distance of 0.74 Å. H_2_ has two spin isomers, *para*-hydrogen (*p*-H_2_, a singlet with spins of the two protons antiparallel to each other) and *ortho*-hydrogen (*o*-H_2_, a triplet with spins of the two protons parallel to each other). Due to symmetry constraints on the total wave function, rotational states with even *J* (0, 2, 4, ...) can only be occupied by *p*-H_2_, and rotational states with odd *J* values (1, 3, 5, ...) can only be occupied by *o*-H_2_. These spin isomers in hydrogen are important for neutron scattering because neutrons have 1/2 spin and scatter differently from *p*-H_2_ and *o*-H_2_. The complex interaction is best described by Fermi’s golden rule, as detailed by Young and Koppel.^74^ A main conclusion of this work is that excitations by neutrons from *p*-H_2_ to *p*-H_2_ (between rotational states with even *J* values) have very small neutron scattering cross sections compared to excitations from *o*-H_2_ to either *p*-H_2_ or *o*-H_2_. This contrasting scattering behavior is illustrated in the INS spectra of solid *p*-H_2_ and solid normal hydrogen *n*-H_2_ (a mixture of *o*-H_2_ and *p*-H_2_ at a 3:1 ratio), as shown in Figure 9. For *p*-H_2_, the strongest INS peak appears at ∼118 cm^–1^ (14.7 meV) corresponding to the *J*(0 → 1) transition. Since the cross section for the *J*(0 → 0) transition is negligibly small, there is almost no elastic line or phonon band (expected at ∼50 cm^–1^) in *p*-H_2_. In contrast, the INS spectrum of *n*-H_2_ (with 75% *o*-H_2_) exhibits a strong phonon band centered at 50 cm^–1^, as well as an intense elastic line.

The elastic line, translational mode, and the *J*(0 → 1) rotational line are all extremely sensitive to the environment surrounding the hydrogen molecule. For example, the rotational energy levels can change drastically when the hydrogen is no longer a “free” 3D rotor but is subjected to an external potential. Quantitative models have been developed to connect the type/strength of the external potential and the position, splitting, and profile of the peaks.^40,75,76^ These, combined with the high penetration of the neutron beam (allowing the study of H_2_–host interactions in bulk materials or opaque sample containers) and the ability to be quantitative (the total INS intensity scales with the amount of H_2_), make INS a uniquely sensitive and powerful probe to study H_2_, such as its adsorption and activation in porous materials and on the catalyst surface. In the following, we will show a few applications related to catalysis.

**Figure 10:**
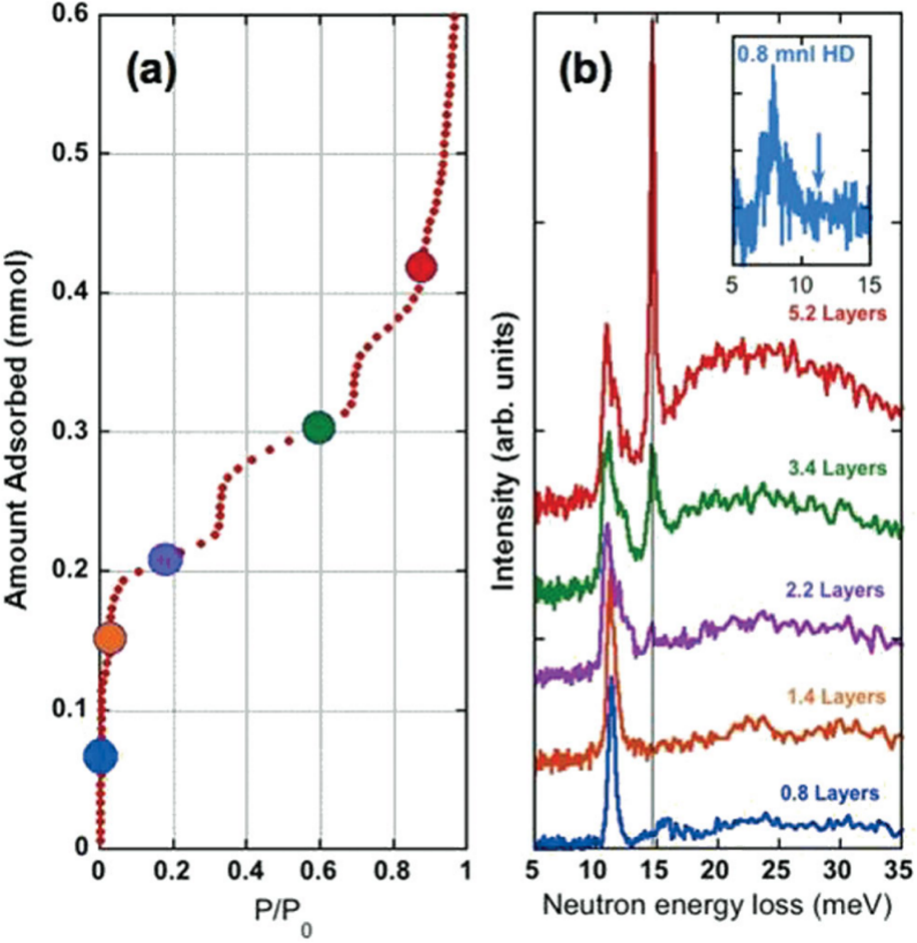
(a) Adsorption isotherms and (b) INS spectra of *p*-H_2_ on MgO(100) surface at different loading/coverage levels. Reproduced with permission from ref (77). Copyright 2008 American Physical Society.

INS has been previously used to study hydrogen on (nanoparticle) metal oxides,^77,78^ (porous) silica,^79^ zeolite,^80,81^ carbon,^82,83^ and metal–organic frameworks (MOFs).^75,84−86^ For example, Larese et al.^77^ studied H_2_ adsorbed on the MgO(100) surface and recorded a rotational energy *J*(0 → 1) of 11.25 meV (90.7 cm^–1^) when there was less than one monolayer of H_2_, significantly lower than the 14.7 meV (118 cm^–1^) free rotor. Combined with neutron diffraction and modeling data, they confirmed that this was due to the interaction of H_2_ with the Mg^2+^ ion with an effective charge *q* ∼ 1, which led to a hindered, quasi-2D rotor. As H_2_ fully covered the surface, additional H_2_ with no direct interaction with Mg gave rise to a peak at 118 cm^–1^, consistent with free rotor behavior (Figure 10)

**Figure 11:**
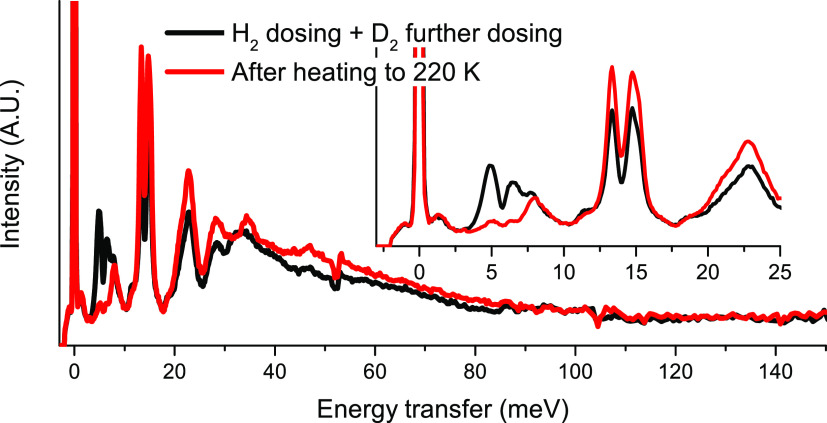
INS spectra of H_2_/D_2_ adsorbed on a Cu(I) MOF. Inset highlights the strong (∼5 meV (40.3 cm^–1^)) and weak (∼14 meV (112.9 cm^–1^)) adsorption sites, as well as the displacement of H_2_ from the strong sites to the weak sites upon D_2_ addition (note that scattering from D_2_ is an order of magnitude weaker than that from H_2_). Reproduced with permission from ref (87). Copyright 2017 Nature Publishing Group.

Hydrogen also interacts strongly with the metal sites in MOFs. In a study by Weinrauch et al.,^87^*p*-H_2_ was first dosed into a MOF with Cu(I) sites. Two binding sites were clearly observed, with the stronger site resulting in peaks near 5 meV (40.3 cm^–1^), and the weaker site having rotational excitations around 14 meV (112.9 cm^–1^) (Figure 11). Interestingly, when D_2_ was further added to the system, it preferentially binded to the strong site, displacing the H_2_ to the weaker site. The origin of this selectivity is associated with nuclear quantum effects and may have potential applications in selective activation or separation of H_2_/D_2_. Similar INS features indicating a strong interaction between H_2_ and open metal sites are also observed in other MOFs.^75,85,86^ On the contrary, there are also MOFs in which the metal sites are already saturated. Thus, H_2_ does not have direct access. In these cases, much weaker interactions between H_2_ and organic linkers or hydroxyl groups are expected, resulting in a rotational peak very close to the free rotor at 14.7 meV (118.6 cm^–1^).^84^

**Figure 12:**
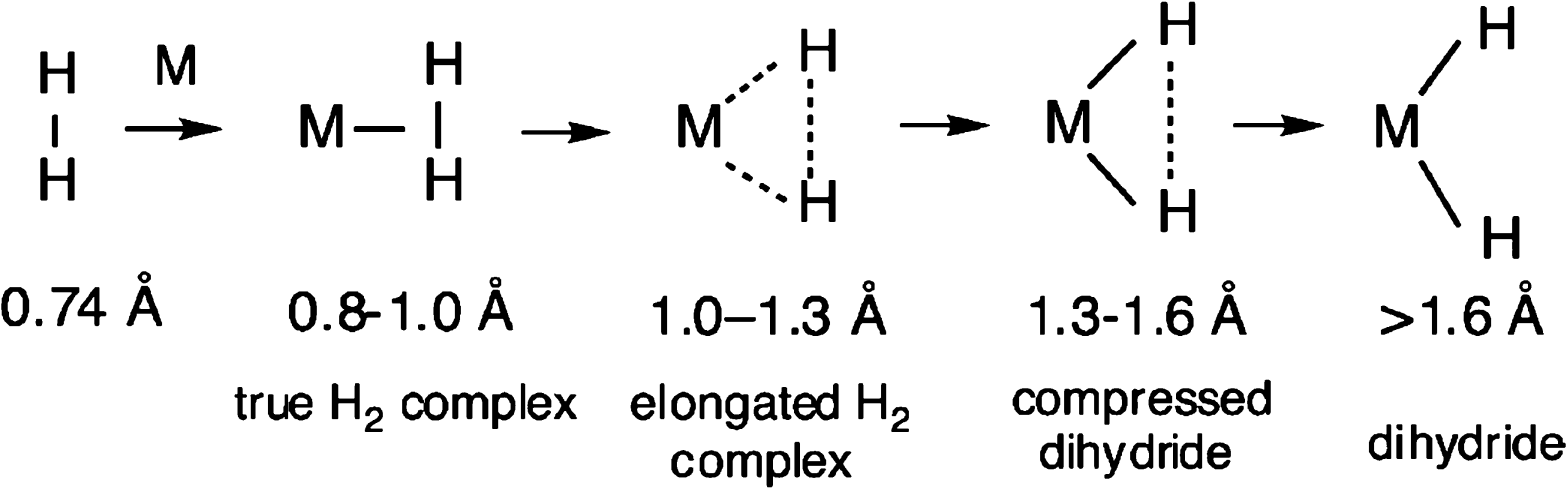
H–H distance under various situations. Reproduced with permission from ref (88). Copyright 2007 American Chemical Society.

The interaction between H_2_ and metals can be rather complicated, and the product can be found in a broad spectrum, as illustrated in Figure 12 by Kubas.^88^ Specifically, between the extremes of an H_2_ molecule and the metal dihydride, there can be multiple intermediate states depending on the relative strength of the interactions between the two H atoms and the metal site. A key indicator is the H–H distance, which, together with the environment-dependent hindrance experienced by the H_2_, will exhibit strong signatures in the INS spectra. Assignment of the peaks can lead to important information on H–H distance, nature of the complex, tunneling frequency, energy barrier, etc. In the case of W(CO)_3_(η^2^-H_2_)P_2_,^88,89^ the tunneling peaks can be explained by a quantum H_2_ rotor with an elongated H–H bond (0.82 Å) in a double-well with an energy barrier of 762 cm^–1^.

Silica, zeolites, and porous carbon are common catalysts or substrate materials for catalysts. Understanding their interaction with H_2_ is important. A study of *p*-H_2_ adsorption on Cu-ZSM-5^80^ found that the rotational excitation *J*(0 → 1) exhibited a double peak with the first peak around 12 meV (96.8 cm^–1^) and the second peak around 14 meV (112.9 cm^–1^). Similar features are also observed in other porous silica or zeolite materials.^79^ The two peaks usually go side by side, even at very low coverage, indicating that they are not due to two adsorption sites. The integrated intensity of the first peak is about twice that of the second peak. A reasonable explanation is that the first peak is due to excitation from *J*_0_*M*_0_ to *J*_1_*M* ± 1 (*M* is the magnetic quantum number and in the free rotor has a degeneracy of (2*J* + 1)). In contrast, the second peak is due to excitation to *J*_1_*M*_0_. This suggests that the hydrogen is likely parallel to the surface and has a slightly reduced rotational constant (meaning a slightly compressed H–H distance).

In brief, this section summarizes the basic theory of INS from dihydrogen, as well as the applications of INS to understand the status of H_2_ adsorbed on different porous materials and surfaces. The strength and advantages of INS are prominent, and it is arguably the most powerful technique available to understand adsorbed dihydrogen.

#### 3.2.2 Metal Hydrides in Ammonia Synthesis and Other Reactions

The cost of CO_2_ emissions in the Haber–Bosch process for ammonia synthesis has resulted in intensive efforts to find alternative catalysts.^90,91^ In 2000, it was reported^92^ that ternary nitrides Fe_3_Mo_3_N, Co_3_Mo_3_N, and Ni_2_Mo_3_N were active catalysts, particularly when doped with Cs. The materials have been studied by *in situ* neutron powder diffraction.^93,94^ Computational studies suggest two mechanisms^95^ are operative: one involves the direct reaction between surface-bound activated N_2_ and H_2_; in the second, H_2_ dissociates on a Co_8_ cluster and reacts with N atoms on the surface. Presumably, the role of Cs is to promote the dissociation of H_2_.

**Scheme 1:**
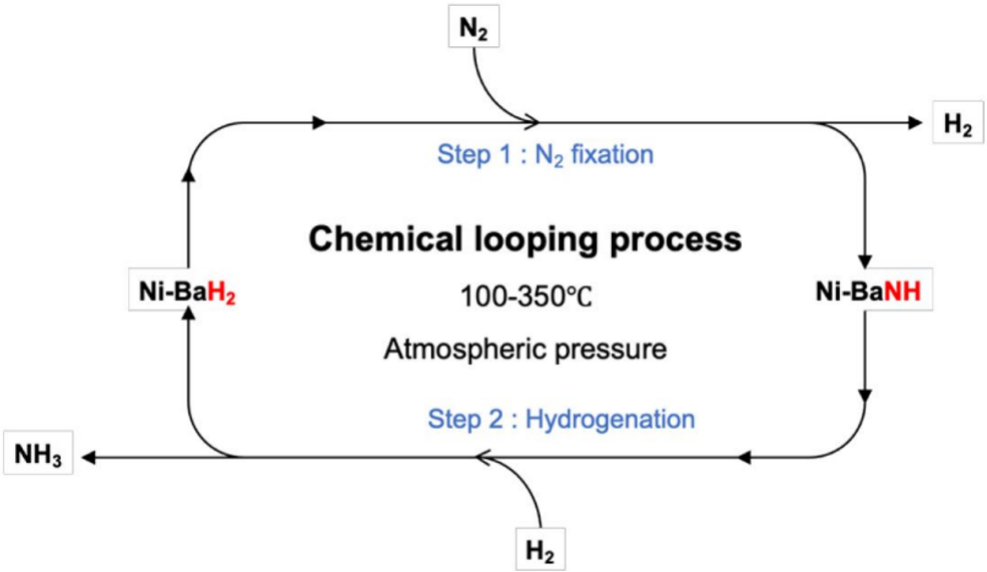
Proposed Mechanism for the Chemical Looping Process for Ammonia Synthesis over Ni/BaH_2_ Reproduced with permission from ref (101). Copyright 2021 Springer.

Recent work has shown that alkali metals or alkaline earth hydrides, combined with transition metals, are active ammonia synthesis catalysts.^96^ The group I and II hydrides have been comprehensively characterized by INS spectroscopy and DFT calculations,^97−99^ as have many ternary metal hydrides.^100^ These catalysts are operated in a chemical looping mode involving alternating cycles of nitrogen fixation and hydrogenation, as illustrated schematically in Scheme 1. An INS and *in situ* neutron powder diffraction study^101^ of Ni/BaH_2_ as a model catalyst for ammonia synthesis clearly showed the cycling between BaNH and BaH_2_. Such systems are ideally suited to neutron scattering, and there are clearly opportunities in this area.

**Figure 13:**
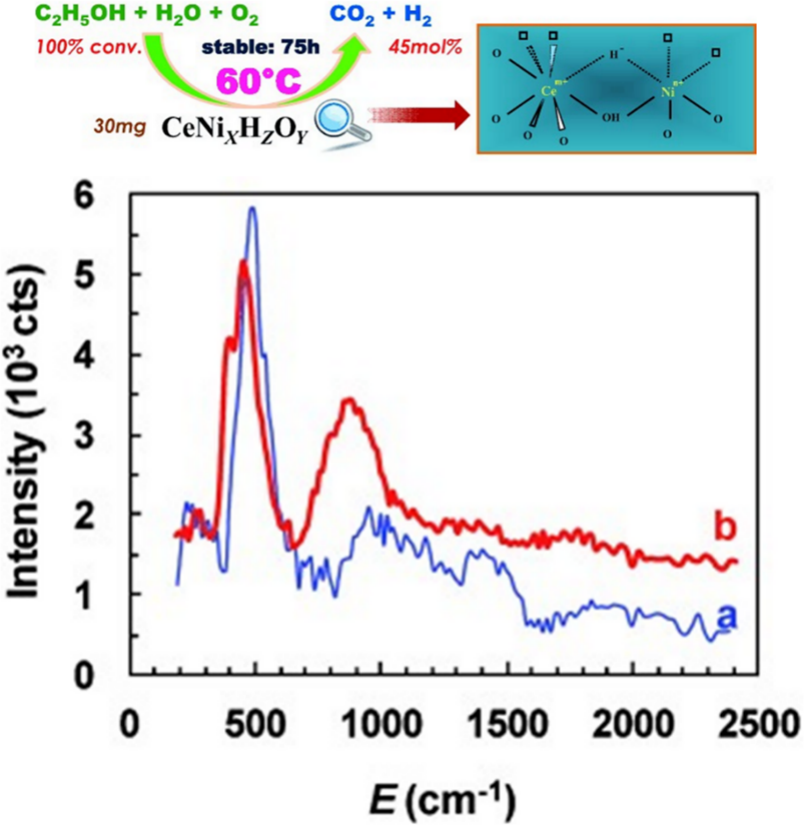
Top: schematic of ethanol reforming using a CeNi_*x*_H_*y*_O_*z*_ oxyhydride catalyst. Bottom: background subtracted INS spectra of CeNi_*X*_H_*Z*_O_*Y*_ catalysts obtained after treatment in H_2_ at 250 °C: (a) *X* = 0.5 and (b) *X* = 1. Reproduced with permission from ref (110). Copyright 2013 Wiley-VCH.

A novel development in materials chemistry has been the discovery of oxyhydrides.^102^ The archetype is BaTiO_3_H_*x*_ that contains both O^2–^ and H^–^ ions. Several of the parent compounds have been investigated by INS.^103−106^ These materials have been proposed as catalysts or catalyst supports for various reactions, including ammonia synthesis,^107^ selective hydrogenation,^108^ and steam reforming of ethanol.^109^ The last of these has been extensively investigated by INS using a CeNi_*x*_H_*y*_O_*z*_ catalyst.^109−112^Figure 13 shows a schematic of the reaction and depiction of the active site. The INS spectra are assigned as the hydride (peak at 460 cm^–1^) and hydrogen chemisorbed on nanoparticulate Ni (peak at 870 cm^–1^).

#### 3.2.3 Hydrogen on/in Metals

While most of the transition metals form binary hydrides of the hydrogen-in-metal type,^113^ albeit about half of them only do so at high pressure,^114^ for applications in heterogeneous catalysis, it is the formation of surface hydrides that is generally required.^115^ Industrially, the most important ones are Ni, Pd, Pt (hydrogenation catalysts), Fe (ammonia synthesis and Fischer–Tropsch synthesis), Co (Fischer–Tropsch synthesis), and Cu (methanol synthesis).

Hydrogen bound to a surface is difficult to detect by vibrational spectroscopy. Raman spectroscopy is usually hampered by fluorescence; infrared spectroscopy suffers from the bands being intrinsically weak and, for supported catalysts, often from a limited spectral range because of absorption by the support. The metal surface selection rule restricts both forms of spectroscopy: only modes that involve motion perpendicular to the surface are allowed.^116^ All these factors are irrelevant to INS spectroscopy: metals and supports are essentially transparent to neutrons (so the entire 0–4000 cm^–1^ range is observable), and there are no selection rules. The disadvantages of INS are that it is insensitive and usually measured at 20 K or less. INS spectroscopy requires 0.1–10 mmol H (depending on the instrument) in the beam. Generally, for metal surfaces, at best, there is a 1:1 ratio of hydrogen to surface atoms; this means that to obtain sufficient hydrogen in the beam, high surface area materials are essential. Thus, the samples are usually either supported metals, where the metal loading is at least 5 wt %, or (relatively) high surface area (10–80 m^2^ g^–1^) metals, most commonly Raney-type or metal blacks (skeletal metals). Work before 2005 is reviewed elsewhere,^40^ so the focus here will primarily be on work since then.

The metals that have been studied by INS are those that form surface hydrides under ambient conditions, and for this reason, almost all of the studies have been of Ni, Pd, and Pt. Hydrogen on these metals has been studied many times^40^ and, notably, the spectra are consistent between different groups at various institutions across the decades.

##### 3.2.3.1 Nickel

**Figure 14:**
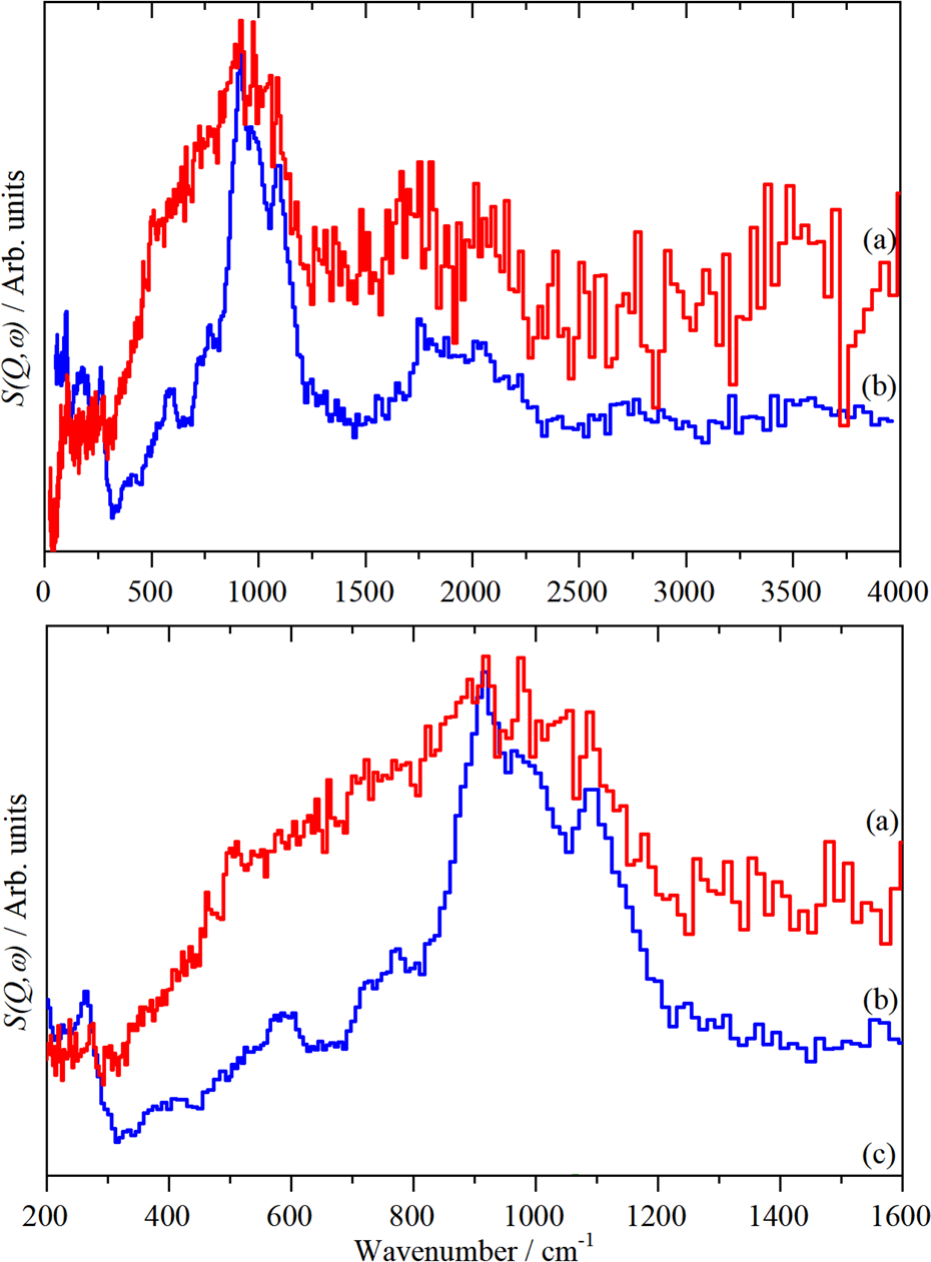
Difference INS spectra of hydrogen on: (a) Raney Ni (blue) and (b) the Ni foam sample (red). Reproduced with permission from ref (119) under a Creative Commons Attribution 4.0 International License (CC-BY).

Figure 14a shows the INS spectrum of hydrogen on Raney Ni,^117^ which is typical of that usually found.^118^ Shown in Figure 14b is the INS spectrum of hydrogen on a novel type of Ni foam catalyst.^119^ This evolution of the Raney process results in a highly porous, lightweight material.^120^ It is clear that the spectra are markedly different. In Figure 14b, there are hints of peaks at 900 and 1030 cm^–1^, which by comparison to Raney Ni, are assigned to hydrogen on (111) facets. The major difference is the greater intensity in the 400–800 cm^–1^ region. This is assigned to hydrogen on “non-(111)” facets. The intensity shows a much larger degree of surface heterogeneity in this sample than in Raney Ni. A crude estimation, based on the relative area of hydrogen on the (111) sites to the total area, would indicate that there are approximately equal numbers of (111) and non-(111) sites in the foam catalyst. In contrast, for Raney Ni, the ratio is at least 5:1, (111) to non-(111).

##### 3.2.3.2 Palladium

**Figure 15:**
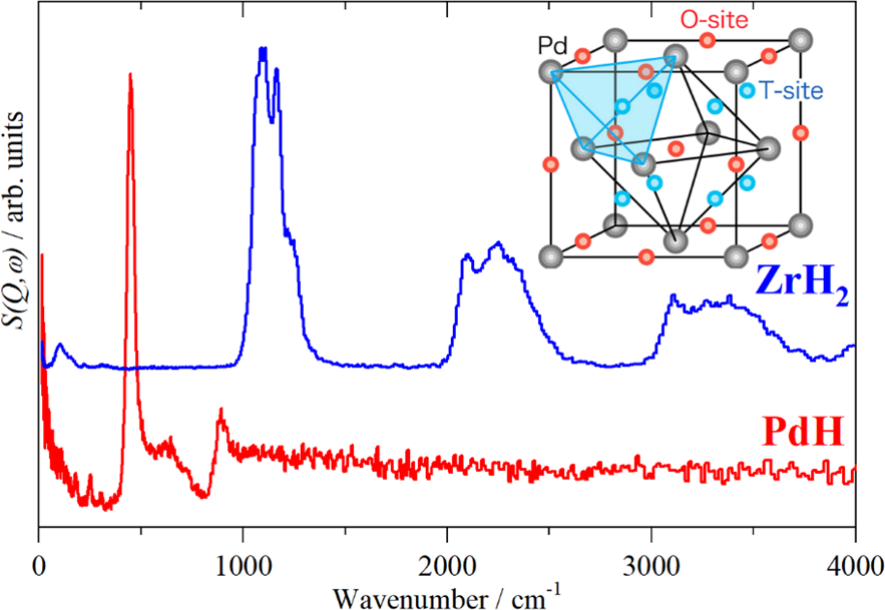
INS spectra of PdH and ZrH_2_. Inset: the octahedral (O-site) and tetrahedral (T-site) sites available in an fcc solid. The inset is reproduced with permission from ref (125). Copyright 2016 American Chemical Society.

Palladium is exceptional in readily absorbing hydrogen at room temperature to form the archetypal hydrogen-in-metal system.^121^ The stoichiometry is PdH*x* where 0 ≤ *x* ≤ 1. There are two phases, α (*x* ≤ 0.017) and β (0.60 ≤ *x* ≤ 1), and the two phases coexist in the intermediate regime. The α-phase is a solid solution of hydrogen in Pd, and the β-phase is an ordered structure.^122^ Ignoring imperfections, in a face-centered cubic (fcc) metal such as Pd, there are two possible sites for hydrogen to occupy: octahedral (O-site) and tetrahedral (T-site) (see Figure 15, Inset). In bulk PdH, the longer Pd–H distance of the octahedral site means that this is the preferred site for both the α- and β-phases. INS spectroscopy is consistent with this,^123^ as the 0 → 1 (fundamental) transition in PdH is at ∼500 cm^–1^, whereas in ZrH_2_, where the tetrahedral site is occupied,^124,125^ the fundamental transition is at ∼1050 cm^–1^ (Figure 15).

**Figure 16:**
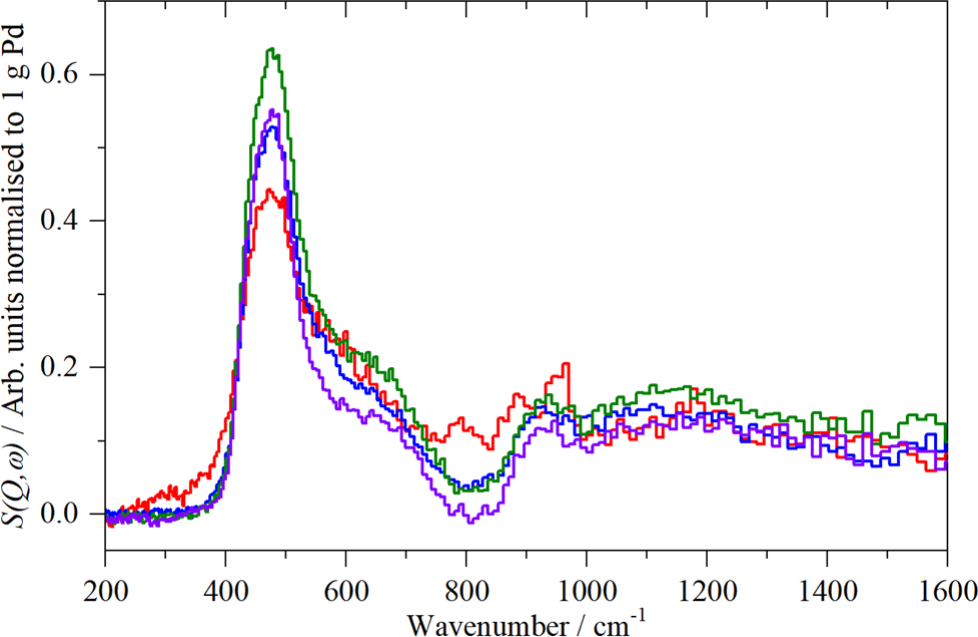
Normalized difference INS spectra of a series of carbon supported Pd catalysts after subtraction of the same sample evacuated at 200 °C overnight. Key: sample 1 (red) Pd(20%)/activated carbon, sample 2 (blue) Pd(20%)/carbon black, sample 3 (olive) sample 2 heated to 300 °C in argon, and sample 4 (violet) sample 2 heated to 400 °C in argon. Reproduced with permission from ref (129) under a Creative Commons Attribution 3.0 Unported License (CC-BY).

The hydrogenation activity of Pd is dependent on the availability of hydrogen. This is clearly seen in the Lindlar catalyst^126^ (5%Pd+3.5%Pb/CaCO_3_). This stereoselectively hydrogenates alkynes to *cis*-alkenes and is a key reagent in the production of vitamin A.^127^ INS spectroscopy shows that under 1 bar H_2_ pressure, the Lindlar catalyst retains 2.2 times less hydrogen than the equivalent Pd-only catalyst (5% Pd/CaCO_3_).^128^ The amount of β-PdH formed strongly depends on the catalyst morphology, which is largely determined by the support choice and synthesis conditions. This is apparent in the INS spectra of a series of carbon supported Pd catalysts^129^ as shown in Figure 16, and the relative areas of the region 350–800 cm^–1^ are shown in Table 2. The shape and intensity of the 0 → 1 transition of β-PdH at ∼500 cm^–1^ differ.

**Table 2:** Integrated Hydrogen Areas from the IINS Spectra in Order of Increasing Average Primary Particle Size^a^

Catalyst (sample no.)	Morphology^b^	DN/nm^c^	Relative area^d^
Pd(20%)/carbon black (2)	agg	2.40	1.31
Pd(20%)/activated carbon (1)	prim	3.58	1.00
Pd(20%)/carbon black 300 °C (3)	agg/prim	6.73	1.65
Pd(20%)/carbon black 400 °C (4)	prim	7.74	1.30

a Reproduced with permission from ref (129) under a Creative Commons Attribution 3.0 Unported License (CC-BY).

b Morphological differences according to TEM: agg = mostly aggregates, prim = mostly isolated primary particles.

c Average particle size from statistical determination by TEM.

d Relative area of the region 350–800 cm^–1^ of the spectra shown in Figure 16.

The influence of the support and the effect of alloying were also seen in a series of catalysts tested for hydrogenation of aromatic nitro compounds, a crucial step in the production of isocyanates for polyurethane manufacture.^130^ In the series Pd(5%)/C, Pd(4.5%)Pt(0.5%)/C, and Pd(4.5%)Pt(0.5%)Fe(5%)/C, the relative amount of hydrogen (normalized to 1 g Pd) is 4.7:3.33:1.00, respectively. This approximately correlated inversely with the activity seen in a model reaction (nitrobenzene hydrogenation): 6.6, 36.9, 23.1 mmol nitrobenzene min^–1^. A more detailed discussion of the influence of the support and the effect of alloying on morphology and hydrogen capacity is given elsewhere.^131^

**Figure 17:**
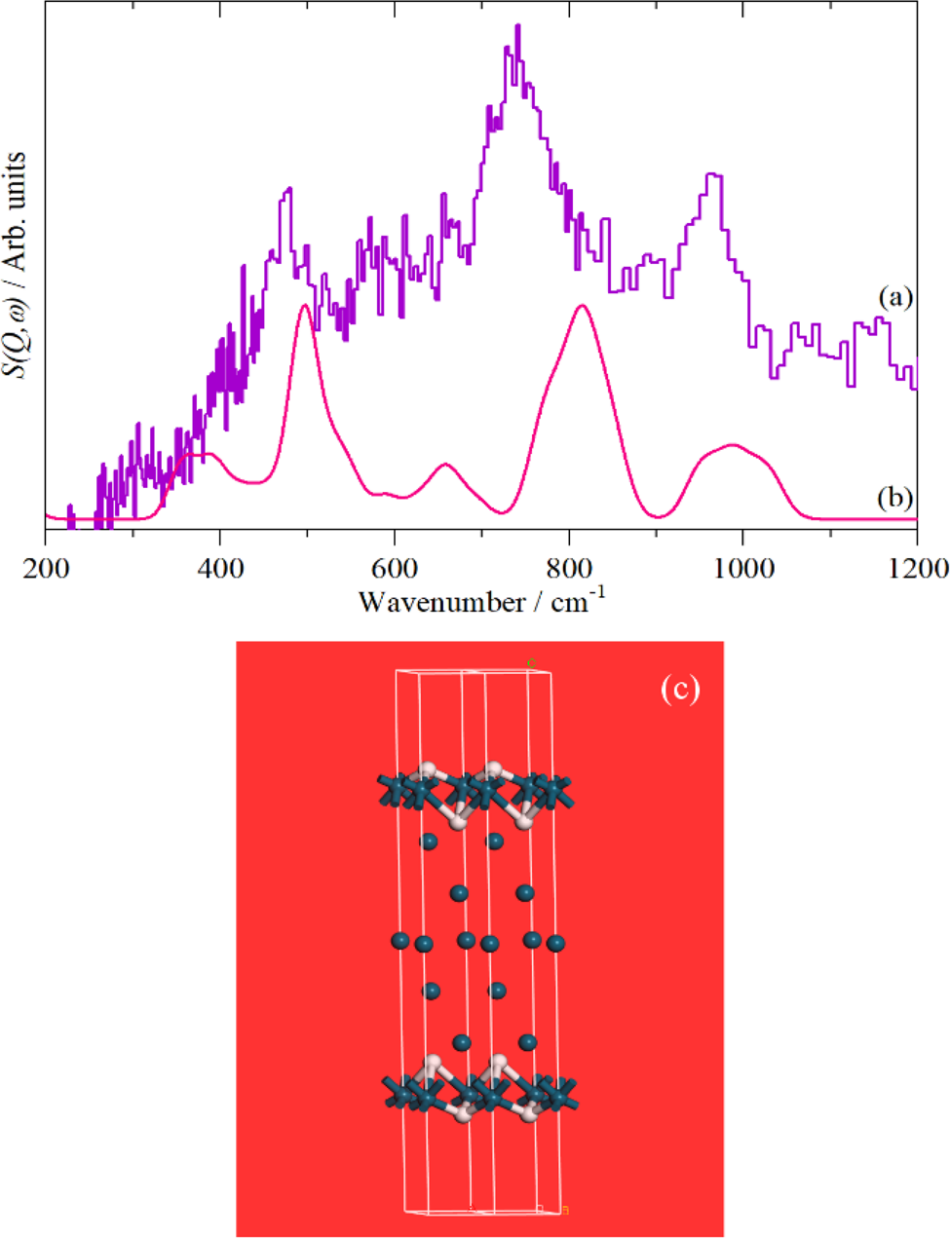
(a) INS spectrum of Pd black after dehydrogenation at 100 °C, (b) calculated INS spectrum based on the model shown in panel (c). Reproduced with permission from ref (129) under a Creative Commons Attribution 3.0 Unported License (CC-BY).

At very low hydrogen concentrations, it is possible to detect surface-bound hydrogen. Figure 17a shows the INS spectrum of a Pd black after dehydrogenation at 100 °C. The key role of modeling in neutron scattering was emphasized earlier in this review. Figure 17b shows the results of a DFT calculation for the model shown in Figure 17c. The model has hydrogen in 3-fold coordination sites at the surface, which results in modes at 815/988 cm^–1^ (738/966 cm^–1^) and in subsurface sites that result in the 496 cm^–1^ (476 cm^–1^) mode (experimental values in brackets). The width of the calculated peaks is because of strong vibrational dispersion (variation of transition energy with wavevector) in the modes.

While the state of the hydrogen in β-PdH has been comprehensively studied, the nature of the hydrogen at the surface has been almost completely neglected by NVS: we are unaware of any experimental studies in this area. By using an INS spectrometer that can be optimized^31^ to look for modes at ∼2000 cm^–1^, it was possible to detect a weak vibration at 2150 cm^–1^ that was assigned to hydrogen in the on-top site (i.e., bonded to a single metal atom).^129^

Differences in the activity and selectivity of α- and β-phase hydride in the selective hydrogenation of ethyne are known,^132^ and this may be related to the presence or not of the on-top surface site. The on-top site is undoubtedly populated under conditions where H_2_ gas is present, so it is likely a hitherto unrecognized participant in catalytic hydrogenations by β-PdH.

**Figure 18:**
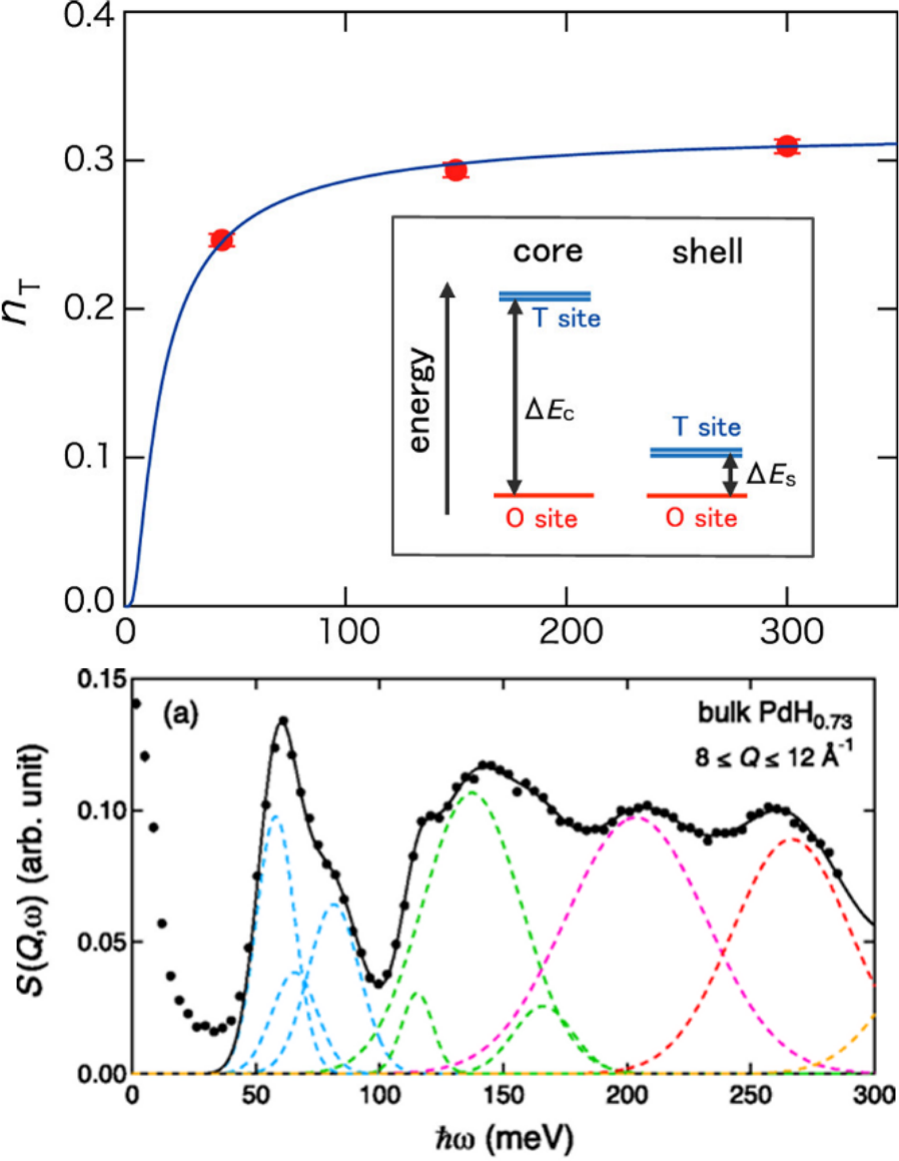
Upper: temperature dependence of the fraction of the D atoms at the T-sites (*n*_T_) determined by Rietveld analysis of PdD_*x*_.^125^ Lower: INS spectrum of nano-PdH_0.42_ at 10 K.^135^ The difference between H adsorption in the bulk (dashed line) and the nanoparticles (solid line with error bars) is shown at the bottom of the figure as a series of Gaussian bands. These are assigned as hydrogen in T-sites. Upper image reproduced with permission from ref (125). Copyright 2016, American Chemical Society. Lower image reproduced with permission from ref (135). Copyright 2017 American Physical Society.

Earlier, we had stated that hydrogen occupies the O-site in the Pd bulk. Lately, this view has been challenged. Based on neutron diffraction studies of Pd nanoparticles, two groups^125,133^ have proposed that there is a significant occupation of the tetrahedral sites, particularly near room temperature (Figure 18 upper part). A third group^134^ claims to have imaged the interstitial hydrogen atoms at the near-surface region of octahedral PdH_*x*_ nanoparticles by scanning TEM. The absorbed hydrogen occupies the T-site interstices near the surface, while the occupation gradually changes to the O-site in the bulk. DFT calculations show that the absorption energy difference between T-site and O-site hydrogen becomes much smaller at the subsurface than in bulk Pd and can be further reduced at the subsurface of PdH. An INS study^135^ found excess intensity in the spectra of nanoparticulate PdH_0.42_ as compared to the bulk (Figure 18 lower part) that was assigned to hydrogen in T-sites. The authors also found additional intensity around 2000 cm^–1^ that was not accounted for within their model and suggested that this may be surface species. This would be consistent with the earlier study.^129^ QENS of the same nanoparticles used for Figure 18 (lower part) showed an additional fast process not seen in the bulk, which was interpreted as jumps between T-sites.^136^ The O- and T-sites are energetically distinct, so they presumably have different reactivity that may influence their properties. This topic merits further investigation.

##### 3.2.3.3 Platinum

In marked contrast to palladium, the hydrogen solubility in platinum is essentially zero, so the hydrogen is entirely at the surface. Hydrogen readily dissociates on platinum at room temperature, but the temperature range over which this occurs was unknown. This can be investigated by QENS. An elastic window scan of a Pt(50 wt %)/C fuel cell catalyst that was loaded with H_2_ at 20 K^137^ showed three regions: the decrease in signal in temperature below 60 K corresponded to desorption of physisorbed H_2_, the increase in signal in the temperature range 60–125 K was because of the dissociation of H_2_ and consequent binding of hydrogen to the surface. The slow decrease above 150 K was the usual behavior as the Debye–Waller factor increases. The data clearly shows that H_2_ dissociates on Pt over the range 60–120 K.

INS is a relatively insensitive technique, so to maximize the signal, either Pt black or high metal loading (40–60 wt % Pt) supported catalysts have been used. Improvements in instrumentation mean that 5 wt % Pt catalysts can now be studied.^138−140^ Remarkably, it is possible to observe both the expected Pt–H modes and molecular hydrogen physisorbed to Pt–hydride species.^138^

**Figure 19:**
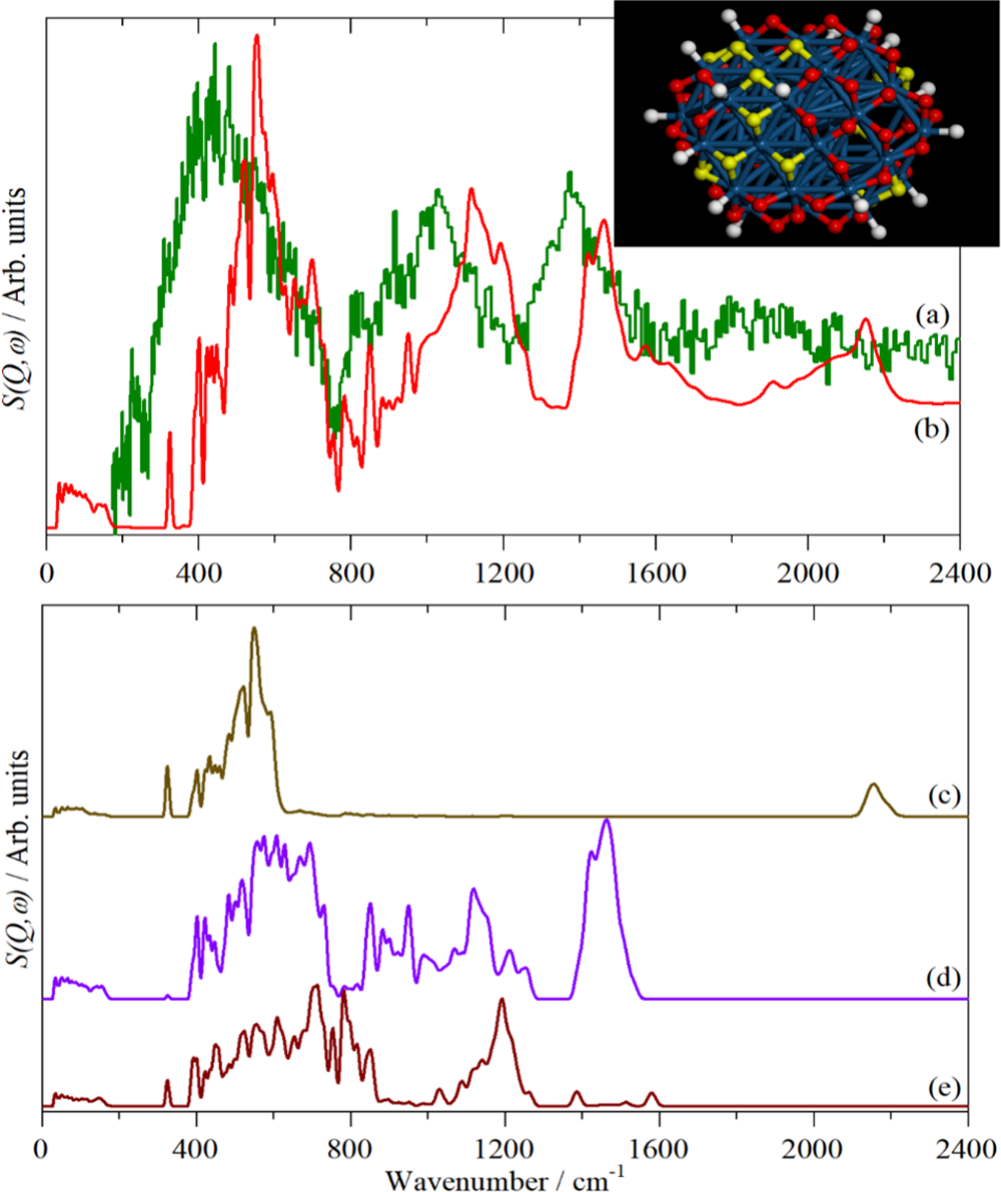
Comparison of: (a) the experimental INS spectrum of hydrogen on platinum black (olive) recorded on IN1-Lagrange with that calculated by (b) lattice dynamics (red) for the Pt_44_H_80_ nanoparticle shown in the inset. (On-top hydrogen are shown in white, 2-fold hydrogen in red and 3-fold hydrogen in yellow.) Contributions from the different sites to the total spectrum of the Pt_44_H_80_ nanoparticle: (a) on-top, (b) 2-fold, and (c) 3-fold. Reproduced with permission from ref (141) under a Creative Commons Attribution 4.0 International License (CC-BY).

The assignment of the surface sites occupied by hydrogen on platinum has been controversial for decades.^40^ The INS spectra are remarkable because the overall profile is almost independent of the environment. Hydrogen on Pt black,^141,142^ on Pt in zeolite Y,^142^ on Pt on carbon,^137^ silica,^142^ or alumina supports^138−140^ all give essentially the same spectra. DFT calculations of hydrogen on a Pt nanoparticle^141^ and alumina supported Pt clusters^140^ have finally resolved the debate. Figure 19 shows a comparison of the INS spectrum of hydrogen on Pt black and that calculated for a Pt_44_H_80_ nanoparticle (inset in the figure). Apart from a small shift to higher energy, the calculation is in outstanding agreement with the experimental data. Calculated INS spectra can be readily decomposed into individual contributions, and Figure 19b–d shows the contributions of the on-top site, the 2-fold bridge, and the 3-fold site. No 4-fold coordinated H atoms exist because the Pt–H distance is too long. Instead, hydrogen forms 2-fold bridges around the edges of the 4-fold site, and there is a complete absence of subsurface hydrogen, consistent with the vanishingly small solubility. It had been assumed that the 3-fold site would be dominant because (111) was the lowest energy surface of Pt. This work shows that this is not the case, and the major contributor is the 2-fold site. The work on the supported clusters gave the same results: the best fit to the experimental data was a linear combination of models that mainly had 2-fold sites.^140^

One noticeable outcome of the work is that both analyses show the H:Pt ∼ 2. Hydrogen chemisorption measurements to determine the metal dispersion have always assumed H:Pt = 1. This work demonstrates that this is not a reasonable assumption.

##### 3.2.3.4 Cobalt

Cobalt-based catalysts are becoming increasingly important for low temperature Fischer–Tropsch synthesis of long-chain hydrocarbons from syngas (H_2_ + CO).^143−145^ A key step in the reaction is the dissociation of H_2_ on cobalt. Surprisingly, studies by vibrational spectroscopy of hydrogen adsorbed on Co are extremely scarce. There is one comprehensive surface science study of hydrogen on Co(1010)^146^ and two INS studies that used Raney Co.^147,148^ Recent work^119^ has shown that both of these are flawed: bands that were assigned as Co–H modes are, almost certainly, deformation modes of hydroxyls. Distinguishing between hydroxyls and metal–hydrogen modes with the type of spectrometer (“indirect geometry”) commonly used for molecular spectroscopy is difficult. While these give excellent spectra below 2000 cm^–1^ (most of the INS spectra shown in this review were recorded with this type of spectrometer), their design means that data in the C–H/N–H/O–H stretch region (2500–4000 cm^–1^) is unreliable (as explained in more detail elsewhere^31^). The complementary use of a different type of spectrometer (“direct geometry”) that enables the high-energy region to be studied reliably is essential, as the presence or absence of an O–H stretch mode allows an unambiguous distinction between hydroxyls and metal–hydrogen modes.

**Figure 20:**
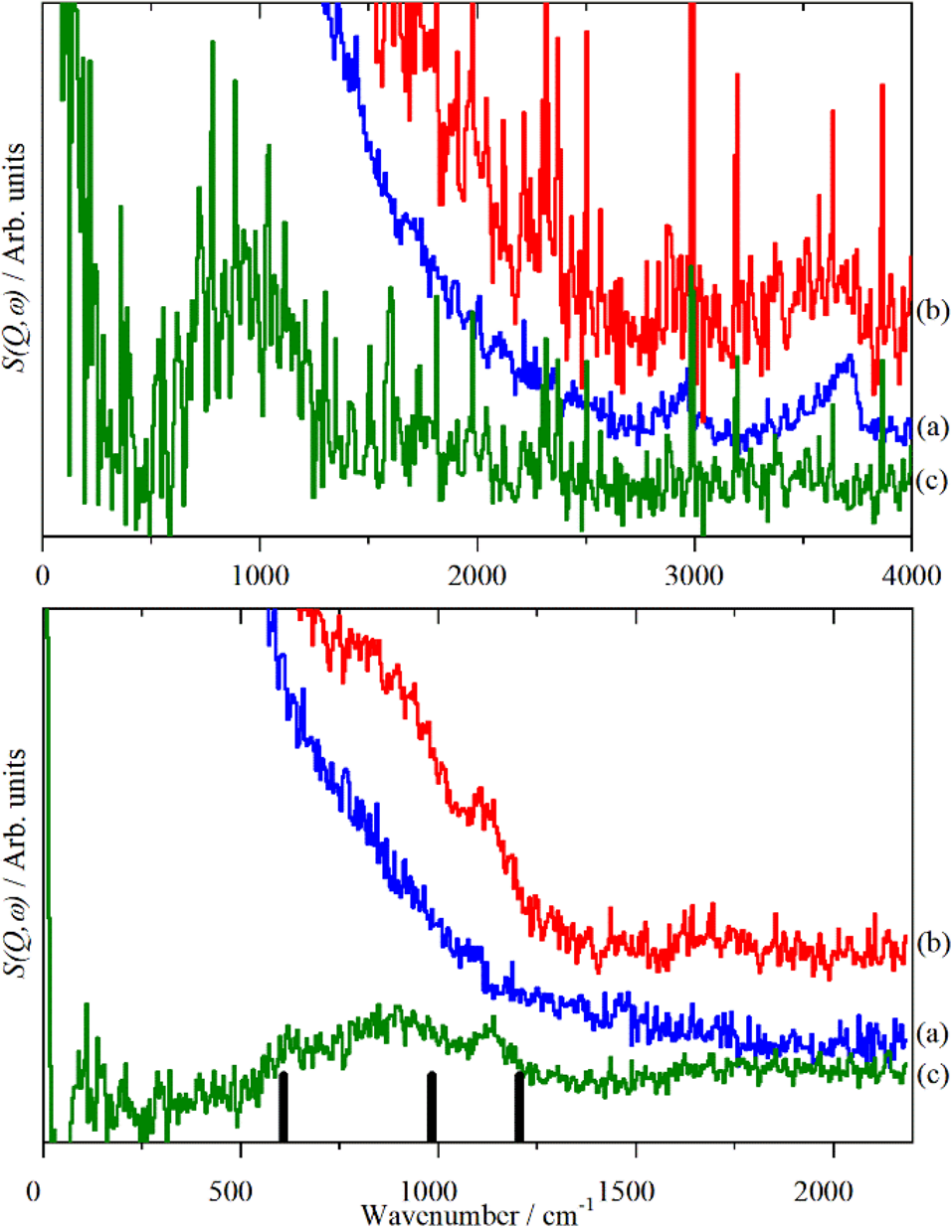
INS spectra of Raney Co, all spectra recorded with a direct geometry spectrometer (MAPS). Top: *E*_*i*_ = 650 meV (5243 cm^–1^), bottom: *E*_*i*_ = 300 meV (2420 cm^–1^). (a) Dried, reduced sample, (b) sample plus 1 bar H_2_, and (c) the difference spectrum (a–b). The vertical bars in the lower part are the transition energies at which the Co–H modes are found for the high coverage phase of hydrogen on Co(1010). Reproduced with permission from ref (119) under a Creative Commons Attribution 4.0 International License (CC-BY).

Figure 20 shows spectra for hydrogen on Raney Co recorded with a direct geometry instrument.^119^ The difference spectrum (Figure 20c) shows a broad band centered at ∼880 cm^–1^. Crucially, the difference spectrum shows no change in the O–H stretch region on the addition of hydrogen, so the feature at 880 cm^–1^ cannot be hydroxyls. Comparison with the transition energies found for the high coverage phase of hydrogen on Co(1013)^146^ shows that the envelope encompasses the modes and that there are submaxima at, or close to, the Co–H modes found for Co(1010).

The work showed that an oxidic and/or hydroxylated Co surface is very resistant to reduction. An extended reduction period at >250 °C is required to remove most hydroxyls. It is also clear that the clean surface is highly reactive: even very small amounts of oxygen (e.g., as found in a typical glovebox) result in the hydroxylation of the surface.

Recently, INS proved that the existence of oxygen vacancies on Co_3_O_4_ played an important role in the formation of hydride.^149^ The activation of H_2_ over Co_3_O_4_ at 250 °C formed Co–H species at 110 meV (887.3 cm^–1^) and Co–OH moieties evidenced by a broad feature around 80–160 meV (645.3–1290.6 cm^–1^). However, the activation of H_2_ over metallic Co did not yield features of Co–H due to the absence of Co–O and oxygen vacancies. DFT calculations indicated that H_2_ underwent both homolytic and heterolytic dissociation over CoO(100)–O_V_ sites to yield hydride species.

##### 3.2.3.5 Copper

Copper is a key component in the Cu/ZnO/Al_2_O_3_ catalyst used for the industrial manufacture of methanol.^150^ As discussed in more detail in the section on methanol synthesis (Section 3.3.1), copper(I) hydride, CuH, has been proposed as a hydrogen reservoir in the reaction.

CuH was the first binary metal hydride to be discovered (in 1844)^151^ and has been characterized by neutron diffraction^152−155^ and INS spectroscopy.^154,155^ The diffraction studies show that while the stoichiometric material can be made, it is generally nonstoichiometric, CuH*x*, with *x* ∼ 0.75. There are aqueous and nonaqueous routes to make CuH*x*. The resulting materials have different properties, particularly regarding solubility. A combination of total scattering neutron diffraction, INS spectroscopy, and DFT calculations showed that the products from both routes were nanoparticulate with a core of CuH*x* but with different surface termination: bonded hydroxyls for the aqueous routes and a coordinated donor for the nonaqueous routes.

CuH*x* provides a particularly clear example of how the nature of an adsorbed layer on a nanoparticle surface determines its properties. Functionalization and optimization of nanoparticles by manipulating the surface layer is a topic of considerable interest,^156^ as it potentially provides a means to tailor the properties of the system. INS spectroscopy is well-suited to characterize hydrogenous adlayers on nanoparticles,^157^ as the optical absorption that hampers conventional spectroscopy is irrelevant.

#### 3.2.4 Hydrogen on/in Oxides

##### 3.2.4.1 Hydride Species: H^–^

When exposed to H_2_, surface hydrides could exist on the surface of metal oxides. Generally, H^–^ and H^+^ species from the heterolytic split of H_2_ can form a surface hydride (M–H, M is the metal cation) and a hydroxyl (OH), respectively, on nonreducible metal oxide surfaces (e.g., MgO, Al_2_O_3_, ZnO). Two hydrogen atoms (H·) from the homolytic split of H_2_ can reduce a reducible metal oxide surface (e.g., CeO_2_, TiO_2_) and generate two hydroxyls. The formation of surface hydrides is not limited to nonreducible metal oxides. For instance, neutron-based experiments find that a hydride could be stabilized on the surface of reducible metal oxides when surface defects are present.^158^ In addition to surface defects, the electronic properties (e.g., polarization of the metal–O bond, bond strength of metal–H, reducibility, band gap) of metal oxides could also affect the formation mechanism of surface hydrides.^159,160^ Among all metal oxides that can form hydrides from H_2_, CeO_2_ is the most intensively studied.^161,162^ Over CeO_2_, it was found that the homolytic splitting of H_2_ was thermodynamically favored, whereas the heterolytic pathway was kinetically preferred. The results obtained from neutron techniques showed that hydride could exist on the ceria surface, although there were debates on the formation pathway. This section will primarily focus on hydride formation on the ceria’s surface and discuss the results obtained from neutron-based techniques. Reports of hydrogen on other metal oxides (e.g., ZnO) are scarce and will also be summarized.

**Figure 21:**
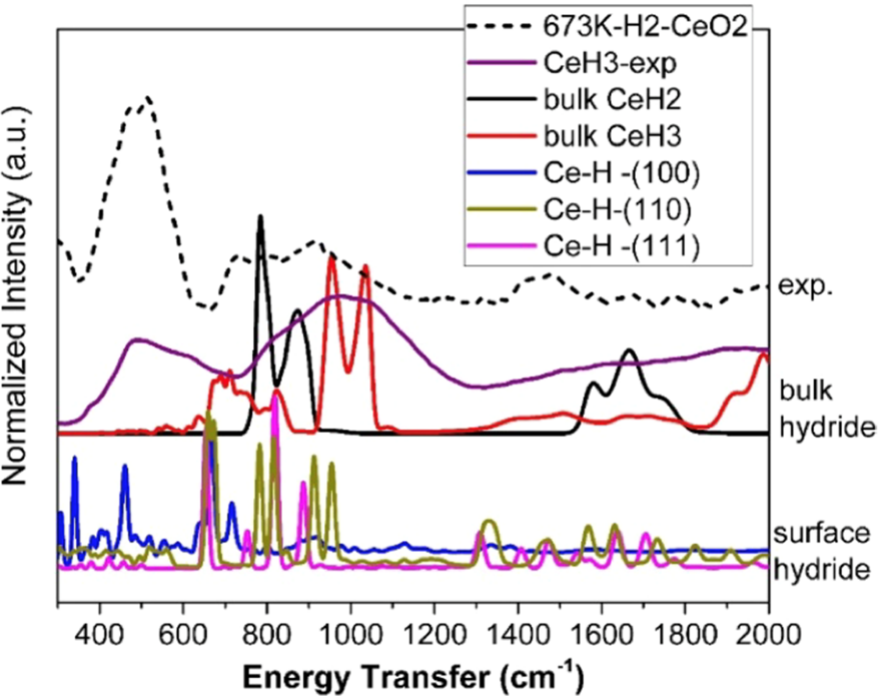
Simulated INS spectra of bulk hydride of CeH_2_ and CeH_3_ and surface hydride on reduced (111), (110), and (100) surfaces. The experimental spectra from CeO_2_ after H_2_ treatment at 400 °C and bulk CeH_3_ are also shown for comparison. Reproduced with permission from ref (164). Copyright 2017 American Chemical Society.

Using INS, Lamonier et al. observed peaks attributed to hydride species (a peak at 490 cm^–1^) and surface hydroxyl groups (peaks at 100, 280, and 660 cm^–1^) on reduced cerium–nickel oxides.^163^ In addition to the surface hydride and the hydroxyl, several experimental and theoretical studies suggest that −OH and M–H species can be formed in the subsurface and bulk region of ceria due to the migration of surface H adatoms. Via *in situ* INS spectroscopy, Wu et al. found the existence of bulk Ce–H species in addition to surface hydride after heterolytic dissociation of H_2_ on ceria.^164^ However, the −OH groups in the bulk phase of CeO_2_ were not detected.^164^ It could be that bulk hydroxyl was unstable and destabilized as the amount of oxygen vacancies increased.^165^ Specifically, as shown in Figure 21, three groups of peaks were detected in INS spectra. Consistent with results obtained by Lamonier et al., sharp peaks at 400–650 cm^–1^ (B1) were assigned to the deformation band of surface Ce–H with a possible contribution from bulk CeH_3_-like species. Broad peaks at 750–1100 cm^–1^ (B2) were attributed to the deformation band of bulk Ce–H from CeH_2_ and/or CeH_3_-like species. The combinations and/or overtones of the B1 and B2 peaks were observed at 1300–1800 cm^–1^.

Interestingly, products derived from homolytic splitting (surface–OHs) were observed on a close-to-stoichiometric CeO_2_ surface. In contrast, heterolytic products (Ce–H and −OH) were detected on the CeO_2_ surface with oxygen vacancies.^164^ These experimental results suggested that oxygen vacancies participated in the formation of hydride on the surface of ceria via heterolytic splitting of H_2_ and the proposed pathway was: H_2_ + O^2–^ + □ → OH^–^ + H^–^ (□ = anionic vacancy). The hydride formed on the surface of ceria via heterolytic split of H_2_, based on both experimental and theoretical results, could transform into hydroxyl in the presence of O_2_, under which the oxygen vacancies were filled, leading to the transfer of H to the lattice O, the generation of −OH, and the reduction of two Ce^4+^ cations.^159,161,162,166^ The proposed mechanism was: 2H^–^ + O_2_ → 2OH^–^.

Apart from neutron scattering studies, recent work proposed new pathways for hydride formation over CeO_2_,^161,167,168^ with the results in general agreement with the INS results. In one pathway, the *4f* electron from Ce^3+^ could be transferred to hydrogen on the subsurface of CeO_2_ with defects to form hydride species and oxidize Ce^3+^ to Ce^4+^. The process was depicted as H_2_ + 2Ce^3+^_Vo_ → 2Ce^4+^_Vo_–H^–^ and could occur because the energy level of the localized *4f* electron of Ce^3+^ was relatively high.^167,169^ In addition, Wang et al. found that the H^–^ species originated from H_2_ heterolytic dissociation can exist on various stoichiometric CeO_2_ surfaces, including the low-index (111) and (100) surfaces and the high-index (221), (223), and (132) ones.^169^ Depending on the coordination numbers of the surface Ce, the stability of Ce–H was different. Specifically, the stability of the hydride species was higher if the coordination number of the surface Ce was lower. In addition to the correlation between the stability of the hydride with the coordination numbers of the surface Ce, it was also proposed that the pairs of hydride/proton species from heterolytic dissociation of H_2_ were thermodynamically stable on the CeO_2_(100) surface.^170^

**Figure 22:**
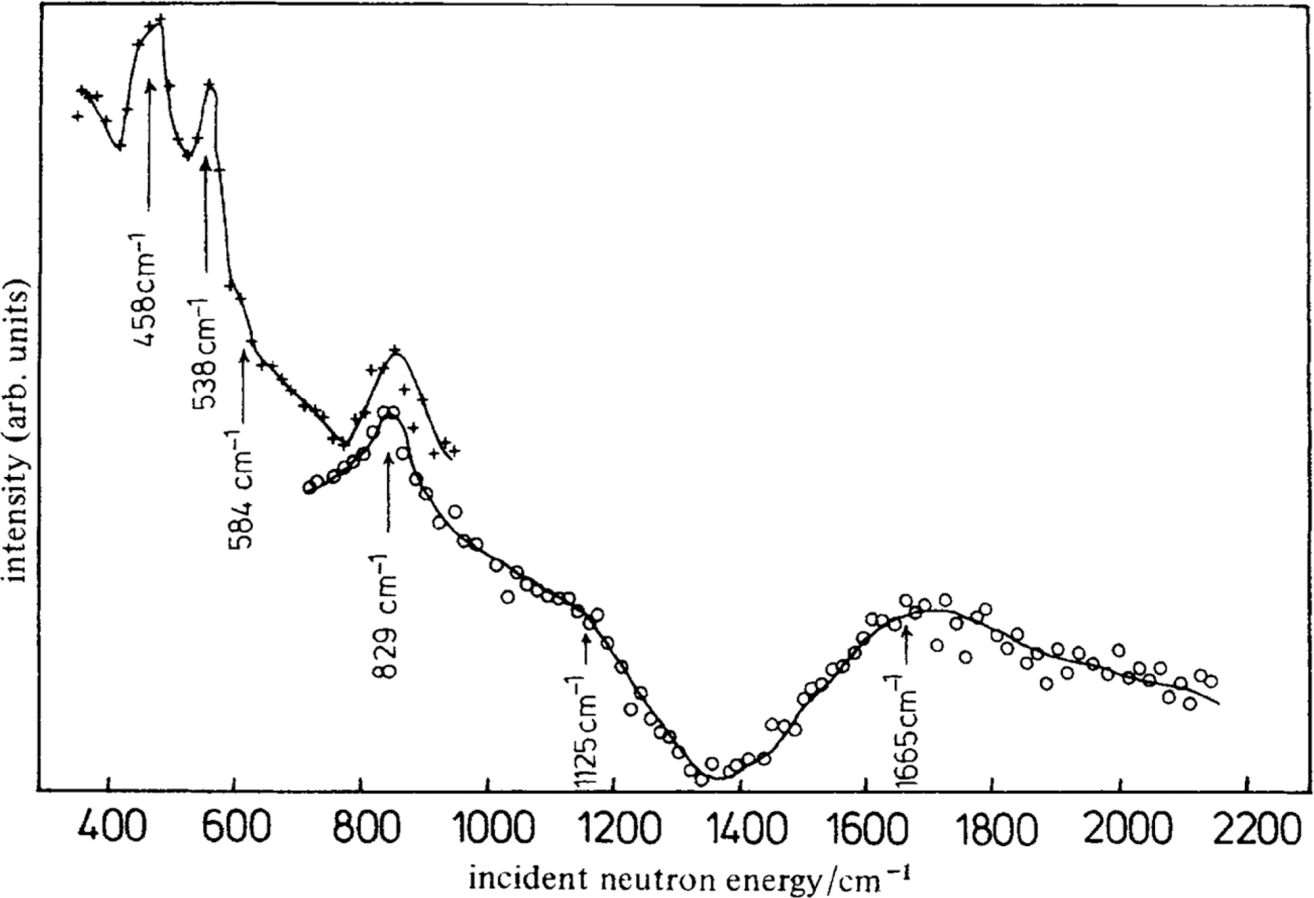
Difference spectrum: INS spectrum of ZnO + H_2_, minus INS spectrum of ZnO. The symbol “+” indicates data collected using the (200) plane, and “o” those collected using the (220) plane of the copper monochromator. Reproduced with permission from ref (172). Copyright 1984 Royal Society of Chemistry.

Neutron spectroscopy has also been applied to study hydride formation over ZnO since it was suggested that O vacancies on ZnO could promote the heterolytic dissociation of H_2_ and the stabilization of hydride species.^171^ As shown in Figure 22, several bands were observed on the INS spectrum of the H_2_–ZnO system. The intense band at 829 cm^–1^ and a strong, broad band at 1665 cm^–1^ were correspondingly assigned to bending and symmetric stretching modes of Zn–H from reversible dissociative adsorption of H_2_ on Zn–O dimers.^172^ The position of the symmetric stretching mode (1665 cm^–1^) was different from that observed by infrared spectroscopy (1710 cm^–1^), as the INS band was too broad to locate the center accurately. DFT calculations indicated that the 1665 cm^–1^ band was contributed by Zn–H species on the nonpolar surfaces and Zn surface.^173^ The 1125 cm^–1^ band was ascribed to the bending mode of −OH. According to the DFT results, the shoulder at 584 cm^–1^ could be attributed to the bending mode of surface bridging Zn–H–Zn species.^173^ However, the bridging structure (Zn–H–Zn) at 1475 cm^–l^ detected by infrared spectroscopy could not be confirmed by INS.^174^ When ZnO was reduced under H_2_ at 20 bar, and at 300 °C, a broadband (∼500 cm^–1^ to ∼1250 cm^–1^) was observed in the INS spectrum.^175^ One might anticipate that hydride species contributed to this band due to the increased amount of oxygen vacancies under a highly reducible environment. However, according to simulated INS spectra, this band was most likely attributed to Zn–OH species (around 750 cm^–1^) rather than bulk Zn–H species (below 500 cm^–1^). Meanwhile, the contribution from surface Zn–H species could not be ruled out, as surface hydride species might have a band with a higher frequency than the bulk hydrides.

##### 3.2.4.2 Hydroxide Species: OH

Via different methods (e.g., hydrogen spillover), oxide hydroxides can be formed by hydrogen insertion into several metal oxides (e.g., WO_3_, ReO_3_, MO_3_, UO_3_, Mo_*x*_W_1–*x*_O_3_, V_9_Mo_6_O_40_, rutile VO_2_).^176−186^ At room temperature, a low amount of H can be inserted into the structure of metal oxide (e.g., H_0.34_UO_3_) without changing the framework integrity of parent oxides.^187^ Higher hydrogen content could result in the formation of amorphous phases (e.g., H_*x*_V_2_O_5_, *x* > 3).^178,188^ By using INS, different types of hydroxide groups (−OH and −OH_2_) can be observed depending on the polymorphs and the extent of hydrogen insertion.^189^

In the INS spectrum of cubic H_0.4_WO_3_, an intense band at 1145–1170 cm^–1^ was observed and assigned to a M–O–H deformation vibration.^190,191^ In addition, the INS spectrum of *h*-H_0.26_WO_3_ showed bands at ∼1613 and ∼484 cm^–1^ attributed to OH_2_ groups. For *h*-H_0.26_ WO_3_, it was also proposed that H^+^ might exist as H_3_O^+^ in the hexagonal tunnels rather than −OH. On the other hand, for H_0.92_Mo_0.44_W_0.56_O_3_ and H_1.02_Mo_0.70_W_0.30_O_3_, it was proposed that H^+^ presented exclusively as −OH, as evidenced by the strong deformation band of M–O–H at 1089 cm^–1^. These outcomes differ from infrared spectroscopy results, which showed the existence of a hydride species (assigned to a trampoline vibration of the hydrogen atom). However, no hydride species at 690 cm^–1^ was detected by INS.^192^

**Figure 23:**
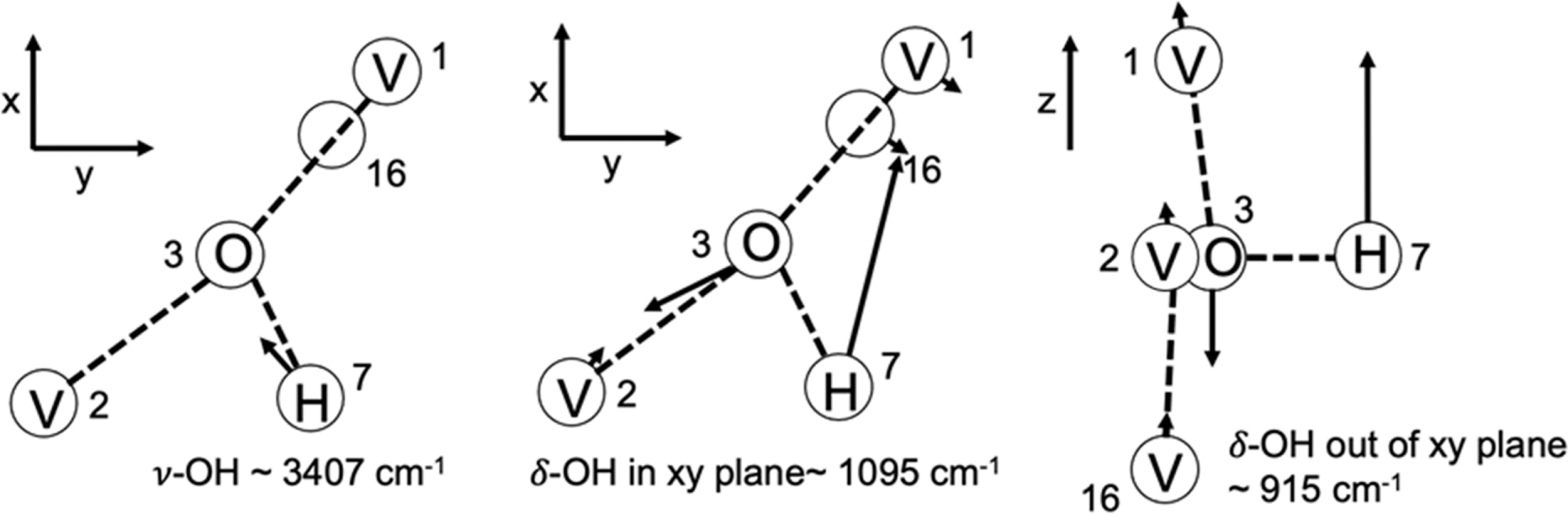
Generated atomic displacements of V_3_OH unit. Reproduced with permission from ref (182). Copyright 1991 Elsevier.

For H_*x*_VO_2_, two intense peaks (1083 and 909 cm^–1^) were observed by INS and ascribed to orthogonal δ-V–OH bending modes according to calculations (Figure 23).^182^ For V_9_Mo_6_O_40,_ which had alternate layers of MoO_3_-like and ReO_3_-like units, the inserted H atoms bonded with the O atoms linking edge-shared octahedral chains and constructing the square windows. INS spectra of H_*x*_V_9_Mo_6_O_40_ (*x* = 7.8 and 17.5) exhibited an intense and broad peak at ∼1081 cm^–1^ assigned to the combination of Mo–OH and V–OH deformation modes.^176^

In the case of MoO_3_, when the H concentration was low (e.g., H_*x*_MoO_3_, *x* = 0.34), −OH groups existed, whereas, at high hydrogen concentrations (e.g., H_*x*_MoO_3_, *x* = 0.93, 1.68, 2.0), the hydrogen atoms tended to form −OH_2_ groups over bridging oxygen atoms of MoO_3_.^191,193−197^ Further reduction of MoO_3_ led to the attachment of H to the terminal O atoms.^187^ The same trend was also observed over H_*x*_UO_3_.^189^ Specifically, in the INS spectrum, the −OH deformation vibration band at 968 cm^–1^ was identified on H_0.35_MoO_3_.^190^ This band shifted to 1267 cm^–1^ in the INS spectrum of H_0.34_MoO_3_.^191^ A band around 1600 cm^–1^ was observed in monoclinic phases of H_*x*_MoO_3_ with higher H contents (e.g., *x* = 0.93, 1.68, 2.0) and was attributed to the H–O–H deformation vibration of the −OH_2_ group. The band at 1200 cm^–1^ was very weak in H_0.93_MoO_3_, indicating that most H existed as −OH_2_ rather than as the −OH group.

#### 3.2.5 Hydrogen on/in Other Catalytic Materials

INS measurements have been applied to study the surface of sulfides, carbides, nitrides, and electrides under H_2_ conditions. Using INS measurements, one could study the H_2_ activation capability of different metals and H-containing species in the system. For instance, metal sulfides (e.g., MoS_2_, CoMoS_2_) are widely applied in the industrial hydrodesulfurization process. In studying MoS_2_, a band at 660 cm^–1^ attributed to the Mo–S–H bending mode was observed, suggesting that H_2_ could be activated and the chemisorbed H species was bounded to the S sites.^198^ However, no bands assigned to H species on Co_9_S_8_ were detected, indicating that Co sites did not function for activating H_2_. For ruthenium sulfide, H–S bands and two different Ru–H linear species (probably related to different Ru facets) were observed.^199^ The hydride species was proposed to be active in hydrogenation since it was weakly adsorbed compared with −SH. Depending on the type of metal sulfides, the position of the −SH bending mode was different: 650 cm^–1^ for MoS_2_, 694 cm^–1^ for WS_2_, 600 and 710 cm^–1^ for RuS_2_.^200^ For a ruthenium sulfide catalyst, it was found that the adsorption of H_2_ depended on the sulfur-to-metal ratio, and coordinatively unsaturated S–S anion pairs were identified as active sites for H_2_ adsorption.^199^

Metal carbides, nitrides, and phosphides are highly active in hydrogenation reactions (e.g., hydrodenitrogenation and hydrodeoxygenation). However, relevant studies about the surface H species over these materials in hydrogenation related reactions are scarce. Based on INS data, it was found that the adsorption site of H depends on the composition of the carbide. For instance, in the H_2_–NbC_*x*_ system, peaks at 774 cm^–1^ for NbC_0.76_H_*x*_ and 524 cm^–1^ for NbC_0.76_D_*x*_ were assigned to optical vibrations of H or D atoms occupying the centers of carbon vacancies.^201^ On the NbC_0.71_H_0.28_ sample, an additional peak at ∼1049 cm^–1^ was observed, which might be related to H atoms at sites displaced from the centers of carbon vacancies. Such sites were relatively unstable, as evidenced by the significantly decreased intensity of the 1049 cm^–1^ peak at elevated temperatures.

**Figure 24:**
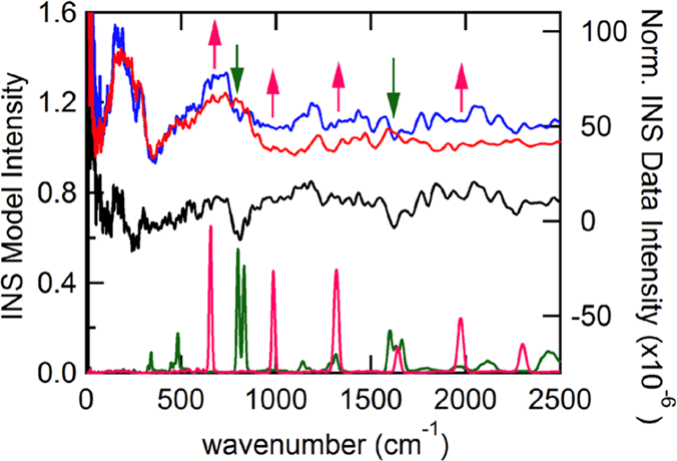
(right axis) Normalized INS spectra for (red) γ-Mo_2_N and (blue) γ-Mo_2_N-H_x_ samples and their difference spectrum (black, γ-Mo_2_N-H_x_ – γ-Mo_2_N). (left axis) Simulated INS spectra (summed over zero to four quantum transitions). Areas in the difference spectrum showing loss upon the addition of hydrogen match the computed κ^1^-NH surf model (green) well. Areas showing growth upon the addition of hydrogen are consistent with a μ^6^-Mo_6_H submodel (pink). Reproduced with permission from ref (202). Copyright 2016 American Chemical Society.

INS could help determine the site for H adsorption in systems with multiple surface sites. For instance, when γ-Mo_2_N was exposed to H_2_ at elevated temperature, γ-Mo_2_N–H_*x*_ (0.061< *x* < 0.082) phases were detected.^202^ According to H_2_-temperature programmed desorption, there were at least two hydrogen binding sites on the surface of γ-Mo_2_N–H_*x*_. DFT results suggested that hydrogen heterolytically dissociated on the γ-Mo_2_N. Surface N (κ^1^-NH_surf_), surface Mo (κ^1^-MoH_surf_), and interstitial Mo (μ^6^-Mo_6_H_sub_) were the sites for adsorbing H. INS was employed to get detailed information on the H adsorbing sites, and several peaks were detected. Peaks at 800 and 832 cm^–1^ were assigned to κ^1^-NH_surf_. Peaks at 658, 986, and 1324 cm^–1^ were attributed to μ^6^-Mo_6_H_sub_ (Figure 24), suggesting that H preferred to adsorb on the interstitial Mo sites over the surface Mo sites. It was also inferred that the subsurface H might migrate to the surface once the reactant (crotonaldehyde) had consumed the surface H species.

By monitoring the surface species, INS can also examine if the moieties are active or spectator species. For instance, although many electrides were successfully synthesized recently and exhibited interesting catalytic properties in certain reactions (e.g., ammonia synthesis), it needed to be clarified whether the hydride species in the bulk were involved in the reactions. INS spectra and DFT calculations were combined to reveal the role of encaged hydride species on Ru/C12A7:e^–^ under ammonia synthesis conditions.^20^ According to the INS spectra, the intensity of the hydride band did not change significantly when the catalyst was exposed to N_2_, suggesting that the hydride species in the cage was chemically stable and might not be the active species in ammonia synthesis. Instead, it was proposed that the surface-adsorbed H species was responsible for the activity of the Ru/C12A7:e^–^ catalysts in NH_3_ synthesis.

#### 3.2.6 Adsorbed Hydrocarbons on Catalyst Surfaces

This section is focused on how neutron scattering techniques can be used to interrogate catalytic systems involving hydrocarbons. Many catalytic systems connected with contemporary chemical manufacturing techniques involve hydrocarbon transformations. For heterogeneously catalyzed processes, turnover cannot occur without the adsorption step. This section reviews the phenomenon of hydrocarbon adsorption, primarily from the perspective of using INS to access the vibrational spectra of adsorbed moieties. It is informative to consider aspects of the early pioneering work in this area and to follow the topic in time when, via a combination of improved spectrometer specifications and sample environment options, increasingly more complex adsorption systems are being investigated.

INS studies of adsorption (physisorption and chemisorption) in the 1970s and 1980s tended to concentrate on olefin adsorption over various substrates. For example, in 1977, Howard and Waddington used the beryllium filter detector (BFD) spectrometer located at the Atomic Energy Research Establishment (AERE) at Harwell (UK) to examine acetylene adsorption on Ag^+^ exchanged 13X zeolite.^203^ The BFD spectrometer provided somewhat limited resolution. The authors deduced from a partial vibrational assignment that acetylene was adsorbed nonlinearly on Ag-13X. Following on from work by Jobic and co-workers on Raney platinum,^204^ Graham and Howard used a combination of spectrometers located at the AERE and the Institute Laue-Langevin (France) to examine the adsorption of benzene on platinum black.^205^ A strong coverage dependence of the spectra was reported, and the authors could deduce that the plane of the molecule was parallel to the metal surface.

In 1985 Kelly and co-workers using a triple-axis spectrometer located at the National Bureau of Standards (USA), measured the INS spectra of ethyne and ethene on Raney nickel.^206^ Decomposition processes were observed on thermal ramping. The vibrational spectra of the molecularly adsorbed species were obtained and compared to vibrational electron energy loss spectra obtained using a nickel single crystal. Contrasts in the spectra enabled the authors to conclude that steps and edge sites on the high surface area material exhibited a reduced activation energy for dissociation.^206^ In 1992, Jacqueline Nicol reviewed the topic of using INS to investigate chemisorbed hydrogenous molecules, which included an informative section on the important matter of sample environment considerations.^207^

**Figure 25:**
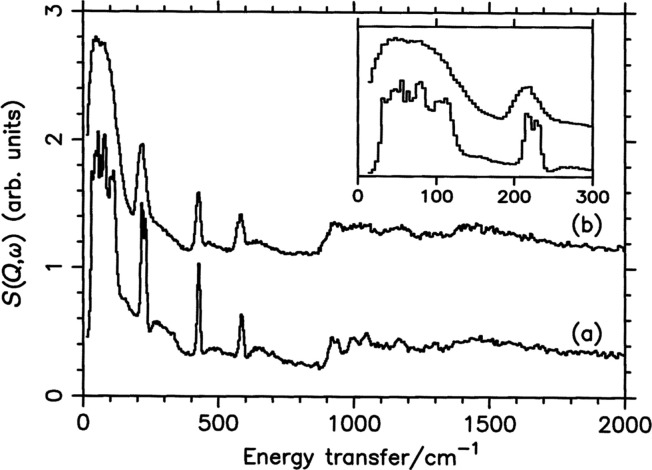
INS spectra at 20 K of (a) propene and (b) propene adsorbed on carbon. Reproduced with permission from ref (209). Copyright 2000 Royal Society of Chemistry.

Improvements in spectrometer design in the 1990s and thereabouts led to improved resolution and sensitivity, enabling a wider range of substrates to be examined. For example, Henson and co-workers used the filter difference spectrometer (FDS) located at the Los Alamos National Laboratory (USA) to investigate the adsorption of ethene on Na zeolite Y.^208^ McNamara and co-workers used the TOSCA spectrometer located at the ISIS Neutron Facility of the Rutherford Appleton Laboratory (UK) to examine the physisorption of ethene and propene on activated carbon that was representative of that used as a catalyst support material for dispersed metal catalysts.^209^Figure 25 shows the spectra of solid propene (a) compared to that of the adsorbed variant (b). Normal coordinate analysis revealed the alkene as a physisorbed, disordered layer on the carbon.^209^

A further example of INS being used to assess hydrocarbon adsorption over catalytically relevant materials is represented by the work of Beta and co-workers who examined the adsorption of furan over alkali metal cation exchanged faujasite.^210^ In this case, the IN1 BeF spectrometer of the ILL was used to acquire the data. Shifts in C–H stretching modes and out-of-plane C–H bending frequencies enabled the authors to address interactions between basic lattice oxygen atoms and the slightly acid C–H bonds of the furan and between the π-electron system of furan and the corresponding cation. Furthermore, the work highlighted a role for adsorption inducing several compensation effects between various furan fundamental modes.^210^

**Figure 26:**
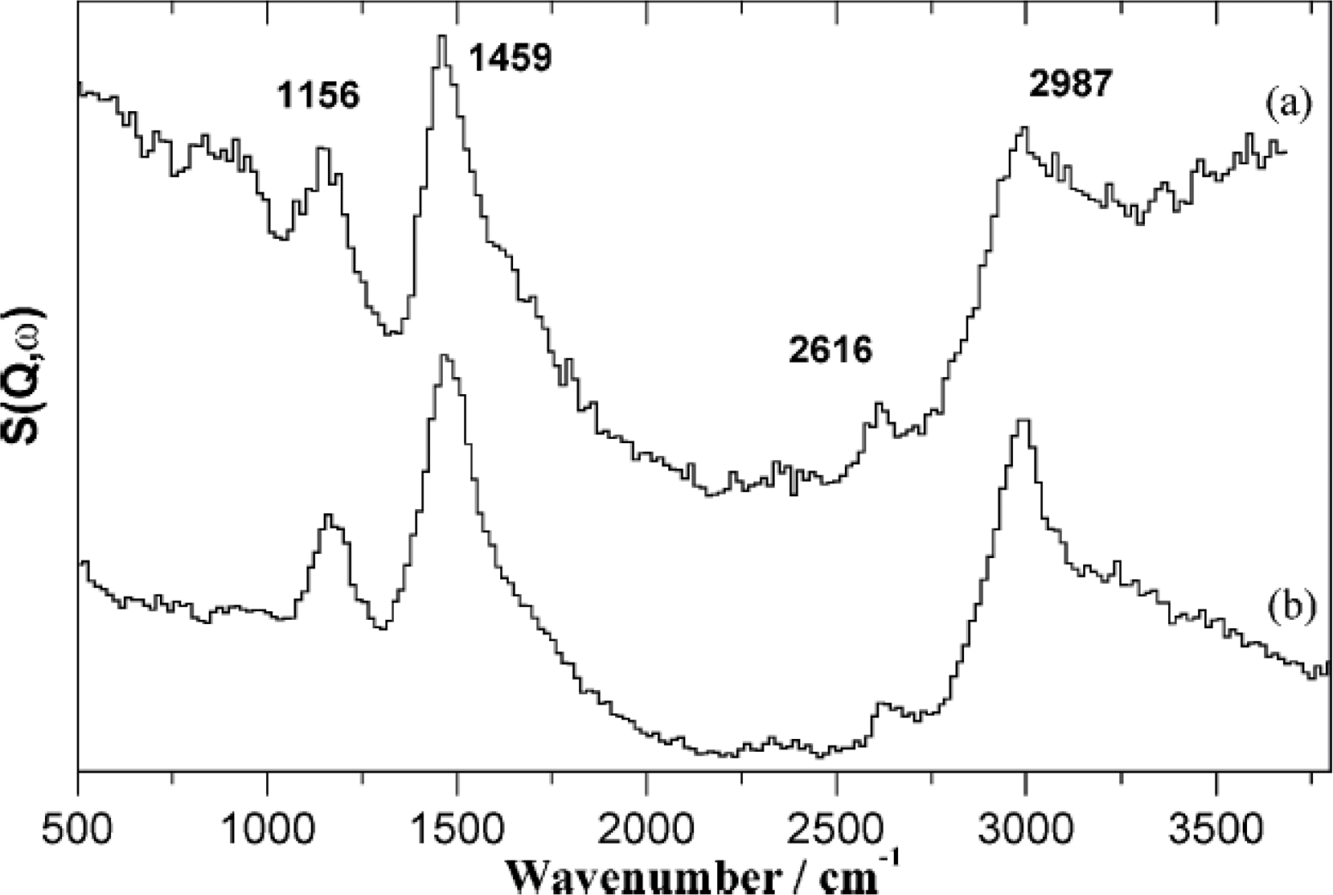
INS spectra of (a) a saturated chemisorbed overlayer of methoxy on η-alumina and (b) Al(OCH_3_)_3_. Reproduced with permission from ref (213). Copyright 2005 Royal Society of Chemistry.

Given the pivotal role of methanol in contemporary and future chemical manufacturing processes, methanol adsorption over various substrates has been an active area of endeavor in recent times. INS’s ability to access a wide spectral range of powdered catalysts, often beyond that accessible via infrared spectroscopy,^211^ provided additional impetus to those studies. Representative examples of substrates examined are zeolite X,^212^ η-alumina^213^ and metal–organic frameworks.^214^ Schenkel and co-workers used the TOSCA spectrometer to investigate the adsorption of methanol on alkali metal cation exchanged zeolite X.^212^ The INS measurements enabled the authors to define the primary interaction of methanol and the zeolite to be the oxygen atom of the alcohol and the cations located at the ion exchange positions of the zeolite. As part of a program of work developing a new generation methyl chloride synthesis catalyst,^215^ McInroy and co-workers used a combination of ISIS spectrometers (TOSCA and MARI) to investigate methanol adsorption over η-alumina.^213^ Whereas TOSCA is an indirect geometry spectrometer with good energy resolution for transitions below ca. 2000 cm^–1^, MARI is a direct geometry instrument that uses a Fermi chopper to monochromate the incident neutron. This latter arrangement allows one to select an incident energy close to the vibrational transitions of interest, offering relatively high resolution over a restricted spectral range. Chemisorbed methoxy groups were identified as the only surface species present under the studied conditions.^213^Figure 26 confirms this deduction, where it compares the INS spectrum of a saturated overlayer of methoxy on η-alumina with that of the model compound Al(OCH_3_)_3_.

**Figure 27:**
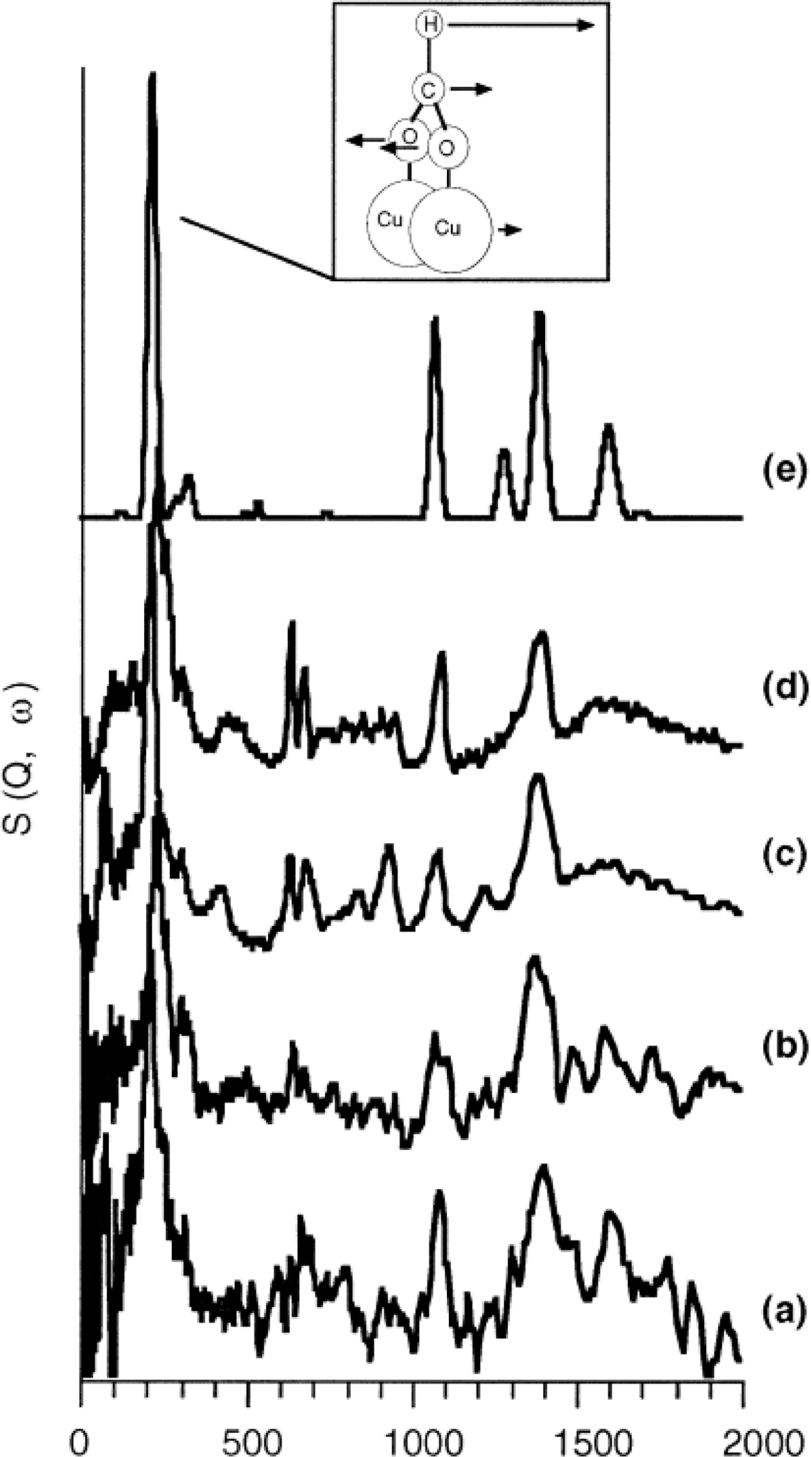
INS spectra of formate adsorbed on (a) Cu_2_O, (b) Cu metal, (c) CuO, (d) bulk copper formate, and (e) DFT simulated spectrum for the adsorption geometry indicated by the inset. Reproduced with permission from ref (216). Copyright 1998 Elsevier.

The industrial production of methanol is typically achieved by applying a copper-containing heterogeneous catalyst of general formula Cu/ZnO/Al_2_O_3_.^150^ To better understand mechanistic options accessible within this reaction system and representing an extension of the methanol adsorption work, several studies have examined the matter of formate adsorption on copper-based materials. For example, Poulston and co-workers have used INS to observe the vibrational spectrum of formate (HCOO) produced by the room-temperature adsorption of formic acid on a range of reduced and oxidized copper surfaces.^216^Figure 27 presents the INS spectra in the 0–2000 cm^–1^ region (TOSCA) for formate adsorbed on Cu_2_O (a), Cu metal (b), and CuO (c). Figure 27d is the spectrum of bulk anhydrous copper formate and Figure 27e is the DFT simulated spectrum for the adsorption geometry indicated by the inset. The most intense features are the out-of-plane and in-plane C–H deformation modes at 1080 and 1389 cm^–1^. The article additionally provided the first report of the C–H torsional mode for the surface species observed at 208–225 cm^–1^.^216^ Subsequent theoretical studies for formate adsorption on copper low index surfaces by Chutia and co-workers corroborate the INS deduced findings.^217^

Recent improvements in spectrometer specification have broadened the range of systems that can be investigated. Interestingly, some of these studies are logical extensions of the work outlined in Nicol’s review.^207^ For example, Duong and co-workers have used the VISION spectrometer operating at the Spallation Neutron Source at the Oak Ridge National Laboratory (ORNL, USA) to identify the optimal binding of ethyne to a nitro-decorated metal–organic framework compound.^218^ Jones and co-workers have used the recently upgraded TOSCA spectrometer^219^ to use ethene adsorption on model skeletal cobalt catalysts to characterize the active site distribution.^220^ Reminiscent of the work of Kelley and co-workers examining adsorption on Raney nickel, the new cobalt studies revealed the presence of highly active sites that induced adsorbate dehydrogenation. In general agreement with a preceding study by Davidson and co-workers on cobalt-based prototype Fischer–Tropsch synthesis catalysts,^119^ as highlighted in Section 3.2.3, the sensitivity of the cobalt catalysts to surface hydroxylation was additionally noted.

In brief, the application of inelastic neutron scattering to investigate hydrocarbon adsorption is an active area of research that provides a baseline for understanding the related but more dynamic process of heterogeneous catalysis facilitating hydrocarbon reforming. Recently realized improvements in spectrometer performance, in terms of sensitivity and resolution, have enabled a wider range of reaction systems to be investigated than was previously the case.

### 3.3 INS Studies of Catalytic Reactions

#### 3.3.1 Hydrogenation/Dehydrogenation Reactions

##### 3.3.1.1 Ammonia Synthesis

**Figure 28:**
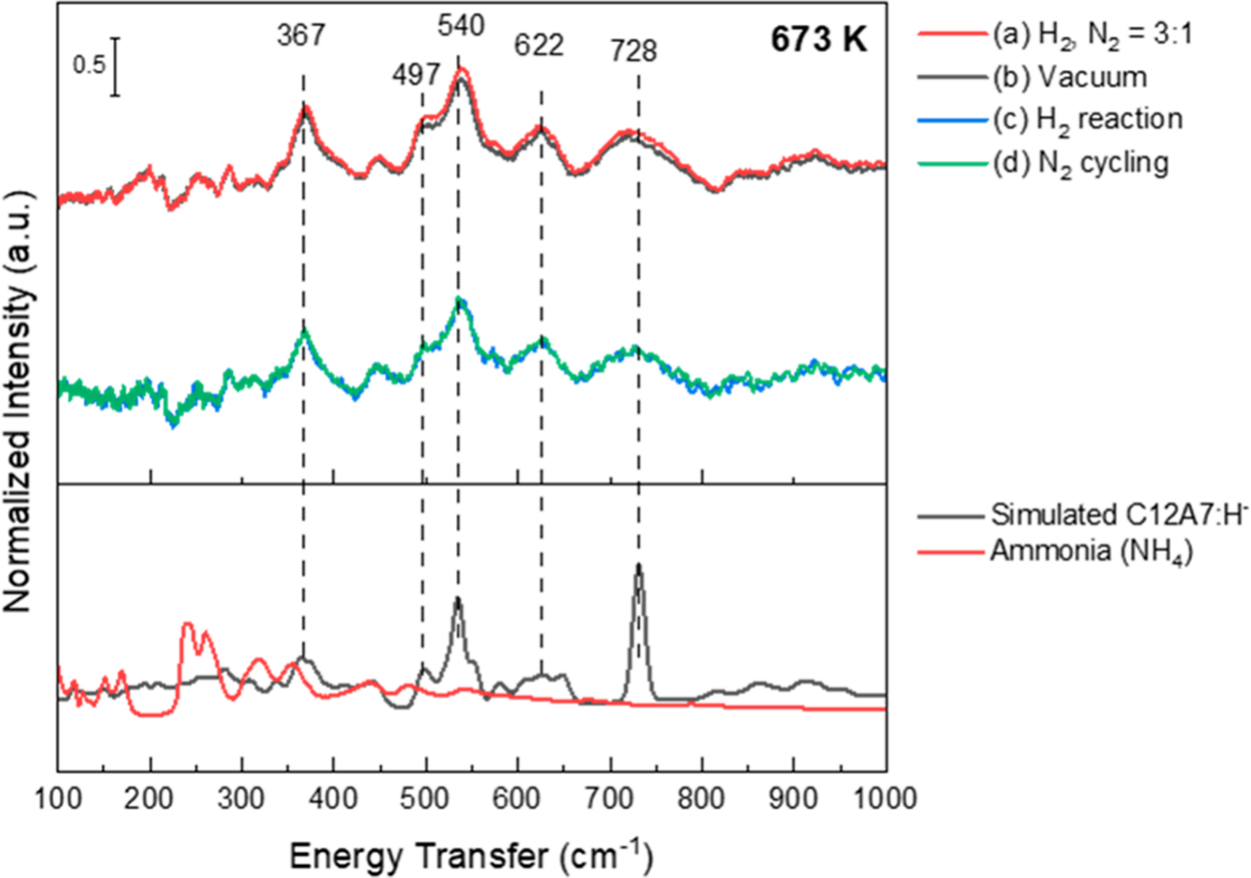
INS spectra of Ru/C12A7:e^–^ collected at 5 K after exposure to (a) 0.1 MPa 3:1 H_2_:N_2_ mixture, (b) vacuum, (c) 0.1 MPa H_2_, and (d) 5 cycles of 0.1 MPa N_2_ at 673 K. Spectra are offset for clarity. Simulated C12A7:H^–^ spectra and measured ammonia spectra are also shown for comparison. Reproduced with permission from ref (20). Copyright 2020 American Chemical Society.

Ammonia is crucial for global food production and can be used as a hydrogen storage chemical.^221,222^ For over a century, ammonia synthesis has been conducted by reacting H_2_ and N_2_ in the Haber–Bosch process.^223^ Fe-based catalysts are typically used for ammonia production; however, their low activity requires high reaction temperatures (673–773 K). At these temperatures, the reaction equilibrium favors the reactants H_2_ and N_2_; therefore, the reaction is carried out under high pressure (15–30 MPa) to obtain economically viable ammonia yields.^20^ Ru catalysts have shown promising reaction rates at milder conditions, but they suffer from H_2_ poisoning since hydrogen adsorption out-competes N_2_ adsorption. When Ru nanoparticles (NPs) are supported on the electride form of a mayenite structure [Ca_24_Al_28_O_64_]^4+^ (4e^–^) (abbreviated C12A7:e^–^) with solvated electrons in the cages, a highly active catalyst for ammonia synthesis is created.^224^ In the work by Kammert et al.,^20^ a combination of kinetic analysis, neutron scattering, and DFT simulations were used to understand the nature of reactive hydrogen during catalytic ammonia synthesis over Ru/C12A7:e^–^ at ambient pressure. After conducting the ammonia synthesis reaction, ND identified the presence of deuterium, ^15^N or oxygen inside the electride cage. However, the neutron scattering length density of these species was very similar; thus, the species were indistinguishable using only ND. INS measurements were able to identify the species inside the cages as hydrides. The spectrum generated after conducting the ammonia synthesis reaction at 673 K (H_2_:N_2_ = 3:1) (Figure 28a) was in excellent agreement with the simulated spectrum of an encaged hydride C12A7:H^–^ via DFT. Hydride species inside the cages were chemically very stable after various treatments (Figure 28b–d), and thus it was proposed that they played a minor role in the ammonia synthesis reaction. More hydride species could be incorporated into the electride cage at 873 K, compared with 673 K, but the hydrides incorporated at 873 K were also less stable at this temperature.

It was reported that Ru/Ca_2_N:e^–^ outperformed Ru/C12A7:e^–^ in ammonia synthesis.^225^ Ca_2_N:e^–^ reacted with adsorbed H and formed Ca_2_NH species. However, it was not clear about the structure of the Ca_2_NH species formed in the reaction. Recent *in situ* neutron scattering results confirmed the formation of H-containing species on the catalysts.^226^ By comparing different models with the experimental spectra, it was speculated that the formed Ca_2_NH-like species had a segregated structure with H/N layers separated by Ca layers, instead of the traditional structure where H and N are intermixed in the anion layers.

**Figure 29:**
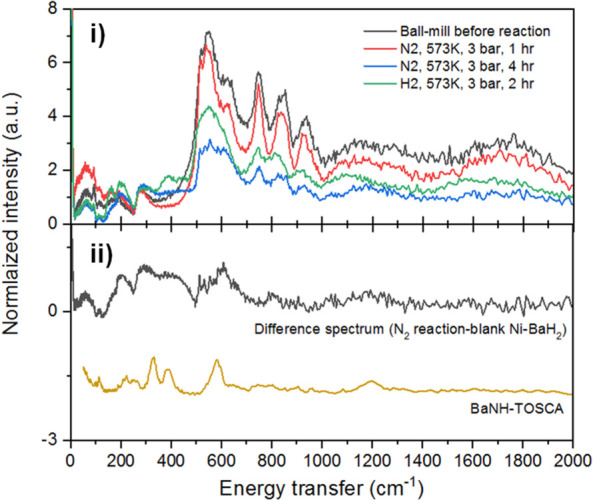
i) INS spectra of Ni/BaH_2_ before reaction, after N_2_ reaction for 1 and 4 h at 573 K, and after H_2_ reaction for 2 h at 573 K. ii) Comparison of difference INS spectrum after N_2_ reaction with that reported for BaNH. Reproduced with permission from ref (101). Copyright 2021 Springer.

Ni/BaH_2_ is a hydride-based catalyst that has been shown to catalyze ammonia production at temperatures below 373 K and ambient pressure via a chemical-looping process. This process consists of two steps: nitrogen fixation and hydrogenation, as shown in Scheme 1.^227^ Moon et al. used INS to evidence the consumption of BaH_2_ during the nitrogen fixation step via the reaction: 2BaH_2_ + N_2_ → 2BaNH + H_2_. Figure 29i shows the INS spectrum of the Ni/BaH_2_ catalyst synthesized via ball-milling, the spectra after N_2_ exposure (for 1 and 4 h), and the spectrum after H_2_ exposure. Reduction of the INS signal intensity during N_2_ treatment reflected the consumption of BaH_2_. The spectra features gained after N_2_ treatment resembled those of BaNH (Figure 29ii). Treatment in H_2_ at 573 K resulted in increased intensity of the INS spectrum due to the incorporation of H_2_ into the catalyst via the following reaction: BaNH + 2H_2_ → BaH_2_ + NH_3_. However, the structure obtained after H_2_ treatment did not quite resemble the initial BaH_2_, suggesting that BaN_1–*x*_H_1+*y*_ (0 < *x*(*y*) < 1) coexisted. Thus, it is suggested that the hydrogenation step is more difficult than the nitridation step.^101^

##### 3.3.1.2 CO_2_ Hydrogenation: Reverse Water–Gas Shift Reaction

**Figure 30:**
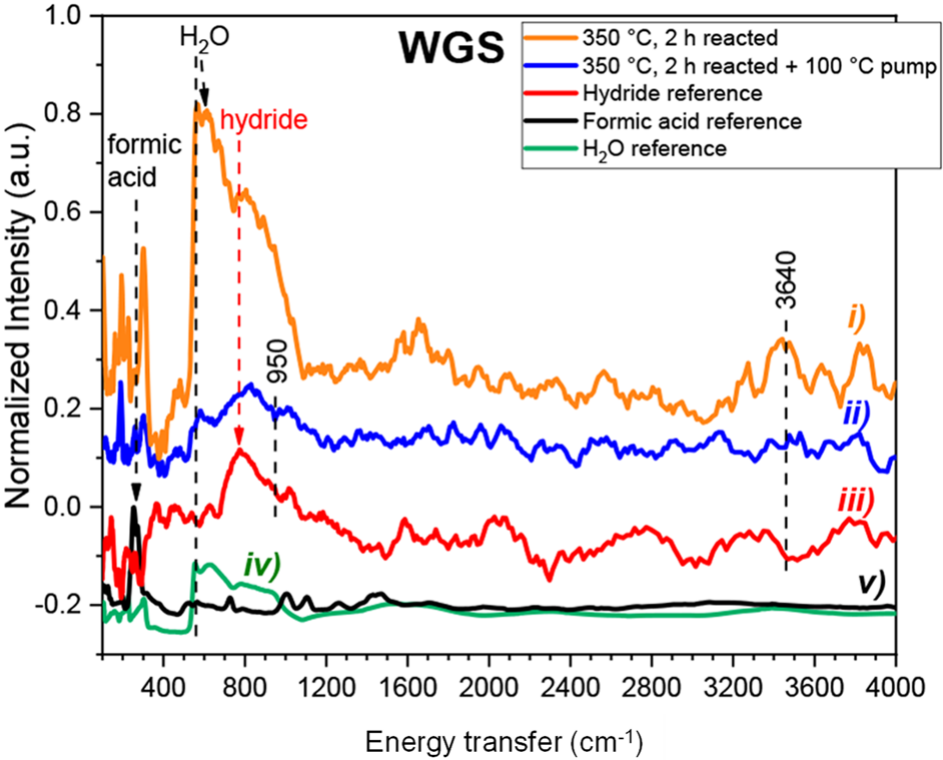
INS spectra i) after WGS reaction (CO/H_2_O = 1) over CuCrFeO_*x*_ at 350 °C for 2 h, ii) after WGS reaction over CuCrFeO_*x*_ at 350 °C for 2 h followed by evacuation at 100 °C. Reference spectra for iii) hydride (on CuCrFeO_*x*_), iv) water, and v) formic acid. Reproduced with permission from ref (230). Copyright 2019 American Chemical Society.

Reduction of atmospheric CO_2_ is of paramount importance to improve the natural balance of our planet. Catalytic hydrogenation of CO_2_ offers an avenue to use this greenhouse gas as a carbon source to produce various chemicals. CO_2_ can be hydrogenated to CH_4_, typically done over Ni catalysts.^228^ INS has assisted in identifying changes in the catalyst structure and surface intermediates during this reaction. During CO_2_ hydrogenation to methane at 300 °C over a commercial Ni-alumina/silica catalyst (reduced in H_2_ at 300 °C before reaction), INS revealed the simultaneous generation of NiO (440 and 580 cm^–1^) and Ni–H species (∼1000 cm^–1^), shining light on key pieces of the reaction mechanism.^229^ Studying the interaction of molecules to produce CO_2_ and H_2_ also provides insights related to CO_2_ hydrogenation. The water–gas shift (WGS) reaction, where CO reacts with water to produce CO_2_ and H_2_, was studied by Polo-Garzon et al. over an industrial-type CuCrFeO_*x*_ catalyst.^230^ They concluded that the “redox” mechanism was predominant. In the redox mechanism, first, CO is oxidized by surface oxygen, and H_2_O subsequently fills the oxygen vacancy to produce H_2_. In the alternative “associative” mechanism, CO and H_2_O come together to form an “associated” intermediate (e.g., formate) on the surface, which later decomposes into CO_2_ and H_2_. INS was used to identify such “associated” intermediate after WGS and reverse-WGS (CO_2_ and H_2_ were reactants) by comparing them with the INS spectrum of formic acid. No evidence of an “associated” intermediate was found. INS spectra also supported the existence of surface Cu–H hydride and hydroxyl species (Figure 30). This evidence, combined with IR spectroscopy, DFT simulations, temperature-programmed surface reaction, and steady-state isotopic transient kinetic analysis (SSITKA), supported the redox mechanism.

##### 3.3.1.3 CO Hydrogenation: Fischer–Tropsch Synthesis (FTS)

Fischer–Tropsch synthesis (FTS) is a well-established catalytic reaction system using synthesis gas (CO and H_2_) obtained from resources such as coal, natural gas, and biomass to produce a variety of hydrocarbon products. Several variants of the process operate around the world, with the catalysts predominantly based on Fe- and Co-containing materials.^231^ Whereas recent large-scale unit operations have tended to feature Co catalysts, there is an increasing interest in Fe-based catalysts, not least as they offer the opportunity to cosynthesize olefins alongside alkanes, which otherwise tend to dominate the product slate.^231^

Despite wide application, there remains considerable uncertainty about how FTS catalysts operate. Schulz describes “the true F-T catalyst to be “constructed” under reaction conditions in processes of self-organization”.^232^ Moreover, when considering possible means for investigating the F-T process, in 2003, Schulz noted the potential of neutron scattering spectroscopy to provide new information on hydrocarbon entities likely to participate in the specific process chemistry.^232^ Whereas analytical techniques such as Mössbauer spectroscopy, X-ray diffraction, and temperature-programmed oxidation applied to Fe-based systems characterize the catalysts in terms of solid-state chemistry (hemeatite, magnetite, iron carbides, etc.)^233,234^ and the presence of carbonaceous materials,^235,236^ INS is one of the few probes that can provide information on how hydrogen is partitioned within the catalyst matrix.^37^

**Figure 31:**
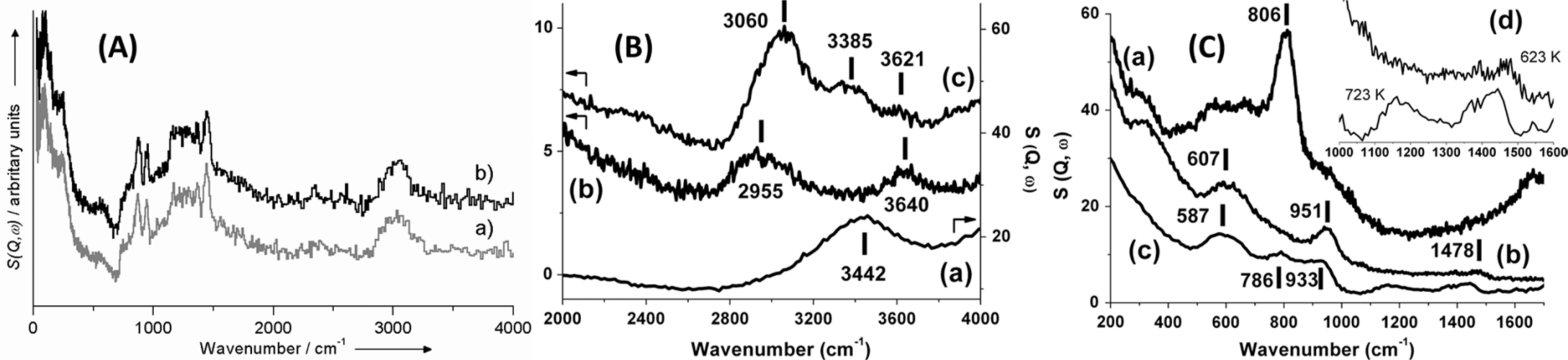
(A) INS spectra of postreaction FTS catalyst samples obtained from the Secunda coal-to-liquid facility that have experienced two solvent extraction regimes: (a) the toluene extracted sample; (b) toluene/CH_2_Cl_2_ extracted sample. Reproduced with permission from ref (237). Copyright 2013 Wiley-VCH. (B) INS spectra of (a) precursor α-Fe_2_O_3_ and post reaction samples after reaction with a CO/H_2_ mixture (1:2) at (b) 623 K and (c) 723 K. Spectra were recorded at an incident energy of 4840 cm^–1^. (C) INS spectra of the (a) precursor α-Fe_2_O_3_ and post reaction samples after reaction with a CO/H_2_ mixture (1:2) at (b) 623 K and (c) 723 K. The inset spectrum (d) is an enlargement of the 623 K (top) and 723 K (bottom) spectra between 1000 and 1600 cm^–1^. Spectra were recorded at an incident energy of 2016 cm^–1^. Reproduced with permission from ref (238). Copyright 2014 Elsevier.

In 2013 Hamilton and co-workers used the technique of inelastic neutron scattering (INS) to interrogate an iron-based technical grade FTS catalyst that had been extracted from Sasol’s Secunda coal-to-liquids facility located in South Africa. Following a Soxhlet extraction procedure to remove the wax and hydrocarbon product from the catalyst, the spectra presented in Figure 31A were obtained on analysis by the TOSCA spectrometer.^237^

The two spectra in Figure 31A are essentially identical, indicating that the spectrum obtained is not dependent on the extraction solvent used. As noted previously (Section 3.1), TOSCA provides good spectral resolution in the 20–2000 cm^–1^ region but experiences resolution broadening at energies >2000 cm^–1^, manifested in Figure 31A by unresolved C–H stretching bands about 3000 cm^–1^. Figure 31A represents the vibrational spectrum of the postreaction catalyst that, due to its deep black coloration, would not have been accessible via optical (e.g., infrared) spectroscopy. Upon analysis, Figure 31A could not be associated with a single molecular entity. Instead, the spectrum was attributed to a mixture of partially saturated polycyclic hydrocarbons^237^ that, overall, signified the presence of a hydrocarbonaceous overlayer of the form described by Webb and co-workers for various metal-based heterogeneous catalytic systems.^239^

To overcome the resolution broadening issue evident in the employment of an indirect geometry spectrometer such as TOSCA (Figure 31A), based on spectrometer configurations considered in Section 3.1, measurements were subsequently undertaken using the direct geometry MAPS spectrometer using distinct incident energies and chopper configurations, as outlined by Parker and co-workers.^31^ These and further measurements on iron-based FTS catalyst formulations utilized laboratory prepared samples, thereby avoiding the challenges and inconvenience of extracting samples from the large-scale chemical manufacturing operation. CO hydrogenation of a syngas mixture at ambient pressure and elevated temperatures to produce methane was selected as a test reaction. This arrangement avoided producing high molecular weight hydrocarbon products favored at elevated pressures, as their formation would significantly complicate the INS spectra but provides information on the critical matter of Fe/CO/H_2_ surface chemistry.^238^Figure 31B,C presents MAPS spectra obtained at respective incident energies of 4840 and 2016 cm^–1^. Figure 31B(a),C(a) shows the spectra of the clean, activated, in-house prepared hematite catalyst (Fe_2_O_3_); Figure 31B/C(b),(c) shows the postreaction spectra of the catalyst after exposure to an ambient pressure syngas mixture at respective reaction temperatures of 623 and 723 K.^238^

Figure 31B provides information on C–H and O–H stretching modes, which are otherwise undiscernible in the TOSCA spectra (Figure 31A). It also shows that the distribution of sp^2^ and sp^3^ hybridized C–H stretching modes exhibits a temperature dependence. The features in the postreaction spectra of Figure 31C are attributable to a combination of C–H deformation modes, hydroxyl deformations, and certain Fe–O transitions. Comparing the low- and high-temperature spectra, consistent with the trends evident in Figure 31B, Figure 31C(b) is largely aliphatic, while Figure 31C(c) is predominantly aromatic.^238^

Subsequent studies of FTS catalysis concentrated on improved catalyst preparation procedures and the development of experimental protocols, not least sample environment options.^240^ These developments enabled Davidson and co-workers to apply the capability of the MAPS spectrometer to explore how the INS spectra of the hydrocarbonaceous overlayer correlated with (i) the reaction profile and (ii) inspection of the catalyst matrix over the reaction coordinate by a variety of analytical techniques, such as X-ray diffraction, Raman scattering, and temperature-programmed oxidation.^241^

**Figure 32:**
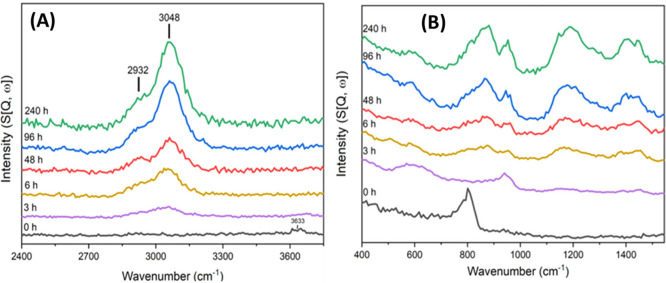
INS spectra for a Fe-based Fischer–Tropsch catalyst after continuous exposure to syngas (CO:H_2_ = 1:2) at 623 K in the large-scale reactor for 0 (black), 3 (purple), 6 (yellow), 48 (red), 96 (blue), and 240 h (green): (a) incident energy = 650 meV (5243 cm^–1^), (b) incident energy = 250 meV (2017 cm^–1^). Reproduced with permission from ref (241). Copyright 2020 American Chemical Society.

Figure 32 presents the high (a) and low (b) energy scans for the hematite catalyst as a function of time-on-stream over a period of 10 days (240 h).^241^ Importantly, the progressive evolution of the hydrocarbonaceous overlayer as a function of time on stream (TOS) was evident in both sets of spectra, with the overlayer form seemingly maturing at approximately 100 h. Ultimately, reflecting aspects of the complexity of iron-based FTS catalysts highlighted by Schultz,^232^ this novel series of measurements revealed different aspects of the complex catalyst evolutionary process to be indirectly connected with catalytic turnover. Further work is needed to better understand the interdependence of the various physical parameters and sustained FTS catalysis. Just recently, those efforts have been extended to include analysis of candidate Fischer–Tropsch to olefins catalysts.^242^

##### 3.3.1.4 Methanol Synthesis

**Figure 33:**
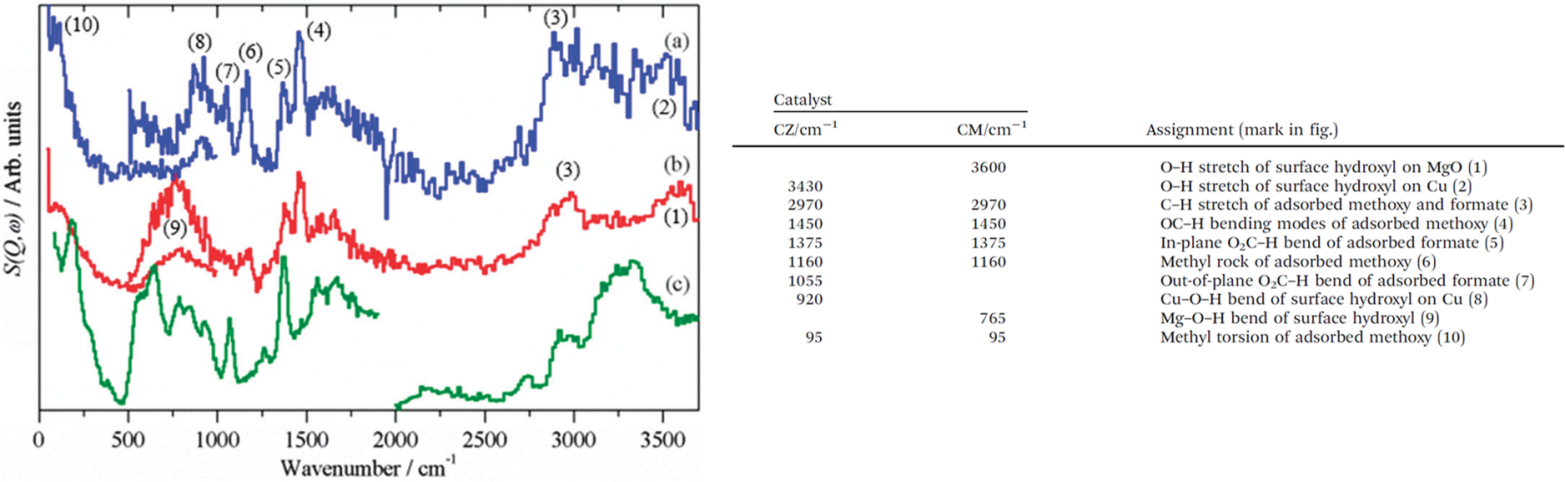
(left) Difference INS spectra after methanol synthesis in CO_2_/H_2_: (a) Cu/ZnO (CZ), (b) Cu/MgO (CM), (c) reference spectrum of Cu(HCOO)_2_·4H_2_O. (right) Peaks assignment. Reproduced with permission from ref (245). Copyright 2016 Royal Society of Chemistry.

Alcohols constitute platform chemicals for several other compounds. A particular commodity alcohol molecule is methanol, from which a variety of chemistry can be derived.^243^ Methanol can be synthesized from CO_2_ or CO hydrogenation. Cu/MgO is considered as a good CO hydrogenation catalyst, whereas Cu/ZnO is preferred for CO_2_ hydrogenation, making it evident that the support has a role in the catalytic cycle.^244^ Kandemir et al. performed kinetic tests combined with INS experiments to elucidate the key reaction intermediates for methanol synthesis using Cu/MgO or Cu/ZnO under two different feed mixtures (CO/H_2_ or CO_2_/H_2_).^245^ During the catalytic reaction using CO/H_2_ as feed, the evolution of the products (CO_2_, H_2_O, CH_3_OH) with time-on-stream was very similar for the two catalysts. INS showed that after the reaction using the CO/H_2_ feed, both catalysts exhibited adsorbed methoxy as a stable intermediate, and Cu/MgO also had surface hydroxyl groups on the support. When the catalysts were tested using CO_2_/H_2_ as feed, Cu/ZnO showed much higher methanol conversion than Cu/MgO, and Cu/MgO instead produced CO through the reverse-WGS reaction. This time, with the CO_2_/H_2_ feed, both spent catalysts had methoxy, formate, and hydroxyl species on the surface, identified via INS experiments. However, for Cu/MgO, hydroxyls were on the support, and for Cu/ZnO, hydroxyls were on Cu (Figure 33). This work provides unique evidence of surface species formation on the catalysts and their adsorption sites, aiding in constructing a complete picture of the reaction mechanism.

To increase the rate of CO_2_ conversion, removing the product methanol as it is produced can be beneficial. This can be achieved by using catalyst supports capable of adsorbing the product to a large extent. In the work by Nikolic et al., INS was used to follow the transient production of methanol, water, and dimethyl ether (DME) over a Cu/zeolite catalyst at 200 and 250 °C and various reaction times, with the catalyst capable of adsorbing reaction species. A constant “water edge” at around 66 meV (532 cm^–1^) in INS spectra, despite changes in the signals for methanol and dimethyl ether, showed that the generation of these products was temporally decoupled from water generation.^246^

##### 3.3.1.5 Hydrogenation of Unsaturated Hydrocarbons

**Figure 34:**
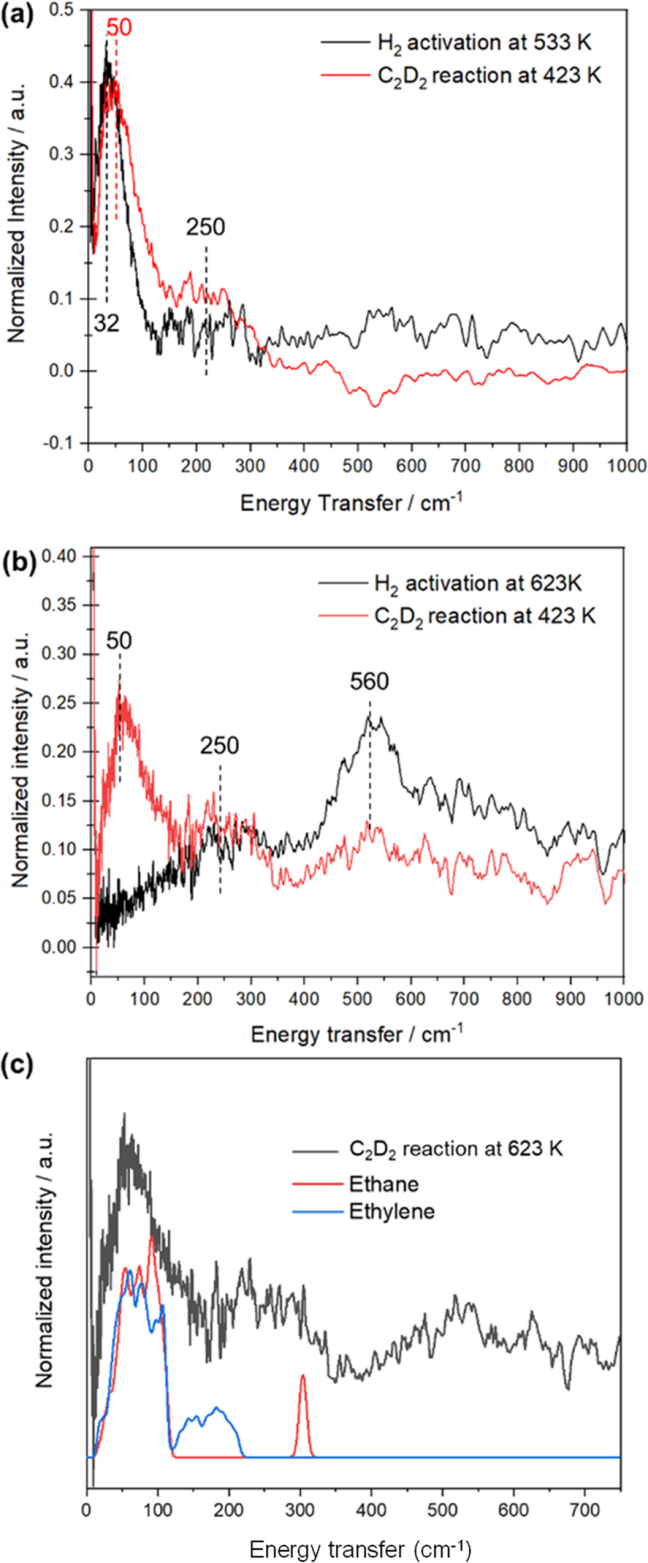
INS spectra of (a) CeO_2_ treated in H_2_ at 533 K and (b) CeO_2_ treated in H_2_ at 623 K before and after reaction with C_2_D_2_. The DFT calculated INS spectra of ethylene and ethane are shown in panel (c). Reproduced with permission from ref (247). Copyright 2020 American Chemical Society.

Hydrogenation of unsaturated hydrocarbons over heterogeneous catalysts is tightly related to the ability of the catalyst surface to dissociate the hydrogen molecule. The mechanism for this dissociation step over metal oxides that do not contain precious metals has been debated for years. INS studies combined with DFT calculations were able to show the existence of surface (>623 K) and bulk (>673 K) cerium hydride after treating CeO_2_ with H_2_, supporting heterolytic dissociation of H_2_ on the metal oxide (producing Ce–H and O–H). However, Ce–H species were only observed after oxygen vacancies are created on reduced CeO_2_.^164^ In a follow-up study, the role of Ce–H and O–H species for acetylene semihydrogenation was discerned by combining INS and infrared spectroscopy.^247^ As shown in Figure 34, surface atomic hydrogen (32 cm^–1^) and hydride species (surface Ce–H, 400–650 cm^–1^) were created on CeO_2_ after H_2_ treatment at 533 and 623 K, respectively. Upon exposure to C_2_D_2_ at 423 K (C_2_D_2_ was used instead of C_2_H_2_ to avoid the signal from hydrogen in C_2_H_2_ dominating the spectra), atomic hydrogen and hydrides were consumed to produce ethylene. Thus, atomic hydrogen and hydrides were shown to be active for acetylene hydrogenation over ceria. Infrared spectroscopy showed that when CeO_2_ was treated in oxygen at 673 K, the bridging OH group was the reactive H species to hydrogenate acetylene.^247^

**Figure 35:**
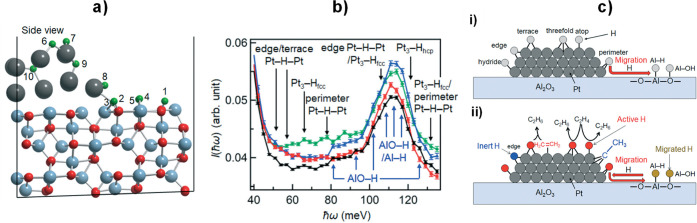
a) Optimized structure of ten H atoms on the supercell of Pt_14_(Al_2_O_3_)_16_ (H: green, Pt: gray, Al: light blue, O: red). The adsorption sites of H atoms are shown as follows: (1) AlO–H, (2) AlO–H, (3) AlO–H, (4) bridge Al–H–Al, (5) bridge Al–H–Al, (6) terrace Pt–H–Pt, (7) edge Pt–H–Pt, (8) perimeter Pt–H–Pt, (9) 3-fold Pt_3_–H_fcc_, and (10) 3-fold Pt_3_–H_hcp_. b) Assignment of the experimental INS spectra. (green) 5 wt % Pt/Al_2_O_3_ with 5% H_2_ (He balance), (blue) 5 wt % Pt/Al_2_O_3_ with 5% C_2_H_4_ (He balance), (red) 5 wt % Pt/Al_2_O_3_ with 5% C_2_H_4_ and 5% H_2_ (He balance), and (black) 5 wt % Pt/Al_2_O_3_ with He. c-i) Activated H species on Pt/Al_2_O_3_ in the presence of H_2_. c-ii) Reaction mechanism of active H species on Pt/Al_2_O_3_ in C_2_H_4_ hydrogenation. Reproduced with permission from ref (248). Copyright 2021 Royal Society of Chemistry.

When supported metal catalysts are used for the hydrogenation of unsaturated hydrocarbons, hydrogen species can be present both on the supported NP and the support, adopting various adsorption configurations and reactivities. Yamazoe et al. studied the hydrogenation of ethylene over Pt/Al_2_O_3_ using INS, IR spectroscopy, and DFT simulations.^248^ An INS spectrum was simulated using a DFT model containing 10 different adsorption sites for atomic hydrogen (Figure 35a). It was found that hydrogen adsorbed on Pt sites and on the Al_2_O_3_ support as AlO–H and Al–H–Al. Adsorption of hydrogen on the support was enabled by H spillover from Pt NPs. Further, it was shown that hydrogen species, generated when ethylidyne species were created, (CH_3_C–Pt_3_) were stored on both Pt and Al_2_O_3_ surfaces. When an H-containing surface was exposed to C_2_H_4_, INS (Figure 35b) and infrared (not shown) signals were reduced, showing that perimeter Pt–H–Pt, terrace Pt–H–Pt, Pt_3_–H, hydride Pt–H, and atop Pt–H were active species for C_2_H_4_ hydrogenation. In contrast, edge Pt–H–Pt was not (Figure 35b). Hydrogen adsorbed on the support (AlO–H and Al–H–Al) did not react with pure C_2_H_4_ directly, but the intensity of their signal reduced in the presence of C_2_H_4_ and H_2_; thus, it was hypothesized that some hydrogen adsorbed on the support could migrate back to Pt sites under reaction conditions (Figure 35c).^248^

#### 3.3.2 Hydrocarbon Conversion, Including Coking

Hydrocarbon conversion over heterogeneous catalysts represents a broad area of activity within the chemical manufacturing sector, encompassing activities as diverse as petroleum reforming, synthesis gas generation, and fine chemicals production.^249^ Given that all these operations involve significant quantities of hydrogen in the transformation process, they are all ideally set up for investigation by neutron scattering techniques. However, despite the apparent economic relevance of these numerous process operations, the number of studies in recent years has been surprisingly modest. Admittedly it is now slowly ramping up, with INS featuring predominantly.^37^ Possibly the most significant impediment to the implementation of neutron techniques in hydrocarbon conversions to date has been the matter of defining the right sample environment conditions to employ that enable a previously working catalyst (high temperature and/or high pressure operation) to be suitably examined by a neutron spectrometer (INS acquisition temperatures of ≤20 K). Following sample environment developments outlined by Nichol for hydrocarbon adsorption over skeletal metals,^207^ Goodman and co-workers proposed an *ex situ* approach to INS analysis of heterogeneously catalyzed reaction systems that essentially involved “quenching” reaction before spectral acquisition.^250,251^ Further sample environment refinements have subsequently elaborated on the *ex situ* approach.^240,252^

A further matter contributing to the complexity of analyzing hydrocarbon conversion reactions is the not insignificant matter of catalyst deactivation. This phenomenon can take different forms (e.g., sintering, poisoning, disruption of active sites, etc.); however, a significant feature is carbon laydown, aka “coking”. Coke formation can take various strands, with the concepts of “hard” and “soft” coke in common use.^249^ Whereas significant coke deposition can seriously compromise catalytic performance (e.g., via pore blocking), the ability to access the vibrational spectrum of the black and partially deactivated catalyst, which would otherwise be inaccessible via conventional optical techniques (e.g., infrared spectroscopy),^211^ represents a distinct advantage for INS.^253^ Thus, in addition to providing information on the fate of hydrogenous moieties active within heterogeneously catalyzed reaction systems, it is possible for INS to contribute in terms of catalyst deactivation pathways involving “coke”. Given the ubiquitous nature of coking in applied heterogeneous catalysis, this is no small matter. INS studies will be discussed below on methane dry reforming and methanol conversion, along with other reactions.

##### 3.3.2.1 Methane Dry Reforming

This subsection will concentrate on INS investigations of a single reaction system as an exemplar of how a neutron spectroscopic technique (INS) can be used to interrogate an industrially relevant hydrocarbon conversion. Specifically, methane reforming over supported nickel catalysts to produce syngas (CO + H_2_) is an important primary reaction for the chemical manufacturing sector,^254^ which McFarlane and co-workers have recently examined. While most of their endeavors concentrated on the so-called dry reforming reaction (which uses CO_2_ as the oxidant),^255−258^ they also considered the matter of the steam reforming variant^259^ that was most typically employed in large-scale industrial operations. This section will concentrate solely on the dry reforming reaction over a specific alumina-supported nickel catalyst.^258^

The production of syngas via the dry reforming of methane represents an alternative to the energy-intensive steam reforming variant that uses carbon dioxide as the oxidant (CH_4_ + CO_2_ → 2CO + 2H_2_). The dry reforming reaction has strong environmental credentials and yields a product mixture suited to upstream processes, such as the Fischer–Tropsch synthesis of relatively high molecular weight hydrocarbons.^260^ Supported nickel catalysts are often utilized for steam and dry methane reforming reactions, with the latter reaction, in particular, being plagued by rapid deactivation issues compared to noble metals (e.g., Pt, Pd), as characterized by excessive carbon retention by the catalyst.^261^ Against this background, it is desirable to secure a better understanding of the processes that lead to diminished activity with increasing time-on-stream for representative dry reforming catalysts. An important parameter in understanding how the catalysts operate is determining how hydrogen is partitioned within the carbonaceous matrix that ultimately impedes conversion. Consequently, INS was used to investigate the degree of hydrogen retained by the catalyst after “quenching” the reaction after a fixed duration of time-on-stream.^258^

**Figure 36:**
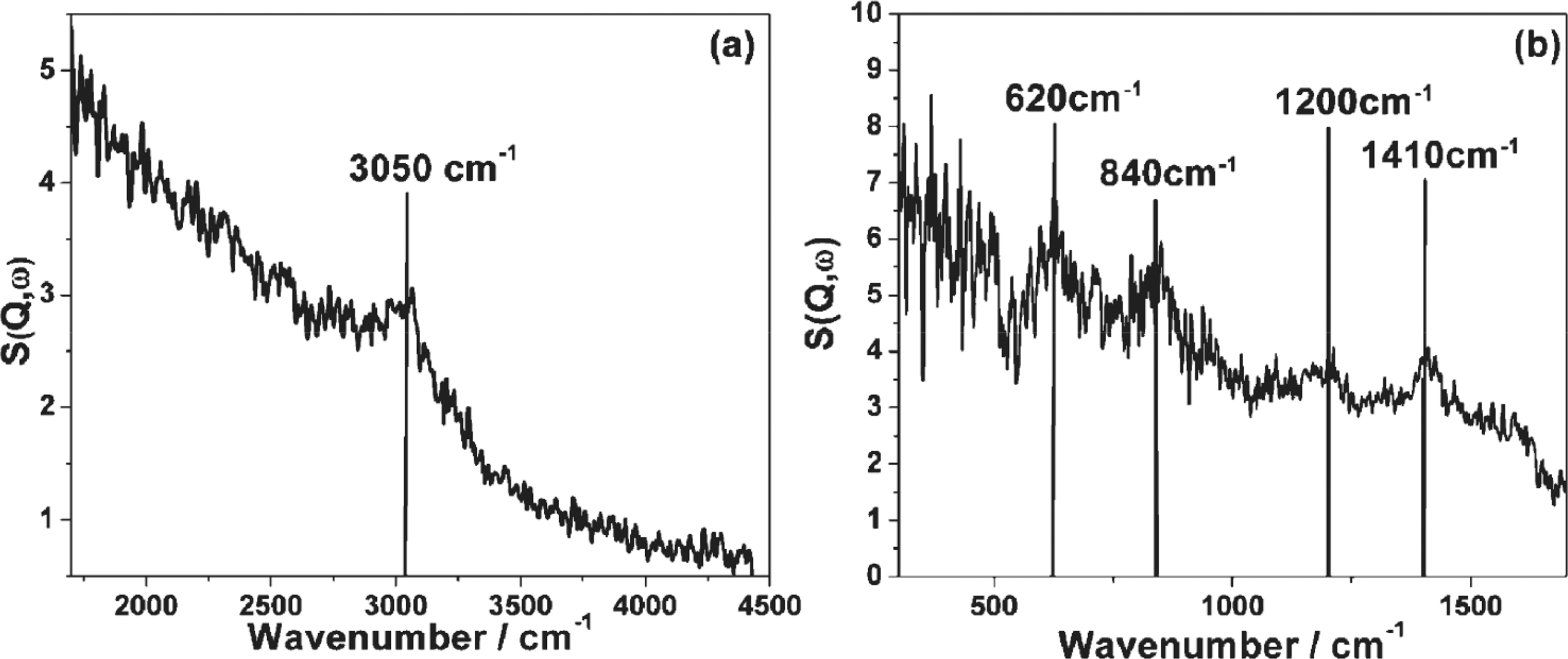
INS spectra of the Ni/Al_2_O_3_ catalyst after 6 h reaction at 898 K. The spectra are difference spectra acquired using the MAPS spectrometer operating at an incident neutron energy of (a) 4840 cm^–1^ and (b) 2017 cm^–1^. Reproduced with permission from ref (258). Copyright 2013 Royal Society of Chemistry.

Figure 36 presents the INS difference spectra of a 26 wt % Ni/Al_2_O_3_ catalyst after a 6 h reaction of methane dry reforming at 898 K, where the spectrum of the clean/activated catalyst has been subtracted from the spectrum of the reacted catalyst. Thus, Figure 36 represents the vibrational spectrum of material retained at the catalyst surface. Spectral acquisition at primary energies of (a) 4840 and (b) 2017 cm^–1^ provides access to the C–H/O–H stretch region and the fingerprint regions of the spectrum, respectively. Figure 36a is characterized by a single weak feature centered at 3050 cm^–1^, which is assigned as an sp^2^ carbon C–H stretch mode. This feature’s poor signal: noise ratio indicates minimal hydrogen retention by the catalyst. Calibration of the C–H stretching intensity using a previously described method^256^ determines the number of hydrogen atoms associated with carbon atoms to be 17.4 ± 1.0 μmol H g^–1^_(cat)_. The absence of any ν(O–H) features in Figure 36a is additionally noted and is attributed to the catalyst preparative procedure adopted.^258^

The spectrum of the reacted catalyst recorded at an incident energy of 2017 cm^–1^ (Figure 36b) is characterized by four discernible bands observed at 1410, ca. 1200, 840, and 620 cm^–1^. Again, the poor signal:noise ratio indicates a low concentration of hydrogen within the carbonaceous matrix. The 1410 cm^–1^ band is close in energy to a CH_2_ scissors vibration. However, there is little evidence for saturated carbon species from the ν(C–H) region (Figure 36a). Instead, this mode is assigned to a coupled aromatic C–C stretch and C–H bend. The broad band centered at 1200 cm^–1^ is assigned to an aromatic in-plane C–H bending mode. The band at 840 cm^–1^ is assigned to an aromatic out-of-plane C–H bending mode, consistent with the ν(C–H) mode observed at 3050 cm^–1^ (Figure 36a), although there could additionally be a contribution from a C–C stretch at 875 cm^–1^. The 620 cm^–1^ band is attributed to the sp^2^ carbon network deformation mode. Collectively, the spectra in Figure 36 indicate the presence of extensive polycyclic aromatic domains that are largely graphitic in nature.

**Figure 37:**
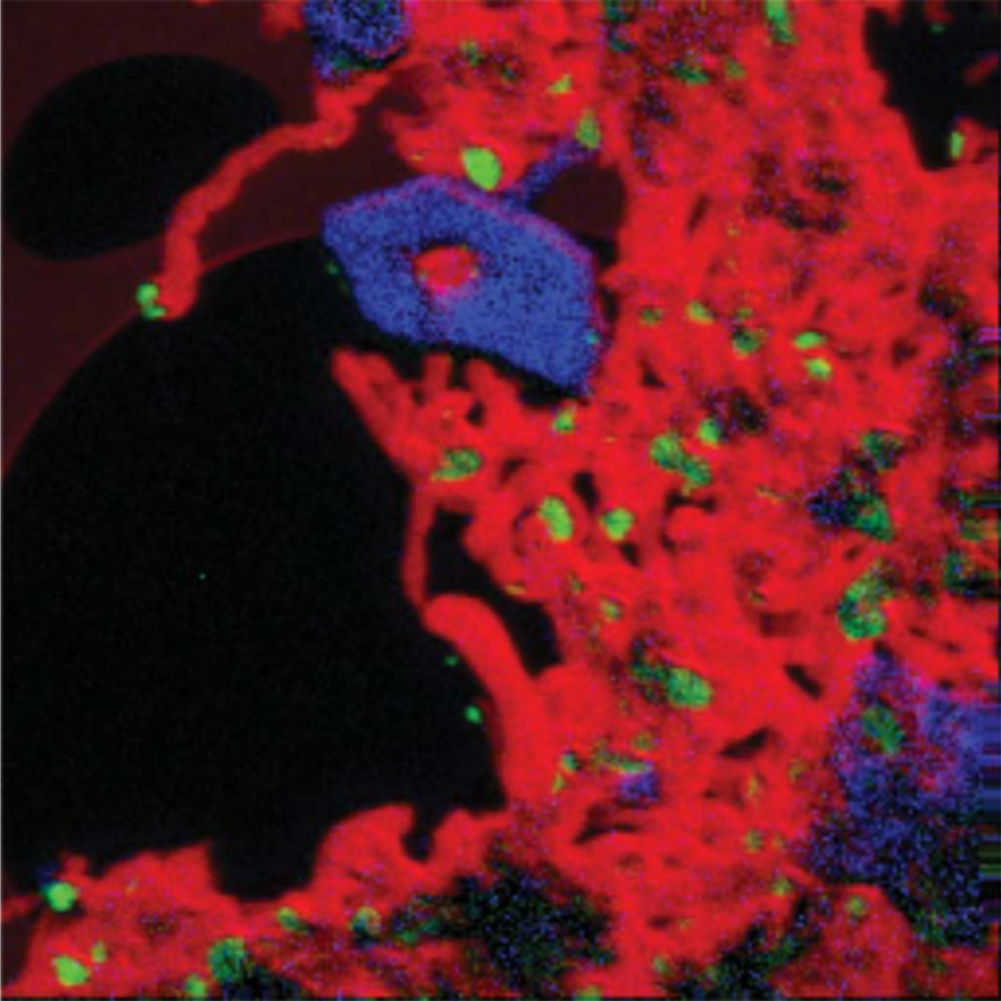
Color-coded energy map transmission electron micrograph of the Ni/Al_2_O_3_ catalyst recorded after 6 h of reaction at 898 K and subsequent INS measurement: red = carbon, green = nickel, and blue = oxygen. Reproduced with permission from ref (258). Copyright 2013 Royal Society of Chemistry.

Figure 37 shows a post-reaction color-coded transmission electron micrograph energy map for the Ni/Al_2_O_3_ catalyst. Dispersed nickel particles (diameter ∼36 nm) are indicated in green. The alumina support material is indicated by the oxygen signal that is colored blue. However, the standout feature of Figure 37 is the extensive red zone that represents the carbon signal. Figure 37 indicates an extensive degree of carbon laydown, which corresponds to a concentration of 44.3 ± 3.5 mmol C g^–1^_(cat)_ according to temperature-programmed oxidation measurements.^258^ The filamentous nature of a nickel-catalyzed carbon deposition pathway is evidenced by the “hook” like structure in the top left-hand corner of Figure 37.

Critical to this multi-technique investigation, the INS measurements unambiguously establish that a sample able to sustain syngas production at elevated temperatures retains minimal quantities of hydrogen (17.4 ± 1.0 μmol H g^–1^_(cat)_), in contrast to a significantly large degree of carbon laydown (44.3 ± 3.5 mmol C g^–1^_(cat)_); corresponding to a C: H ratio of 2546:1. The INS spectra are interpreted as representing the vibrational fingerprint for a small hydrogen population that is decorating terminations of an extensive carbonaceous matrix.^258^ This outcome has mechanistic significance.

##### 3.3.2.2 Methanol to Hydrocarbons

Methanol can be converted to hydrocarbons using zeolite catalysts.^211,262^ Although this technology has been industrially implemented, a complete understanding of the catalyst operation is yet to be attained.^262^ INS is particularly useful for studying the surface species on deactivated catalysts. For instance, room temperature dissociation of methanol on H-ZSM-5 zeolites was observed from INS studies.^263^ DME was an intermediate product in the methanol-to-hydrocarbon (MTH) process. INS studies showed that the “hydrocarbon pool” during DME conversion over ZSM-5 was similar between the deactivated sample (2–3 days of reaction) and the initial catalyst (1 day of reaction). However, the deactivated samples contained more hydrocarbon content. Further, a comparison of surface species with durene and *o*-xylene spectra showed that the aromatic species in the used catalysts were better resembled by *o*-xylene, suggesting that the aromatic species on the spent catalyst were not highly methylated. The ZSM-5 catalyst deactivated quicker with DME as a reactant than with methanol. Quicker deactivation with DME was attributed to a lower water concentration, reducing the regeneration of acid sites.^211,264^

The synthesis of propylene from methanol has been intensively studied due to the growing demand of propylene.^265,266^ In a recent study of this conversion, INS spectra showed that the adsorption of methanol on TaAlS-1 induced the significant broadening of C–O–H deformation (697, 778 cm^–1^) and the O–H stretch (3241 cm^–1^) modes of Me–OH groups while features of – CH_3_ group remained largely the same.^267^ The results suggested that methanol adsorbed on TaAlS-1 via interaction between OH and Ta(V) and Brønsted acid sites. When the reaction was carried out at 350 °C, the presence of the C–O–C scissoring mode at 422 cm^–1^ and the methyl torsions mode at 194 and 245 cm^–1^ confirmed the formation of dimethyl ether (DME) on the catalyst. At 370 °C, the existence of trimethyloxonium (TMO, umbrella mode at 365 cm^–1^) and the librational modes of water at 500–700 cm^–1^ demonstrated that TMO was formed by reaction between DME and methanol. Moreover, the features of propylene were observed, such as methyl torsion (221 cm^–1^), C=C–C scissoring (429 cm^–1^), C=CH_2_ rocking (916 cm^–1^), and C=C stretching (1644 cm^–1^). Based on the INS results, a catalytic cycle was established for propylene synthesis from methanol over TaAlS-1 catalysts with trimethyloxonium as the key intermediate being observed for the first time.

##### 3.3.2.3 Other Hydrocarbon and Hydrogenation Reactions

**Figure 38:**
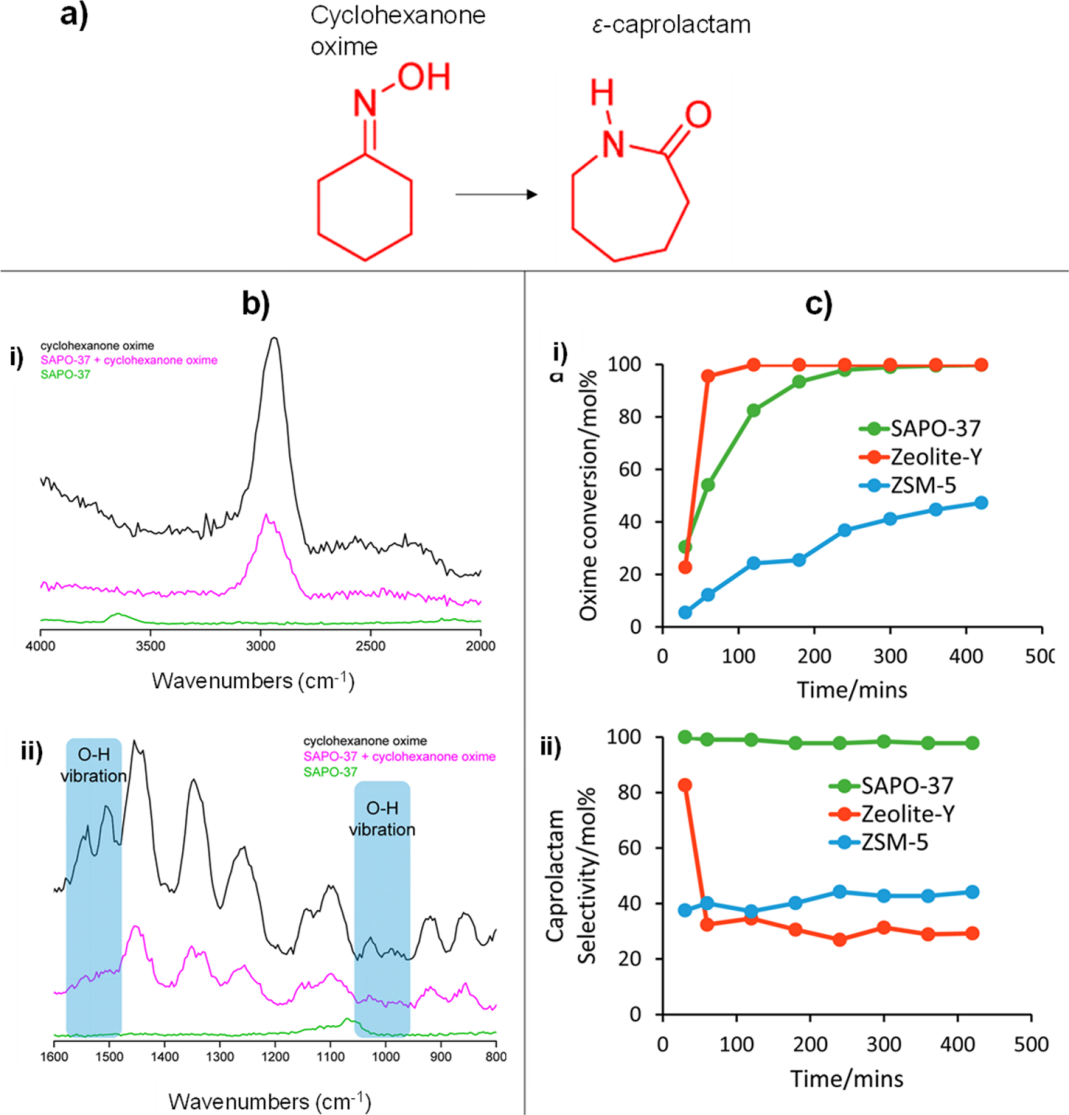
a) Chemical structure of cyclohexanone oxime and ε-caprolactam. b) INS vibrational spectra showing the influence of cyclohexanone oxime binding to SAPO-37. c) Catalytic results of liquid phase Beckmann rearrangement of cyclohexanone oxime over zeolites. Reproduced with permission from ref (268). Copyright 2017 American Chemical Society.

The conversion of cyclohexanone oxime into ε-caprolactam (Figure 38a) via the Beckmann rearrangement is of great interest to the chemical industry, as ε-caprolactam is necessary for the production of Nylon-6. Optimizing zeolite catalysts to perform this reaction requires understanding the dynamic diffusion of cyclohexanone oxime through the zeolite pores and its interaction with the surface sites. The work by Potter et al.^268^ using QENS showed Fickian diffusion of cyclohexanone oxime through the pores of Zeolite-Y, moving freely straight through the pore, whereas the reactant moved in a jump-like manner through the pores of SAPO-37. In contrast, cyclohexanone oxime did not access the internal sites of ZSM-5. INS experiments were performed to better understand this diffusional behavior. When SAPO-37 interacted with the oxime, the intensity corresponding to the O–H stretch of the framework hydroxyls (3650 cm^–1^) was reduced (Figure 38b-i). When comparing the pure oxime with its adsorbed form, intensities corresponding to the O–H vibration of the oxime were reduced when it adsorbed on the surface (Figure 38b-ii). This supported that −OH functionality of the oxime interacted with the acid sites of SAPO-37, which led to a jump-like diffusion through the pores. In catalytic tests, Zeolite-Y and SAPO-37 showed the highest conversions (versus ZSM-5) due to the accessibility to the pores. SAPO-37 showed the highest selectivity (Figure 38c), indicating the importance of the interaction of the reactant with the active sites inside the pores.

The hydrodeoxygenation of biomass-derived oxygenates to chemicals and fuels is important to global carbon neutralization.^269−271^ Metal catalysts are widely applied for this reaction, and the adsorption mode of oxygenates on the catalysts is crucial to the products distribution and understandings in reaction mechanisms.^272,273^ Neutron scattering techniques are well positioned to study the conversion of biomass-based oxygenates as many functional groups of the oxygenates contain H.

In the hydrodeoxygenation of phenol over Ru/Nb_2_O_5_, INS revealed the formation of hydrogen-containing species (Nb–OH and/or Ru–H) after reduction at 150 °C.^274^ Compared with the spectrum of condensed phenol, the spectrum of phenol adsorbed over catalyst surface showed that the strong adsorption restricted the motion of phenol (C_6_-ring). The librational modes of phenol (155–240 cm^–1^), C_6_-ring deformation, and −CH– out-of-plane wagging mode disappeared upon adsorption. Besides, the adsorption caused the deprotonation of the phenol, evidenced by the significant decrease in the intensity of the −OH bending mode at 948 cm^–1^. A similar change in the spectrum upon the adsorption of phenol was observed over Nb_2_O_5_ support as well. At the beginning of the reaction between the adsorbed phenol and H_2_, INS detected the formation of cyclohexanol (C_6_-ring deformation mode at 236 cm^–1^ and ring-conformational modes at 341 cm^–1^) and benzene (398 and 606 cm^–1^). It proved the competition between ring hydrogenation and cleavage of the C–O bond. Adsorption of the phenol on Ru/ZrO_2_, Ru/Al_2_O_3_, and Ru/TiO_2_ catalysts showed similar but weaker features compared to the Ru/Nb_2_O_5_ catalyst.

The INS spectra of tetrahydrofuran (THF) adsorbed on Pt/Nb_2_O_5_ indicated that the adsorption hindered the motion of the intact THF structure and proceeded via O(δ^–^) on the Nb(δ^+^) site.^275^ At the first 10 min in H_2_ at 130 °C, the disappearance of −CH_2_– twisting and internal ring deformation modes of THF at 1,244 and 1,308 cm^–1^ suggested the cleavage of the THF ring. Moreover, INS detected methyl torsion (245 cm^–1^), −CH_2_CH_2_– rocking (744 cm^–1^), −CH_2_CH_2_CH_3_ rocking (805 cm^–1^), and C_4_ chain (477 cm^–1^) of 1-butanoxide on the catalyst. According to the INS spectra, it was proposed that the conversion of THF proceeded through adsorption, binding, ring-opening, partial hydrogenation, and complete hydrodeoxygenation to produce butane.

#### 3.3.3 INS Investigations of Oxidation Reactions

Oxidation or redox reactions are critical chemical transformations in making value chemicals, environment remediation, etc. Neutron scattering studies of redox reactions are relatively scarce unless hydrogen-containing species are involved. Two examples involving INS work are presented below on H_2_ oxidation and CO oxidation.

##### 3.3.3.1 H_2_ Oxidation: H_2_O_2_ Synthesis

**Figure 39:**
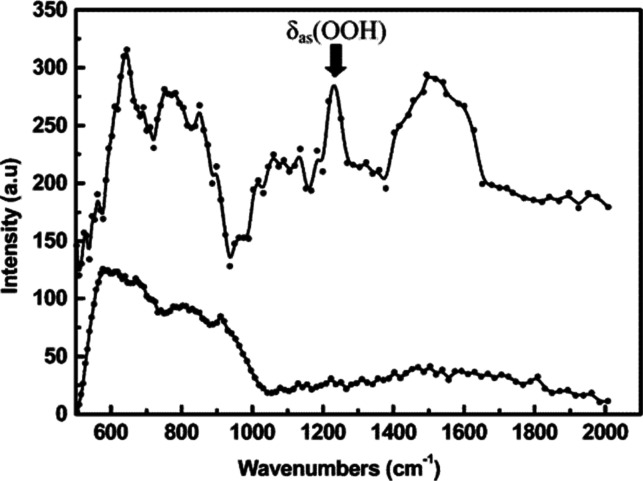
(Top) INS spectrum of Au/TiO_2_ reacted with H_2_ and O_2_ at 523 K for 4 h in flowing H_2_:O_2_:He (1:1:7). (Bottom) INS spectrum of water at 523 K adsorbed on Au/TiO_2_ for comparison. Reproduced with permission from ref (251). Copyright 2004 American Chemical Society.

Propylene oxide is an important chemical for synthesizing polyurethane plastics. It is traditionally produced via a multistep process with expensive oxidants and low efficiency.^276^ It is more attractive to carry out the direct vapor-phase conversion of propylene to propylene oxide using molecular oxygen and hydrogen. Some catalysts, including supported Au, have shown promise in the direct route, but the mechanism still needs to be discovered. An important goal in defining the mechanistic pathway of any reaction is to determine the nature of the surface species/intermediates present under reaction conditions. Sivadinarayana et al. used INS to provide the first direct spectroscopic evidence for surface hydroperoxyl species formed during the vapor phase H_2_–O_2_ reaction over Au/TiO_2_.^251^ As shown in Figure 39, except for the band between 500 and 900 cm^–1^ typical of the librations of bound or adsorbed water molecules on the Au/TiO_2_ surface, hydrogen peroxide species were indicated by the relatively sharp band at 1230 cm^–1^ attributed to the δ_as_(OOH) mode. The features between 1525 and 1600 cm^–1^ were assigned to a hydroperoxyl species complexed to water or possibly bound to the catalyst surface. This INS observation provided direct experimental evidence for the hydroperoxyl species proposed previously^277^ in the H_2_–O_2_ reaction.

##### 3.3.3.2 CO Oxidation

**Figure 40:**
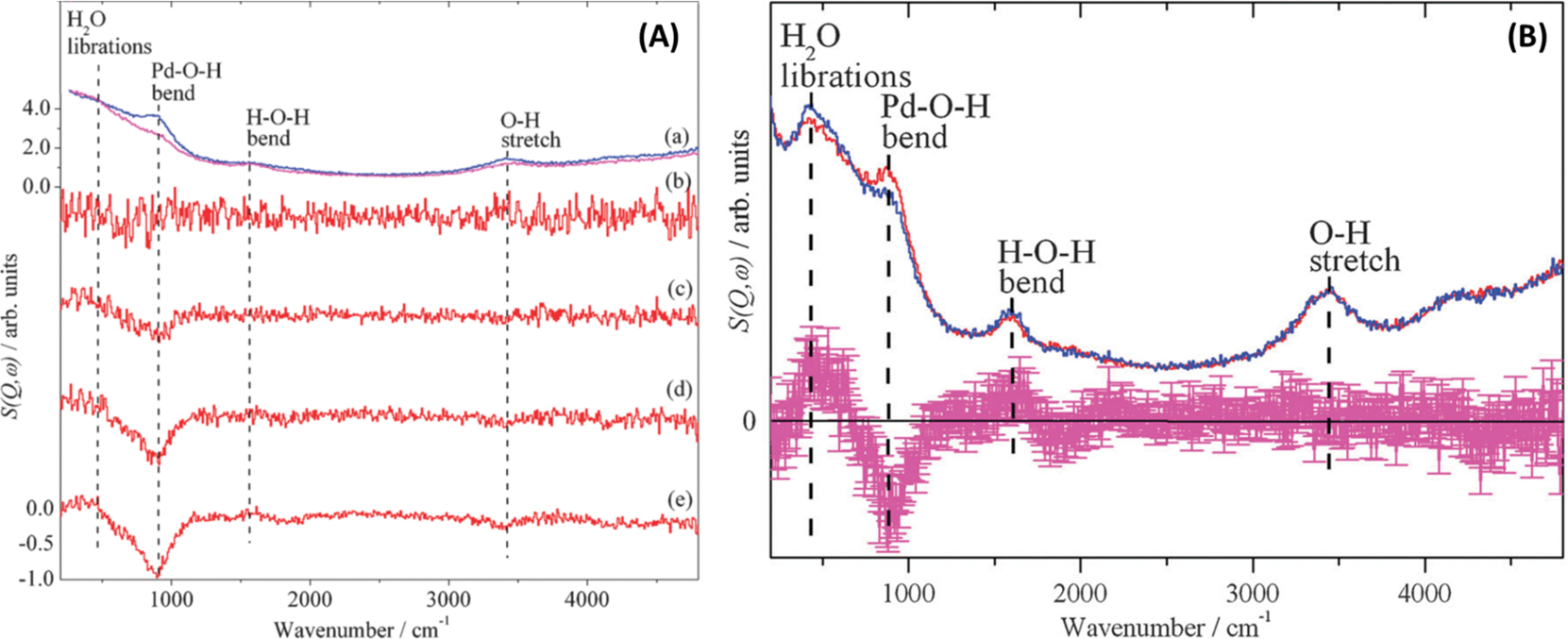
(A) INS spectra of PdO·H_2_O under flowing He/CO(5%) (100 cm^3^ min^–1^) at 25 °C at different times (*T*, hours : mins). (a) *T* = 00 : 00 (blue) and *T* = 21 : 08 end of run (magenta). Difference spectra (all ×4 ordinate expanded relative to (a)) generated by subtraction of the *T* = 00 : 00 spectrum from that at time *T*, (b) *T* = 00 : 32, (c) *T* = 01 : 08, (d) *T* = 02 : 18, (e) *T* = 21 : 08, end of run. Spectra recorded at 25 °C. (B) INS spectra of PdO·H_2_O before (red) and after (blue) reaction with CO(100%) at 25 °C. The difference spectrum (magenta) is shown ×4 ordinate expanded. Positive-going bands correspond to an increase in the species, and negative-going bands to a decrease. Spectra recorded at 5 K. Reproduced with permission from ref (73). Copyright 2011 Royal Society of Chemistry.

Low-temperature CO oxidation is critical for fuel cell application by removing CO from H_2_ generated from methane reforming and emission control catalysis.^278^ Pd is among the active low-temperature catalysts for CO oxidation in which hydroxyl groups associated with Pd are essential. Although infrared and Raman spectroscopy can readily detect the OH groups, quantification of them is challenging because the intensity of each type of OH group can be affected by different electronic factors. In contrast, the INS intensity of OH modes depends only on the density of OH groups but not on their chemical nature, i.e., all OH groups have the same neutron cross section. This virtue was used to quantify the OH groups on a Ni/Al_2_O_3_ catalyst for methane dry reforming.^256^ Parker further took advantage of INS to investigate the role of OH groups in CO oxidation over PdO·H_2_O and provided the first example of a room temperature INS study of a catalytic reaction.^73^ As shown in Figure 40A, the INS spectra recorded for PdO·H_2_O *in situ* at 25 °C under flowing He/CO(5%) at different times showed the gradual decrease of characteristic modes of hydroxyls (bending mode at 936 cm^–1^, bending mode overtone at 1850 cm^–1^, O–H stretch at 3450 cm^–1^) with the gradual increase of modes from water (librational bands at 300–600 cm^–1^, H–O–H scissors at 1600 cm^–1^, and O–H stretch at 3450 cm^–1^). To improve sensitivity, INS spectra collected at 5 K were shown in Figure 40B, along with the difference spectrum between prior- and postreaction of CO oxidation over PdO·H_2_O. Interestingly, the difference spectrum showed that the total number of O–H oscillators was unchanged, even though the number of hydroxyls and water molecules had decreased and increased, respectively. The author rationalized the observations as hydroxyls were converted to water. Thus, the reaction was stoichiometric in hydroxyls rather than catalytic: CO + 2OH → CO_2_ + H_2_O.

Last but not least, this work is of particular significance because it demonstrates that it is possible to use INS to study a working catalyst in real time at ambient temperature for the first time. This is only possible because of the high sensitivity and the ability to access low momentum transfer offered by the direct geometry spectrometer (MAPS).

### 3.4 INS Studies of Oxygen- and Nitrogen-Containing Species

While neutron scattering is especially suited to study hydrogen, it can also be used to study other light elements such as oxygen and nitrogen. Although 1 order of magnitude lower than that of hydrogen, the neutron scattering cross section of oxygen or nitrogen is still comparable to other common elements found in catalysts such as transition metals^44^ (unlike for X-rays where scattering from transition metals overwhelms that of the light elements). This makes INS potentially valuable for studying catalytic processes associated with oxygen/nitrogen-containing (and non-hydrogen-containing) species. However, due to the comparably weaker scattering from O and N and limited neutron flux, a larger specific surface area is usually required for direct observation. An alternate approach is to measure the influence of the oxygen/nitrogen species on a hydrogen-containing surface (i.e., indirect observation).

**Figure 41:**
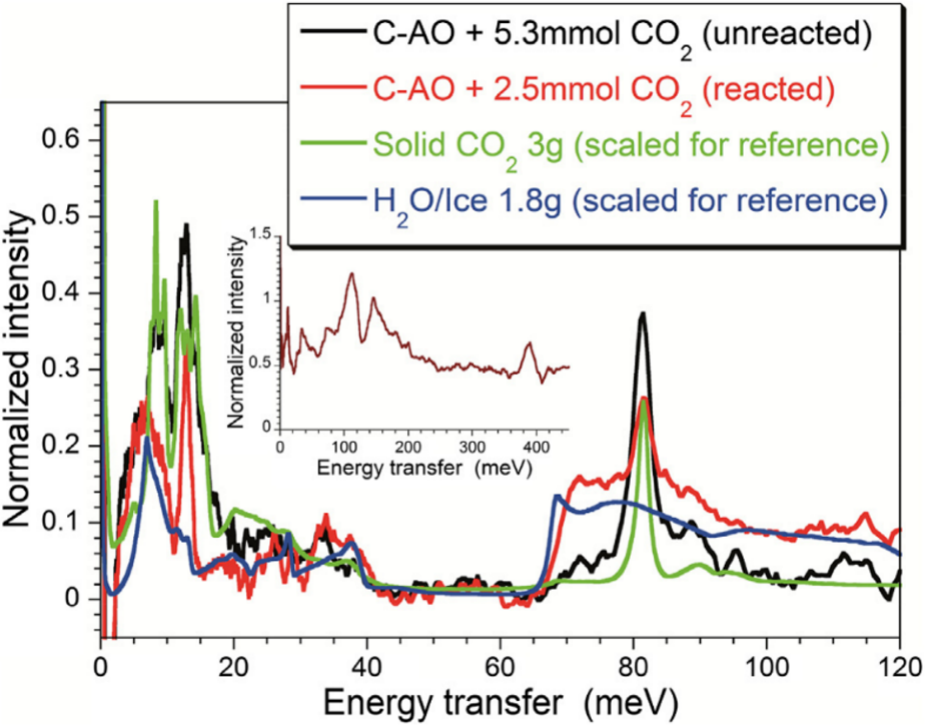
Difference INS spectra before and after CO_2_ dosing in C-AO, in comparison with the reference spectra for bulk solid CO_2_ and H_2_O. The signal from the background and the blank C-AO has been subtracted. The inset shows the INS spectrum of the blank C-AO before dosing. Reproduced with permission from ref (279). Copyright 2016 Elsevier.

In a study of CO_2_ interaction with nanoporous functionalized carbon (labeled as C-AO), INS was used to observe CO_2_ adsorption and its reaction with the surface groups to form H_2_O.^279^ Specifically, the presence of solid-like CO_2_ was observed in the unreacted sample (black curve in Figure 41), as indicated by the signature bending peak at 80 meV (645 cm^–1^). After heating to room temperature and holding for 1 h to allow for reaction, CO_2_ was consumed, as shown by the lower intensity in the red curve. In the meantime, the signal from water/ice was observed (librational edge near 67 meV (540 cm^–1^)). Also noted was the much-reduced translational band of CO_2_ (between 5 and 20 meV (40 and 161 cm^–1^)), indicating that the CO_2_ in the system was much less solid-like. In addition, compared to bulk ice, the librational edge of the reaction product had a blue-shift, which could be resulted from confinement from its adsorption environment. Before the reaction, the blank catalyst (C-AO) was also characterized with INS, and the spectrum in the inset of Figure 41 was consistent with terminating sp^2^ C–H bonds.

**Figure 42:**
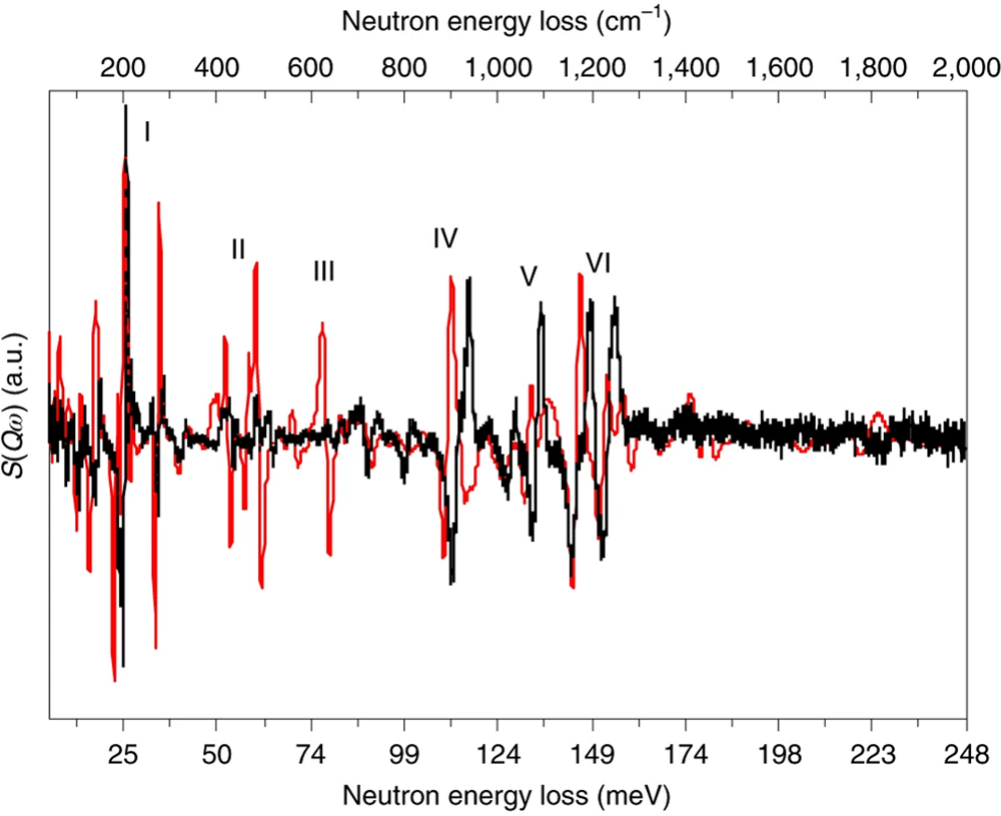
Comparison of the experimental (black) and DFT calculated (red) difference INS spectra between bare and upon NO_2_-dosing MFM-520. Reproduced with permission from ref (280). Copyright 2019 Nature Publishing Group.

In another study,^280^ a MOF (MFM-520) was found to adsorb NO_2_ and subsequently catalyze the reaction of NO_2_ to HNO_3_ (2NO_2_ + H_2_O + 1/2O_2_ → 2HNO_3_). It thus had great potential for air purification and converting the pollutant into valuable chemicals. INS was used to study the nature of the interaction between NO_2_ and the MOF. In this case, the focus was the effect of NO_2_ adsorption on the spectrum of the MOF since the INS signal from NO_2_ itself was negligibly small compared to the signal from the MOF. By comparing the experimentally measured and simulated difference spectra (i.e., the spectrum after NO_2_ dosing minus the spectrum before NO_2_ dosing) in Figure 42, one could make an assignment of the peaks and trace the origin of the changes. Specifically, changes observed for peaks I–III in the low energy region (<80 meV (645 cm^–1^)) correspond to deformational modes of the pyridine ring, and changes in peaks IV–VI in the high energy region (110–150 meV (887–1210 cm^–1^)) correspond to −CH wagging/scissoring modes. These changes were indications of direct interaction between adsorbed N_2_O_4_ and the soft −CH groups.

## 4 Neutron Powder Diffraction of Catalysis

### 4.1 Introduction to Neutron Diffraction

Powder diffraction has been widely used to study functional materials, such as catalysts and electrode/electrolyte materials for batteries and fuel cells, in the past few decades.^20,281−293^ Although the three-dimensional structural information is collapsed into one-dimensional form in the powder diffraction data, the easy and fast data collection and better adaptability have made powder diffraction popular in studying complex materials.^294^ It becomes particularly useful when high-quality single crystals are not available or where the functional materials are operating in polycrystalline forms.^294^ One attractive application of powder diffraction is structural studies of catalytic materials, where the catalysts are often used in powder (e.g., nanocrystalline) or thin film forms. The versatile sample environments of powder diffraction also allow the *in situ*/*operando* monitoring of structural changes during the catalytic reactions under operating conditions, such as high temperatures or flowing gas.^294^

Because neutrons interact (for nuclear scattering) with matter by nuclear forces instead of electromagnetic force (as for X-rays), (coherent) nuclear scattering lengths are thus isotope sensitive instead of atomic number sensitive.^42^ Therefore, neutron diffraction has unique advantages in revealing the structure of materials containing light elements, such as H, C, and O, which are important components of many heterogeneous catalysts.^34^ In many cases, it also provides good contrast that enables neighboring elements (e.g., Mn, Fe, and Cu) to be distinguished. This attribute is helpful in many multiple-element (or entropy-stabilized) catalysts.^295−297^ The ability to perform isotope substitutions for a particular element (of which hydrogen/deuterium is by far the most important) is one of the unique advantages of neutron diffraction relative to X-ray or electron diffraction. This allows unambiguous determination of atomic coordination environments in complex composition materials. In addition, it is also nondestructive, revealing structural information of catalysts during the catalytic reactions, without the concerns of beam damage that is a potential problem for synchrotron studies.^34^

#### 4.1.1 Instrumentation and Data Analysis

**Figure 43:**
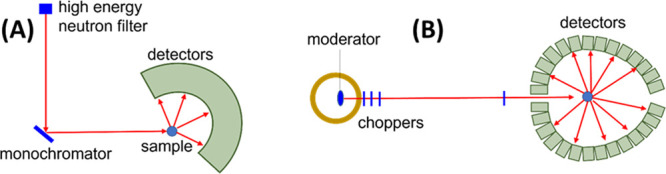
Illustration of the layout of (A) CW powder neutron diffractometer at a reactor source, and (B) ToF neutron powder diffractometer at a spallation neutron source. Only key components are drawn for simplicity.

Neutron powder diffractometers are built at both reactor and pulsed neutron sources. The former usually operates in the constant wavelength (CW) configuration,^298,299^ while the latter operates in the time-of-flight (ToF) configuration (Figure 43).^300−306^ In recent years, the development of accelerator-based pulsed neutron sources allows neutron powder diffraction data to be collected with unprecedented high count rates and high resolution.^291,301^ In addition, the latter configuration also allows complete data collection at fixed scattering angles, a feature that benefits *operando*/*in situ* diffraction measurements of catalysts during the reaction by simplifying the construction of reaction cells/vessels.^281^

**Figure 44:**
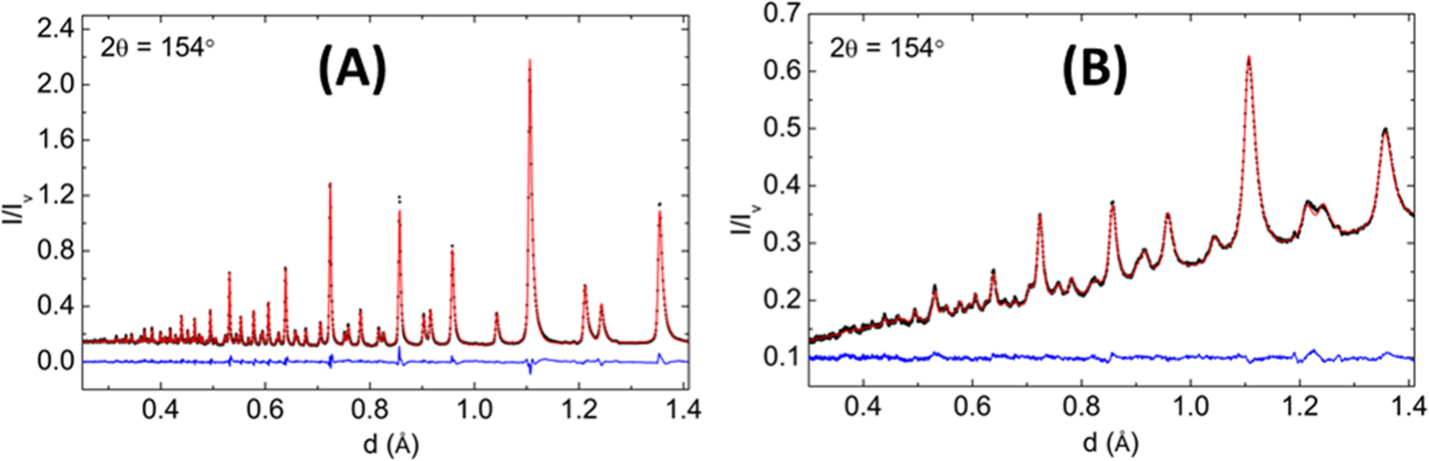
Rietveld refinements of the structure of prereduced ceria nanocube (A) and nanorod (B) using neutron powder diffraction data (only the high resolution back scattering bank is shown here). Frenkel type oxygen defects are included in the refinement. The increasing (and relatively high) background as a function of *d*-spacing can be seen in the nanorod data (B), suggesting the presence of appreciable amounts of surface hydroxyl groups in the prereduced ceria nanorod sample. Reproduced with permission from ref (283). Copyright 2021 American Chemical Society.

Quantitative structure analysis of neutron Bragg diffraction data by whole pattern fitting methods, such as Rietveld refinement, provides accurate long-range structural information such as lattice parameters, atomic positions, site occupancies, and, to some extent, atomic displacements.^307,308^ It can also provide helpful microscopic information about powder specimens, such as mean crystallite size and microstrain.^309^ One particularly fruitful application of neutron powder diffraction for catalytic research is in locating the positions of light atoms, such as oxygen defects in oxides or hydrogen in metal hydrides or porous materials.^102,310,311^Figure 44 shows the ToF neutron diffraction patterns (from NOMAD, SNS) obtained from ceria nanocubes and ceria nanorods. Since neutron scattering is highly sensitive to oxygen, it can be used to accurately extract the location and site occupancies of oxygen. It can also be seen that the Bragg peaks are much broader for the nanorod sample relative to the nanocube sample due to the much smaller crystallite size (coherent column lengths) of the nanorods.^283^

In a neutron powder diffraction experiment, the observed scattering intensity is the integration of the dynamic scattering function *S*(*Q*,*E*) through a broad bandwidth of scattered neutron energies. It is related to the instantaneous real-space atomic correlation *G*(*r*,*0*) by a sine Fourier transform of the scattering function in momentum transfer space.^312,313^ Powder neutron diffraction signals are often categorized into Bragg diffraction and diffuse scattering components. The former contains the information of the time-averaged long-range static structure, while the latter can be due to either the structure dynamics (e.g., thermal diffuse scattering) or short-range structural ordering phenomena (e.g., short-range structure distortion, chemical or magnetic ordering, etc.).^314,315^ Modern neutron total scattering experiments are usually carried out at spallation neutron sources,^313^ where the high flux of high energy epithermal neutrons can be utilized, which is critical in realizing measurements of the scattering to very high momentum transfer (e.g., >30 Å^–1^). Total scattering is possible at reactor sources, but the absence of high energy neutrons limits the available *Q*-range (and hence the real space resolution). D4 at the ILL is an exception, as this views the hot source, so it has a maximum *Q* ∼ 24 Å^–1^.

To obtain good real space resolution and realize complete structure coverage, total scattering often uses very large bandwidth wavelengths of neutrons. The neutron energies used for these studies can range from meVs to several eVs. Thus, the energy nondiscriminated measurements of total neutron scattering contain both static and dynamic information. The structure analysis of total scattering data can be either carried out in reciprocal space through modeling the diffuse scattering signal or Fourier transformed into the real space pair distribution function (PDF) data. For nanosized catalysts, total neutron scattering is a powerful tool for studying both the average bulk structure and potential surface defect structure. The volume ratio of surface/subsurface to bulk is much higher in nanomaterials than in bulk materials, making PDF an ideal tool for studying the surface and bulk structures.^283,285,316^ To view these different bulk and surface structure features more clearly, it is often more convenient to show the structure in real space, i.e., using the PDF method, rather than inspecting the broad diffuse signal in reciprocal space scattering data.

**Figure 45:**
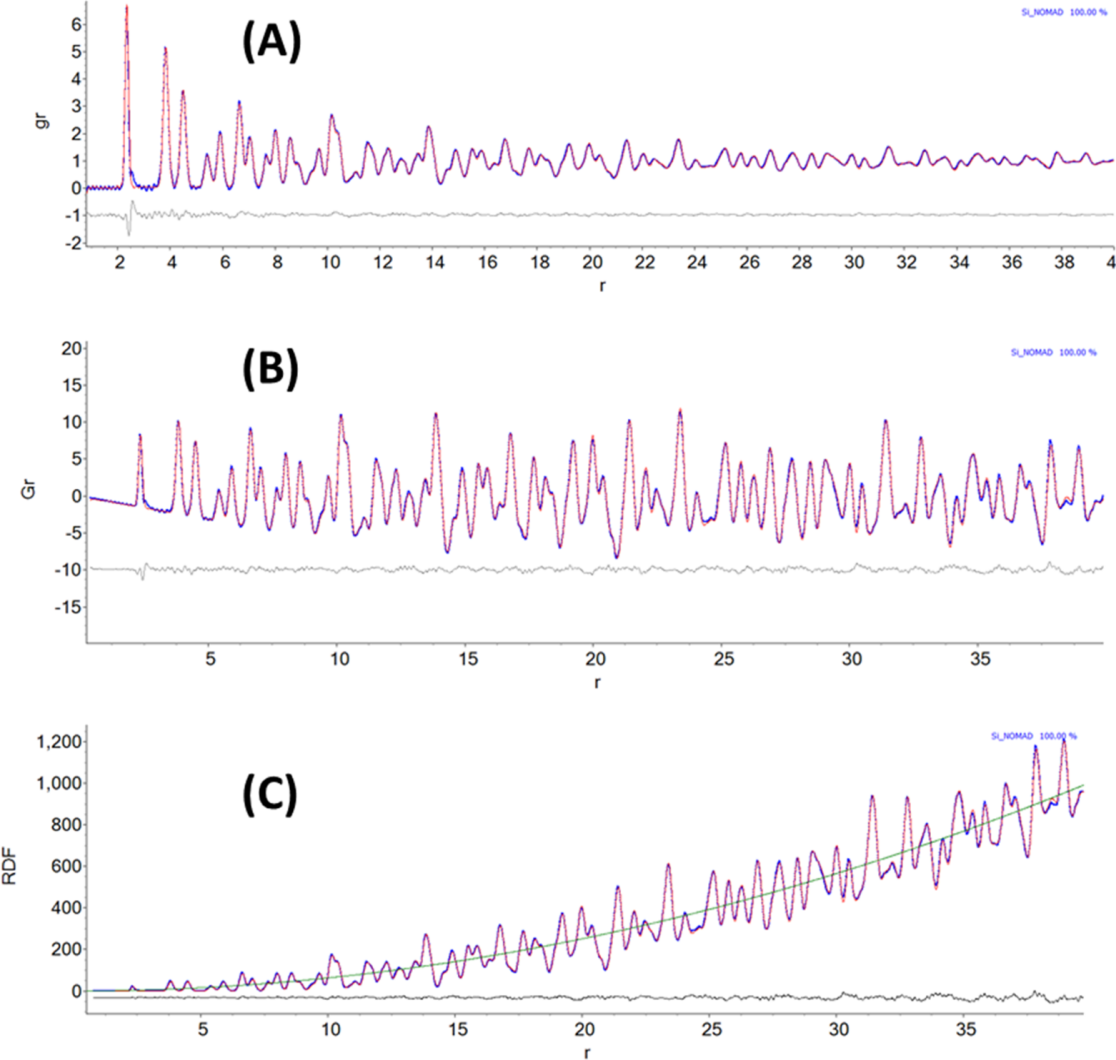
Different types of commonly used (neutron) pair/radial distribution functions of Si. Pair distribution function g(r) (A), reduced pair distribution function G(r) (B) and radial distribution function R(r) (C). The experimental data are shown in blue dots, calculated results from small-box modeling are shown in red, and the difference curve is shown in gray. The dotted green curve shown in (C) is the average number density of Si, which is equal to 2πr^2^ρ_0_.

PDF g(r) describes the probability of finding an atomic pair at specific distances r. Therefore, it is zero at atomic distances smaller than the first atomic pair and is approaching one at very large distances (Figure 45a). There are also other forms of pair/radial distribution functions used by different communities to emphasize various aspects of the investigated structural features.^317,318^ For crystalline or nanocrystalline materials, a more balanced display of both short and intermediate-range structure is preferred by introducing the reduced pair distribution (Figure 45b) function G(r). The other helpful expression is the radial distribution function R(r) (Figure 45c), where the integrated peak intensity (here only for a monatomic system) represents the coordination number for the corresponding atomic pairs

#### 4.1.2 The Development of *In Situ/Operando* Neutron Diffraction/PDF for Catalytic Research

The report from Ozawa and Loong is probably the earliest work using *in situ* neutron diffraction to study the reduction reaction of CeO_2_ catalysts.^319^ With the increases in neutron flux in modern spallation neutron sources, it has become routine to monitor the structural changes of catalysts during catalytic reactions using *in situ*/*operando* neutron diffraction and even neutron total scattering. Advanced sample environments such as gas-flowing systems or reaction (e.g., high pressure) vessels are often needed to realize these capabilities. Dedicated gas-flow systems were reported to operate in several neutron diffractometers worldwide.

**Figure 46:**
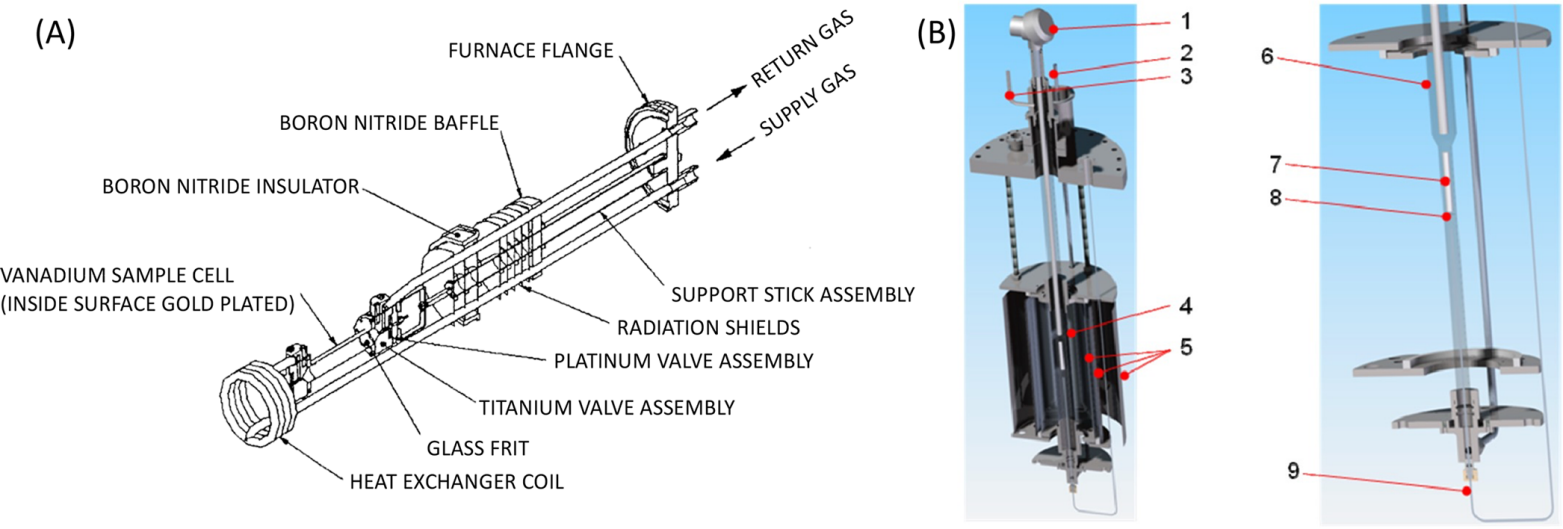
(A) Sectional view of the catalyst furnace center-stick assembly for the ISIS neutron diffraction *in situ* gas flow sample environment. Reproduced with permission from ref (320). Copyright 1999 AIP Publishing. (B) The schematic illustration of the new ISIS high-temperature flow through the gas cell. Reproduced with permission from ref (321). Copyright 2010 IOP Publishing Ltd.

One of the early gas flow systems was reported by Turner et al. (Figure 46A).^320^ Vanadium sample cans were used, and a thin layer of gold was deposited on the internal surfaces to prevent the oxidation of vanadium. Four separate gas lines feeding into the common mixing vessel were constructed. The gas supply line was first taken into the vanadium sample cell, and the outgoing gas was allowed to pass into the analyzing line. They also built a separate vacuum line that could be connected to each circuit. Later, an improved high-temperature gas flow cell was developed at ISIS with a quartz sample holder (Figure 46B).^321^ This allowed gas to flow through the cell at a much higher temperature, e.g., up to 1300 K. This system has been successfully used to study the active site of NiNa–Zeolite Y catalysts during its reaction with acetylene.^18^

**Figure 47:**
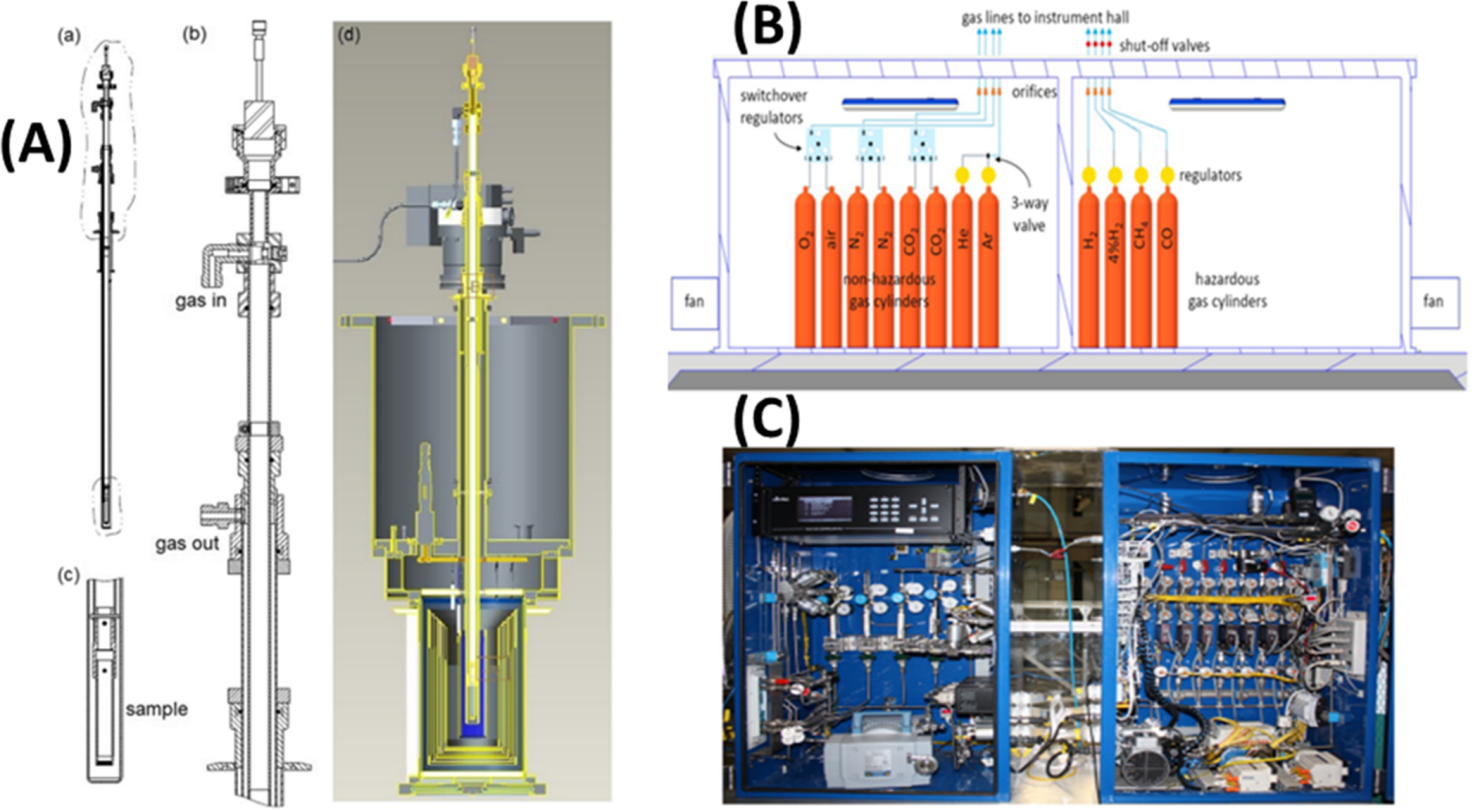
(A) Cross section of the quartz gas doping insert. (B) Schematic of the gas outbuilding for the POWGEN gas flowing system. (C) Two gas mixing cabinets, with the hazardous gas cabinet on the left and the nonhazardous gas cabinet on the right. Reproduced with permission from ref (322). Copyright 2018 AIP Publishing.

**Figure 48:**
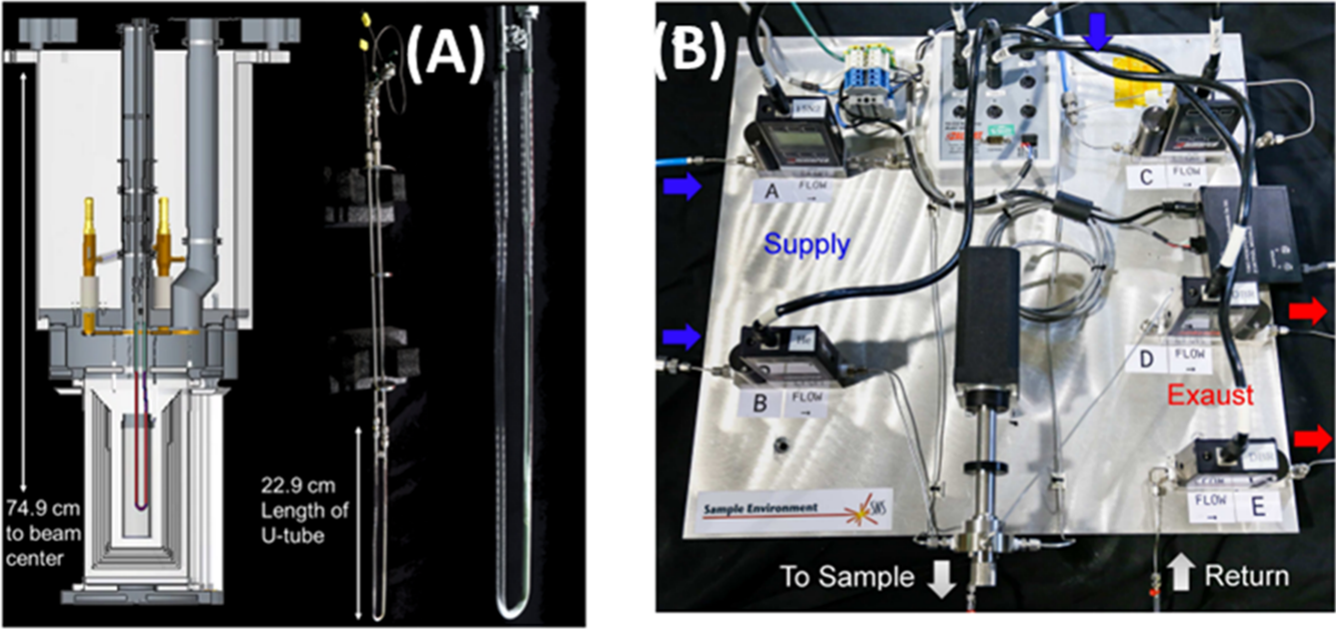
(A) Quartz sample U-tube for high temperature gas flow cells on NOMAD. (B) Image of the flow controlling apparatus on NOMAD. Reproduced with permission from refs (325, 326). Copyright 2017 and 2018 AIP Publishing.

More recently, gas-flowing/doping systems have also been developed and commissioned at SNS’s two neutron powder diffractometers. At POWGEN, an integrated automated gas environment system (AGES) was commissioned (Figure 47), which allows the control of both gas flow and temperature (up to 850 °C).^322^ The neutron diffraction data can be collected by concurrently measuring the effluent gas with a mass spectrometer. This system has been widely used to study fuel cell electrode/electrolyte materials, particularly concerning the potential oxygen diffusion pathways in these materials.^323^ It can also monitor structural transitions during solid-state chemical synthesis.^324^ At the NOMAD beamline, a high-precision gas flow cell with a high temperature sample environment was developed (Figure 48). This sample environment can be used for both *in situ* neutron Bragg and total scattering (PDF) studies under dynamic gas flow conditions. It allows fast gas switch (<425 ms) and fast gas flow rates (up to 50 mL/min).^325,326^ This allows a stroboscopic isotope contrast experiment to be carried out.^327^ This sample environment can also be used to monitor the chemical synthesis or thermal decomposition process.^326^ An improved version of this gas-flowing sample environment, with improved gas flow control, use of hazardous gases, and more accurate positioning of reaction cells, is currently under development at the beamline.

### 4.2 ND Studies of Surface and Bulk Oxygen and Vacancies

Defect engineering has been proven to be an effective strategy for altering the catalytic properties of materials.^328^ To obtain a catalyst with desired properties, it is essential to understand how different defects regulate the structure and properties of a catalyst. In a catalytic system in the form of supported metal nanoparticles, the defects affecting catalytic events could be on the surface of metal nanoparticles or on the support surface near the active metal sites. For the latter, the most seen defect is oxygen vacancies (O_v_) in oxide supports. Due to the complexity and diversity of defect structures, a combination of spectroscopic and microscopic techniques are required to identify defects, quantify their concentration, distinguish defect distribution, and determine the local structure around defects.^328^ Among these techniques, ND is useful for detecting surface and bulk oxygen and vacancies due to the high penetration capability of neutrons and high neutron scattering cross section of light atoms.^329,330^ In addition, complementary results can be obtained by performing PDF analysis on the local to the intermediate structure of nanomaterials with different types of defects or short-range chemical order.

Ceria has been heavily investigated in defect engineering due to its reducibility, involvement in redox reactions, tunable surface, and relatively strong interaction with supported nanostructures. By performing refinement of single crystal ND data collected at ambient temperature, Kümmerle and Heger et al. could determine the crystal structures of CeO_1.68_, Ce_7_O_12_, and Ce_11_O_20_ with ordered oxygen vacancy distributions.^331^ Sun et al. found that the separately prepared Cu (4.8 ± 0.6 nm) nanocrystals could reconstruct and change oxidation state after being deposited on a ceria (5.9 ± 0.7 nm) support.^284^ Based on the ND and PDF results, Cu (≤20 mol %) incorporation did not alter the lattice parameter of ceria. However, the interfacial restructuring occurred and was associated with the creation of surface defects on ceria, which enhanced the activity of catalysts for the water–gas shift reaction.^284^ As a follow-up, Sun et al. were able to introduce more defects in ceria by building surface-confined high-entropy oxide (HEO) layers.^285^ When comparing the series of CeO_2_, Cu-CeO_2_, CuCo-CeO_2_, and CuCoFeNiMn-CeO_2_, ND analysis suggested a similar amount of interstitial O_v_ in the bulk of these oxides. Taking advantage of the sensitivity of NPDF to the short- and intermediate-range structures at the nanoscale, it was shown that the transition metal substituents mostly formed a separate phase on ceria and impacted the surface density of O_v_ of the system. The Ce_7_O_12_ phase provided the best fit of the PDF patterns, demonstrating the enrichment of surface-confined, oxygen-deficient phases. The density of the Ce_7_O_12_-like O_v_ followed the sequence CeO_2_ (<1%), Cu-CeO_2_ (3.0%), CuCo-CeO_2_ (5.0%), and CuCoFe-NiMn-CeO_2_ (13.4%), this correlated with the enhanced activity of CuCoFeNiMn-ceria for the CO oxidation reaction.^285^ The results manifested two orthogonal strategies to modulate the amount of the bulk and surface oxygen defects in ceria. By tuning the geometric parameters such as size and shape, the bulk intrinsic interstitial Ov could be modified, as demonstrated below.^283^ This work shows that modulating the number of transition-metal substituents can control the extent of extrinsic, surface-confined O_v_ on ceria.

**Figure 49:**
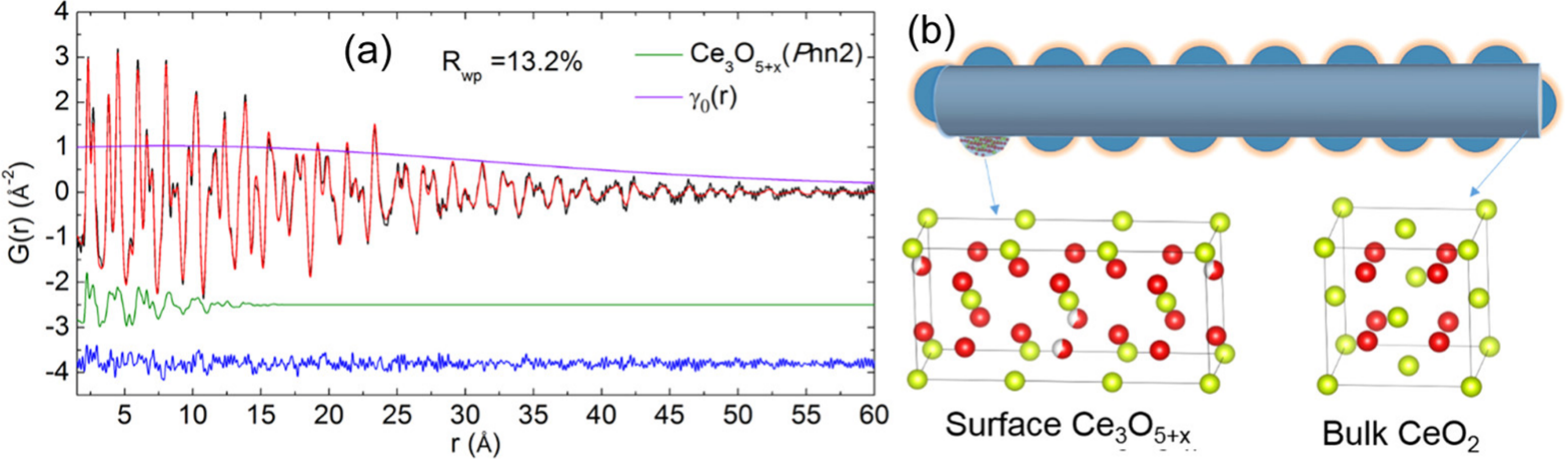
(a) Two-phase modeling of ceria nanorods (CeO_2_ with Frenkel-type defects and *P2nn*-type Ce_3_O_5+*x*_) with the numerical nanorod shape correction. The contribution from surface Ce_3_O_5+*x*_ is highlighted in olive (shifted for clarification). The experimental data is shown in black, calculated curve in red, and difference curve in blue. The numerical approximation of the envelope function of the nanorod shape [γ0(r)] is shown in purple. (b) Schematic illustration of the small spherical Ce_3_O_5+*x*_ nanoparticles decorating the surface of ceria nanorods (assuming that there is no interatomic interaction between these two phases). Crystal structures of the bulk fluorite CeO_2_ phase and the surface defect Ce_3_O_5+*x*_ phase are shown at the bottom. Reproduced with permission from ref (283). Copyright 2021 American Chemical Society.

The oxygen defects in oxides not only form during synthesis but also respond to external environment change. By combining ND and PDF, Luo et al. revealed the nature of the surface and bulk defect sites in ceria nanocrystals (nanorod and nanocube) when annealed at high temperatures and after exposure to SO_2_.^283^ As shown in Figure 49, the surface oxygen defects were predominantly the partially reduced Ce_3_O_5+*x*_. The bulk defect structures were dominated by interstitial Frenkel-type O_v_. Interestingly, annealing the nanorod sample at 600 °C in a vacuum, Ce_3_O_5+*x*_ with long-range oxygen vacancy ordering was observed. In addition, upon exposure to SO_2_, a drastic decrease in the surface vacancies in the ceria nanocrystals was observed.^283^ These results suggested the dynamic evolution of oxygen defects in responding to the change in external environments. Such a change might affect the catalytic behavior of catalytic systems involving ceria supports.

The investigations of dynamic characteristics of defect sites in oxides require the application of *in situ* techniques. Taking advantage of the large X-ray fluxes available for kinetic studies, the keen sensitivity to oxygen displacements of neutrons, and the efficiency of time-of-flight diffraction, Ozawa and Loong studied redox behavior in ceria-containing oxide catalysts.^319^ An analysis of the X-ray data collected under a CO/N_2_ atmosphere at 500–700 °C was used to characterize the reduction kinetics for CeO_2_ and Ce_1–*x*_La_*x*_O_2–*x*/2_. In comparison, ND measurements on Pt-impregnated Ce_0.1_Zr_0.9_O_2_ under both CO/Ar and O_2_/Ar atmospheres up to ∼700 °C showed that Pt-impregnation accelerated the reduction of Ce^4+^ to Ce^3+^ first on the interface of the metal and oxide particles. The generated Ov subsequently migrated to the bulk of the oxide.^319^ Li et al. studied Ce_0.8_Y_0.2_O_1.9−δ_ by performing *in situ* time-of-flight ND at 900 °C in the oxygen partial pressure *p*O_2_ range from 10^–1^ to 10^–18^ atm.^332^ The data showed that the lattice parameter moderately increased with decreasing *p*O_2_ in the range of *p*O_2_ > 10^–14^ atm, while a dramatic expansion (∼0.6%) of the fluorite structure occurred at a *p*O_2_ of 10^–18^ atm. In addition, an approximately linear relationship between the lattice parameter and oxygen vacancy δ was observed.^332^

In addition to ceria, ND has been used to study O_v_ on other oxides. Cox-Galhotra et al. studied the crystal structure of PrBaCo_2_O_5+δ_ at high temperatures and controlled oxygen partial pressures by employing *in situ* ND.^333^ According to the results, the O_v_ were found localized within the Pr layer, with total oxygen stoichiometry between 5.57(1) and 5.17(2). The location of these vacancies and the anisotropic displacement of the surrounding oxygen anion sites indicated that ion transport occurred via a hopping mechanism between O sites in the Pr layer and the nearest neighbor sites in the Co layer.^333^ Tonus et al. investigated the chemical reduction of the K_2_NiF_4_-type oxides, *Ln*_2_Sr_2_CrNiO_8-δ_ (*Ln* = La, Nd) and Nd_2.25_Sr_1.75_CrNiO_8−δ_*in situ* under a dynamic hydrogen atmosphere at high temperatures using ND.^334^ The results showed that hydrogen reduction of the K_2_NiF_4_-type materials, *Ln*_2_Sr_2_CrNiO_8−δ_, proceeded via oxygen deintercalation from the (Cr/Ni)O_2_ layers; the occupancy of the oxygen site within the rocksalt (*Ln*/Sr)O layers remained unchanged throughout the heating/cooling cycle under H_2_-gas.^334^ The reduction of La_2_Sr_2_CrNiO_8−δ_ first yielded a pure Ni(II) phase, La_2_Sr_2_CrNiO_7.5_, and then a mixed Ni(II,I) oxide, La_2_Sr_2_CrNiO_7.40_. In contrast, hydrogen reduction of Nd_2_Sr_2_CrNiO_8−δ_ and Nd_2.25_Sr_1.75_CrNiO_8−δ_ proceeded continuously from Ni(III) to an average oxidation state of 1.80 for the nickel ion.^334^ By using ND, Ozawa et al. found that at 900–1000 °C 10 mol % CuO was doped into a γ′-phase alumina, and this CuO-alumina catalyst showed a 20% lean de-NOx removal efficiency in a test using a model exhaust gas mixture of space velocity = 100,000 h^–1^.^335^ The γ → θ → α phase transformation was also observed in La-doped (1 mol %) Al_2_O_3_ powders by *in situ* ND from 500 to 1300 °C.^336^

Briefly, in this section, we demonstrate that oxides as support materials or catalytic species can be quite complex in catalysis studies: oxygen atoms and defect sites are easily affected by dopants/supported metals or by changing external environments. ND has displayed its power in studying oxygen and defects in oxides with catalytic implications as a complementary tool. The obtained results can be linked with the local structures of active species/sites and their working mechanisms.

### 4.3 ND of Catalyst Structural Transformations During Catalysis

ND is a powerful tool for studying the structural transformation of catalytic materials in working conditions for two reasons.^287,319^ First, as a bulk sensitive tool, ND can provide real-time integral information on structures such as phase compositions, domain sizes, lattice strain, and defects. Moreover, ND can give excellent diffraction patterns for highly symmetric materials as the neutron’s scattering power is independent of the diffraction angle.^337^ Second, due to the high penetration ability of neutrons (mm level), neutron beam interacts weakly with many materials. As a result, constructing materials of *in situ*/*operando* ND reactors can be chosen only based on the requirements of reactions. Such characteristics of neutron beams are especially important for reactions involving harsh conditions such as high temperatures and pressures. For instance, ND is one of a few available techniques that can be used to study the structure evolution of catalysts for high pressure ammonia and methanol synthesis.

#### 4.3.1 Ammonia Synthesis and Decomposition

Ammonia is of great importance in industry. As a fertilizer, ammonia plays an essential role in the agricultural industry. It provides artificially fixed nitrogen to support the sustenance of about half of the world’s population.^20^ It can also be used to produce other industrial chemicals (such as polyimides, nitric acid, pharmaceuticals, refrigerants, dyes, and cleaning solutions). Moreover, ammonia is considered as an attractive candidate for hydrogen storage due to the high volumetric (121 kg H_2_/m^3^ at 10 bar) and gravimetric (17.8 wt %) hydrogen density.^338−340^ In this section, we will focus on both ammonia synthesis and decomposition and the applications of neutron diffraction in these two processes.

The most utilized method for ammonia synthesis is the Haber–Bosch process; its main disadvantage is that it requires large amounts of energy. Specifically, to obtain acceptable ammonia yields (18 vol%) in the exhaust gas, the conventional Haber–Bosch process needs to operate at 500 °C and 200 bar using the so-called “ammonia iron” catalyst.^341^ It is estimated that 1–2% of global energy is used for ammonia synthesis.^93^ Thus, developing new catalysts to improve the process economics for ammonia synthesis is of great research interest. Currently, representative catalysts for ammonia synthesis are based on Fe, Ru, metal hydrides (e.g., VH_0.39_), metal nitrides (e.g., Ni_2_Mo_3_N), and lithium hydride–transition metal (nitride).^92,342−347^

**Figure 50:**
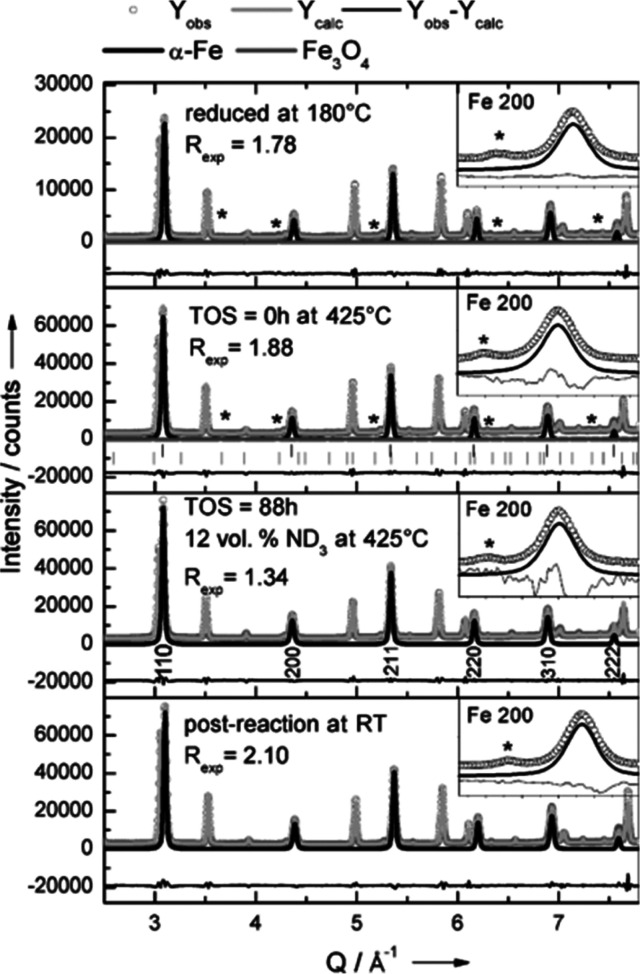
Neutron diffraction patterns of the ammonia synthesis catalyst under different conditions. The black line indicates the fitted contribution α-Fe phase to the patterns. (Top) Reduced, initial catalyst in 4.4 bar D_2_ at 180 °C (the dark gray line is the profile of magnetite, peaks additionally marked by asterisks). (Top-middle) Prereaction catalyst at 425 °C under 75 bar N_2_/D_2_ = 1/3 at TOS = 0 h. (Bottom-middle) *In situ* reaction state at 425 °C under 75 bar syngas, which is converted to yield 12 vol % ND_3_ at TOS = 88 h. (Bottom) Postreaction catalyst in 75 bar Ar at room temperature. The insets show the magnification of the 200 peak of α-Fe, wherein the black asterisks mark the contribution from the Ni reactor tube. Reproduced with permission ref (341). Copyright 2013 Wiley-VCH.

One question for NH_3_ synthesis over Fe-based catalysts is whether Fe–N phases formed in the reaction condition as the nitriding process has been observed over iron-based catalysts in NH_3_ decomposition.^348−350^ To address this question, *in situ* neutron diffraction was employed to study the “ammonia iron” catalyst at 425 °C and under 75 bar N_2_/D_2_ (1/3) mixture. The results showed the presence of the α-Fe phase rather than the nitridation of bulk Fe (Figure 50), suggesting that the pressure of N_2_ in this study was unlikely to cause the nitriding of Fe.^341^ It was proposed that the catalyst preparation, activation process and/or additives could influence the microstructure and subsequent stability of the α-Fe phase in the catalyst.

**Figure 51:**
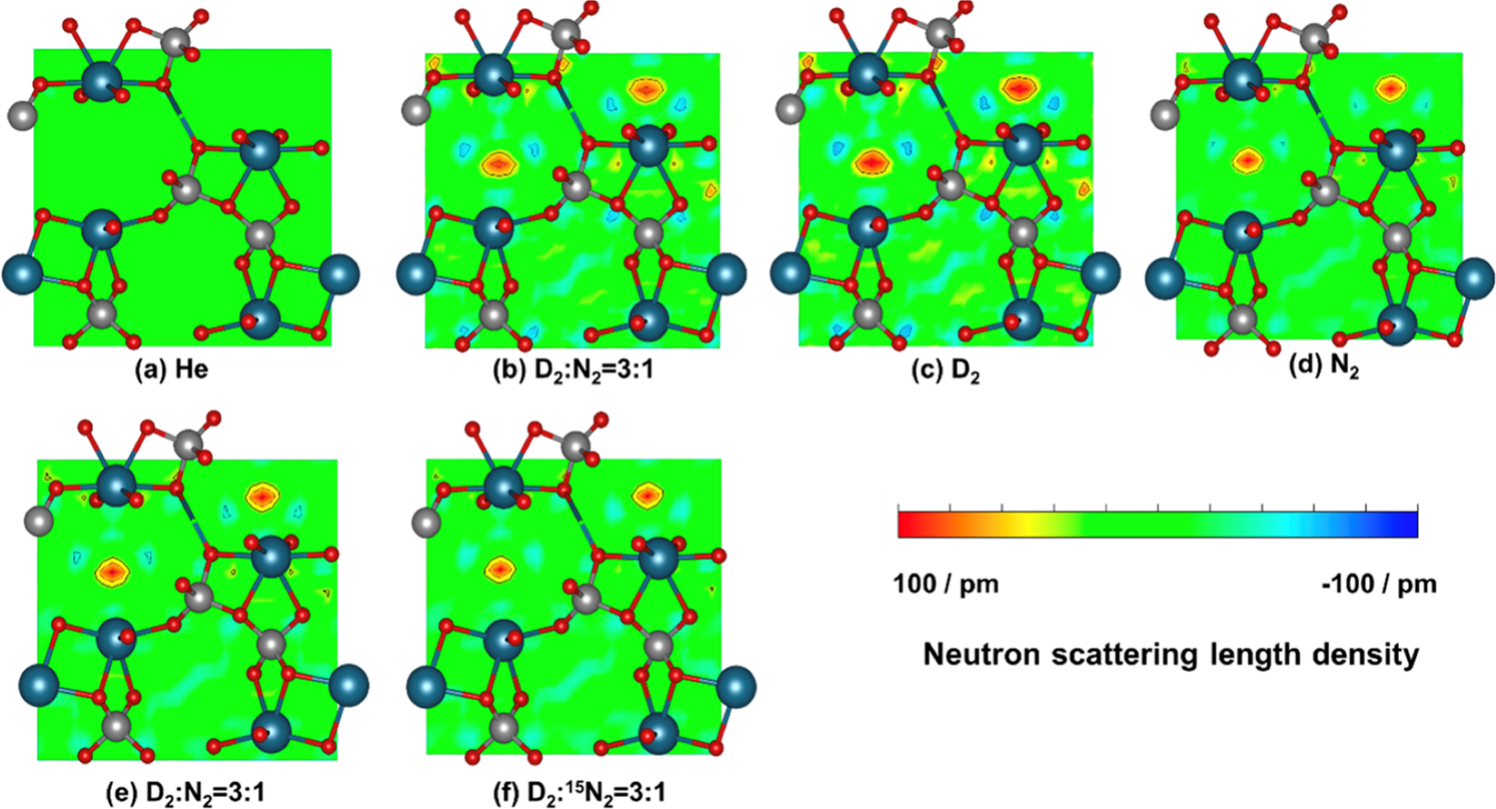
Difference Fourier maps created from results obtained using neutron diffraction. All maps are representations of the (001) planes in C12A7:e^–^. Diffraction patterns were obtained at 673 K after continuous reaction under conditions in the order (a) to (f) in 0.1 MPa gas pressure: (a) He treatment for 30 min, (b) D_2_:N_2_ = 3:1 for 3 h, (c) D_2_ for 2 h, (d) N_2_ for 2 h, (e) D_2_:N_2_ = 3:1 for 2 h, and (f) D_2_:15N_2_ = 3:1 for 2 h. Note that the scale for neutron-scattering length density is the same in all maps. Reproduced with permission from ref (20). Copyright 2020 American Chemical Society.

Ru is one of the best monometallic catalysts for ammonia synthesis due to the optimum N_2_ adsorption energy.^351,352^ However, hydrogen atoms preferentially adsorbed on the B5 sites of Ru at low temperatures (e.g., 350 °C), and that prevented the adsorption of N_2_, causing “hydrogen poisoning”.^351^ It was found that using electride and hydride materials as supports for Ru catalysts could significantly alleviate the hydrogen poisoning effect due to the presence of anionic electrons on the catalysts in the reaction.^224,225,352−355^ Kammert et al. studied the structure of Ru/C12A7:e^–^ catalysts under different environments (e.g., He, D_2_:N_2_ = 3:1, pure D_2_, pure N_2_, and D_2_:^15^N_2_ = 3:1) via neutron-scattering techniques (Figure 51).^20^ They observed an isolated D species encaged in the lattice of C12A7:e^–^ support of Ru/C12A7:e^–^ catalyst during the D_2_/N_2_ reaction. The encaged D species was stable in the applied environments once it formed at 673 K (Figure 51b–f) and thus unlikely to catalyze the reaction.

**Figure 52:**
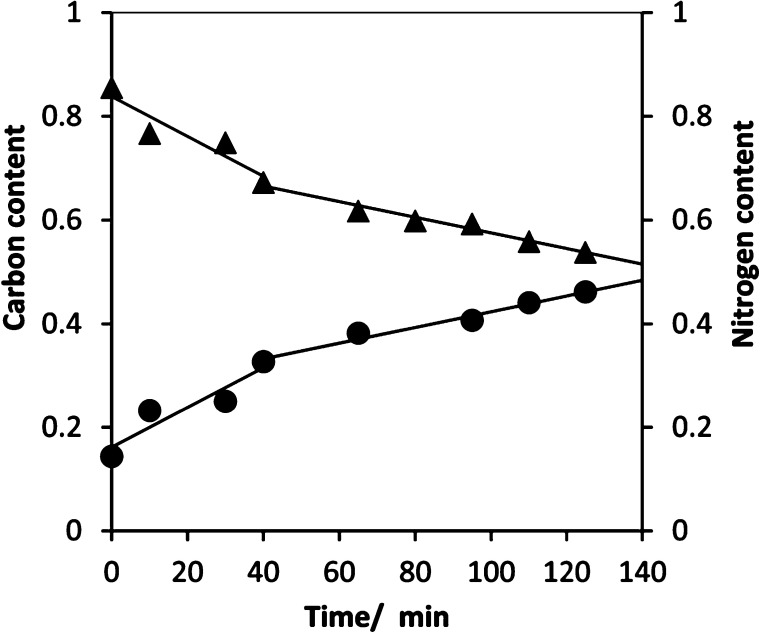
Evolution of the C/N occupancy of the 16c Wyckoff lattice site in Co_3_Mo_3_C as a function of reaction time with 60 mL min^–1^ of 75 vol% H_2_ in N_2_ (BOC, 99.98%) at 500 °C. (▲) Fractional carbon content and (●) fractional nitrogen content as determined from the Rietveld refinement against powder neutron diffraction data. Reproduced with permission from ref (93). Copyright 2017 American Chemical Society.

It was reported that Co_3_Mo_3_N catalysts exhibited about two times the ammonia synthesis rate of Fe–K_2_O–Al_2_O_3_ at atmospheric pressure and 400 °C.^356^ However, the role of N in the catalyst was unclear. When using Co_3_Mo_3_C in NH_3_ synthesis, *in situ* powder neutron diffraction indicated that nitrogen gradually replaced the carbon atoms (Figure 52).^93^ Analysis of the postreaction samples with different techniques verified that the substitution of carbon by nitrogen proceeded until close to complete substitution, suggesting that the active species was Co_3_Mo_3_N and the origin of its high activity was correlated with lattice nitrogen. It was proposed that NH_3_ synthesis over Co_3_Mo_3_N catalysts followed Mars–van Krevelen mechanism with lattice nitrogen as a key surface species.

The idea of using ammonia as a carrier for hydrogen delivery has gained much attention in recent years because ammonia has a number of favorable attributes including its high capacity for hydrogen storage and the strong hydrogen bonding between molecules, which makes it easier to liquefy ammonia than hydrogen. However, in order to use ammonia for hydrogen storage, one has to lower the energy input required for releasing hydrogen from ammonia because ammonia decomposition (cracking) is endothermic. Since the temperature required for efficient cracking depends on the catalyst, it is essential to develop efficient catalysts for releasing H_2_ from NH_3_ (ammonia cracking) at moderate temperature.

As alternatives to the use of rare or transition metal catalysts, light metal (e.g., Li, Na, K) imide–amide systems were studied because they showed high NH_3_ decomposition efficiencies over extended periods at moderate temperatures.^339^ It was suggested that light metal imide–amide systems possessed activity for ammonia decomposition via a cycle of decomposition and formation of the metal amide (MNH_2_).^339,357^ However, it was unclear about the reaction mechanism as the reaction conditions (e.g., ammonia flow rate, temperature) could influence the existing active phases.^358^ To address it, neutron powder diffraction was applied and useful information was obtained. For instance, for Na/NaNH_2_ system, neutron powder diffraction of postreaction samples showed that stoichiometric NaNH_2_ was the major phase under NH_3_ decomposition conditions.^339^ For the lithium imide catalyst, lithium-transition metal nitrides were not detected by *in situ* neutron powder diffraction under the reaction condition.^338^ Analysis revealed that the lattice parameter of the lithium imide catalyst increased when exposed to ND_3_ at 500 and 550 °C. The average stoichiometry of the Li_2_ND sample was calculated by Rietveld refinement using a continuum of stoichiometry between Li_2_ND and LiND_2_ as a single phase (Table 3). The results suggested that the lithium imide phase was converted to an intermediate phase between lithium amide and lithium imide when ND_3_ was introduced. The lithium imide phase was regenerated when ND_3_ was replaced with Ar. Adding ND_3_ at a higher temperature (550 °C) shifted the stoichiometry toward lithium amide to a lesser extent. Based on these results, it was proposed that nonstoichiometric lithium imide was the active phase in NH_3_ decomposition. A nonstoichiometric structure was also observed over a lithium–calcium imide catalyst for ammonia decomposition.^358^

**Table 3:** Refined Values for the Lattice Constant and Average Stoichiometry of Li_2_ND Sample^a^

Segment	Temp. (°C)	Gas	*a* Li_2_ND (Å)	*p* Value/Average stoichiometry
i	500	Ar	5.174(3)	0.002(33)/Li_1.998_ND_1.002_
ii	500	ND_3_	5.190(5)	0.37(3)/Li_1.63_ND_1.37_
iii	550	Ar	5.172(2)	0.19(2)/Li_1.81_ND_1.19_
iv	550	ND_3_	5.177(3)	0.24(2)/Li_1.76_ND_1.24_

a Reproduced with permission from ref (338). Copyright 2015 Royal Society of Chemistry.

**Figure 53:**
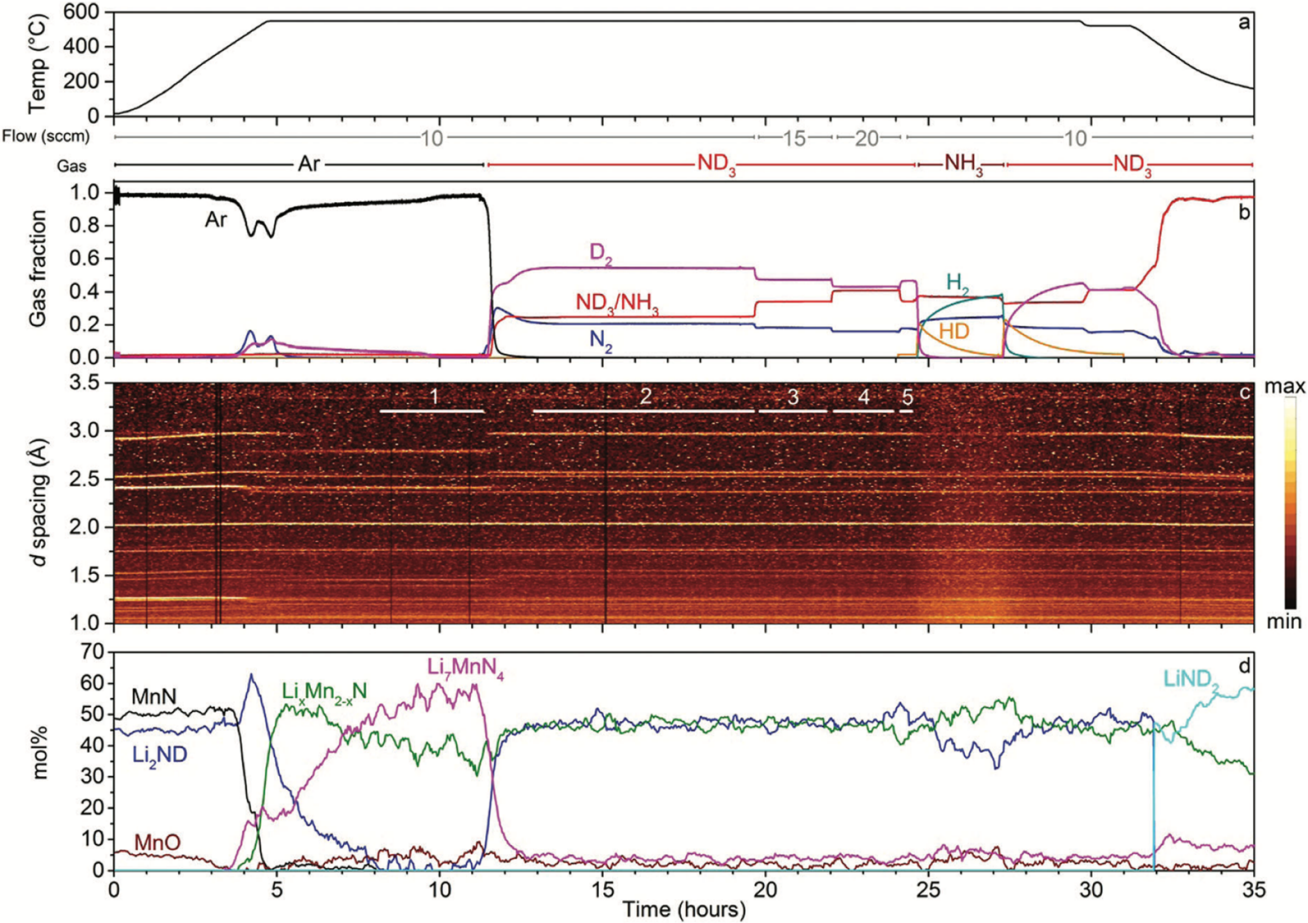
Results of the neutron powder diffraction experiment on lithium imide–manganese nitride. The panels show (a) the temperature of the sample and gas flow rate and composition, (b) the molar gas fractions of the various gas species monitored in the experiment, (c) a contour plot of the neutron powder diffraction with regions used for the analysis of summed diffraction data indicated with numbered, white lines, and (d) the molar composition of the sample obtained from Rietveld analysis of the diffraction data. Reproduced with permission from ref (362). Copyright 2018 Royal Society of Chemistry.

In exploring effective catalysts, it was found that the mixture of lithium imide and transition metals/metal nitrides (e.g., Li_2_NH-MnN, Li_2_NH-Fe_*x*_N) exhibited higher activity than Ru catalysts for ammonia decomposition. In addition, the formation/decomposition of ternary nitrides (e.g., Li_7_MnN_4_) was proposed to account for the activity.^359−362^ The bulk phase behavior of Li_2_NH-MnN and Li_2_NH-Fe_*x*_N was investigated by *in situ* neutron and X-ray powder diffraction (Figure 53).^362^ Under NH_3_ decomposition conditions, only Li_2_NH and metallic Fe, rather than Fe_*x*_N, were observed in the bulk phase of the Li_2_NH-Fe_*x*_N catalyst, due to the denitriding of the Fe_3–*x*_N in the temperature range 400–500 °C.^363^ For the Li_2_NH-MnN system, Li_2_NH and MnN were detected at 500 °C while Li_2_NH and ternary nitrides (Li_*x*_Mn_2–*x*_N and a small proportion of Li_7_MnN_4_) existed at 550 °C. However, the Li_2_NH-MnN catalyst still exhibited significant activity for NH_3_ decomposition without the presence of bulk ternary nitride phases. Thus, a bulk ternary nitride might not be required for NH_3_ decomposition over lithium imide–transition metal catalysts.

#### 4.3.2 Methanol Synthesis from CO_2_/CO Hydrogenation

**Figure 54:**
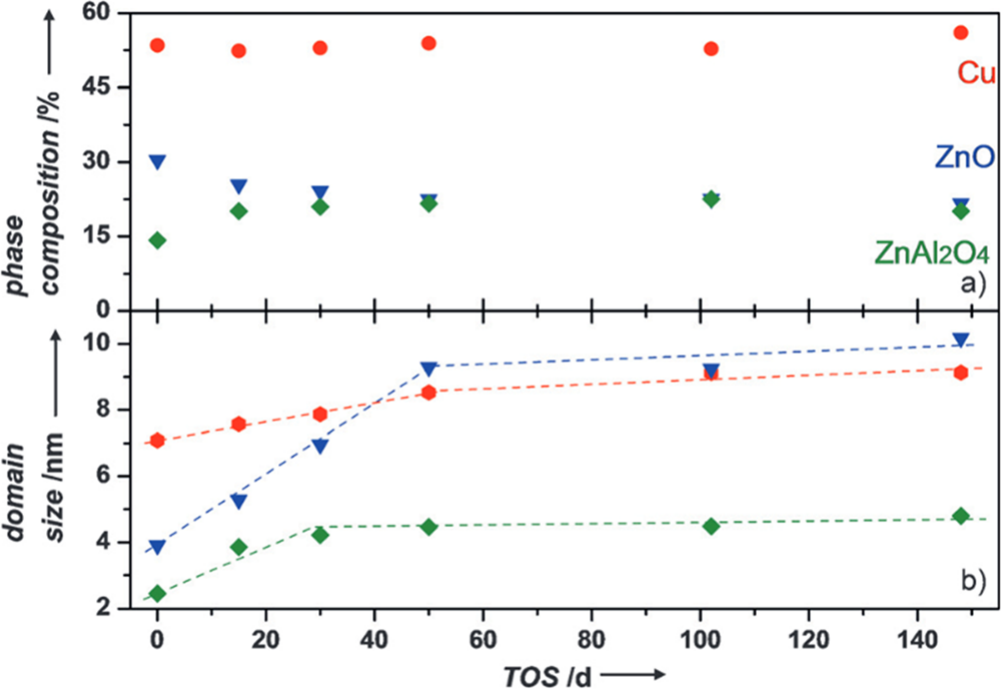
Quantitative analysis of the ND data. a) Phase composition of Cu, ZnO, and Zn, Al-spinel of the catalysts over different TOS durations. The data were obtained by Rietveld’s refinement of the ND patterns. b) Volume weighted mean domain sizes (Lvol@IB) of the corresponding phases, calculated from the width of the reflections. Key: Cu NPs (red), ZnO (blue), Zn, Al-spinel(green). Reproduced with permission from ref (337). Copyright 2016 Wiley-VCH.

Lunkenbein et al. applied ND to study the phase and size changes of a Cu/ZnO/Al_2_O_3_ catalyst for methanol synthesis over different time on stream (TOS) under industrially relevant conditions (60 bar, 230 °C, syngas (8% CO_2_/6% CO/59% H_2_/27% inert)).^337^ As shown in Figure 54a, three Cu and Zn phases were detected at the beginning of the reaction: metallic Cu, ZnO, and a Zn, Al-spinel phase. With increasing reaction time, the phase composition of metallic Cu remained almost unchanged at 50%. The concentration of the Zn, Al-spinel phase increased from 14% to 21%, and the concentration of the ZnO phase decreased from 30% to 23% over 50 days of reaction, after which the concentration of the Zn-containing phases barely changed. The domain size of the Cu- and Zn-containing phases was also tracked with the reaction time, and differences could be observed. The domain size change occurred within 50 days of reaction for all three phases. The metallic Cu only experienced weak sintering, as evidenced by the small increase in the particle size (7.1 ± 0.2 to 9.1 ± 0.3 nm) for 148 days of time on stream whereas the domain size of the Zn, Al-spinel phase increased from 2.4 ± 0.5 to 4.2 ± 0.6 nm for the first 30 days of TOS. A significant change was observed in ZnO: the coherent scattering domain size of ZnO increased from 3.9 ± 0.7 to 9.3 ± 1.3 nm in the first 50 days of TOS. These results suggested that the ZnO phase was quite dynamic under reaction conditions. It could react with Al-oxide species forming the Zn, Al-spinel phase and aggregate into large particles. Such a dynamic change of the Zn-containing phases, combined with the time-dependent change of methanol production, was suggested to be the reason for the deactivation of the catalyst.

**Figure 55:**
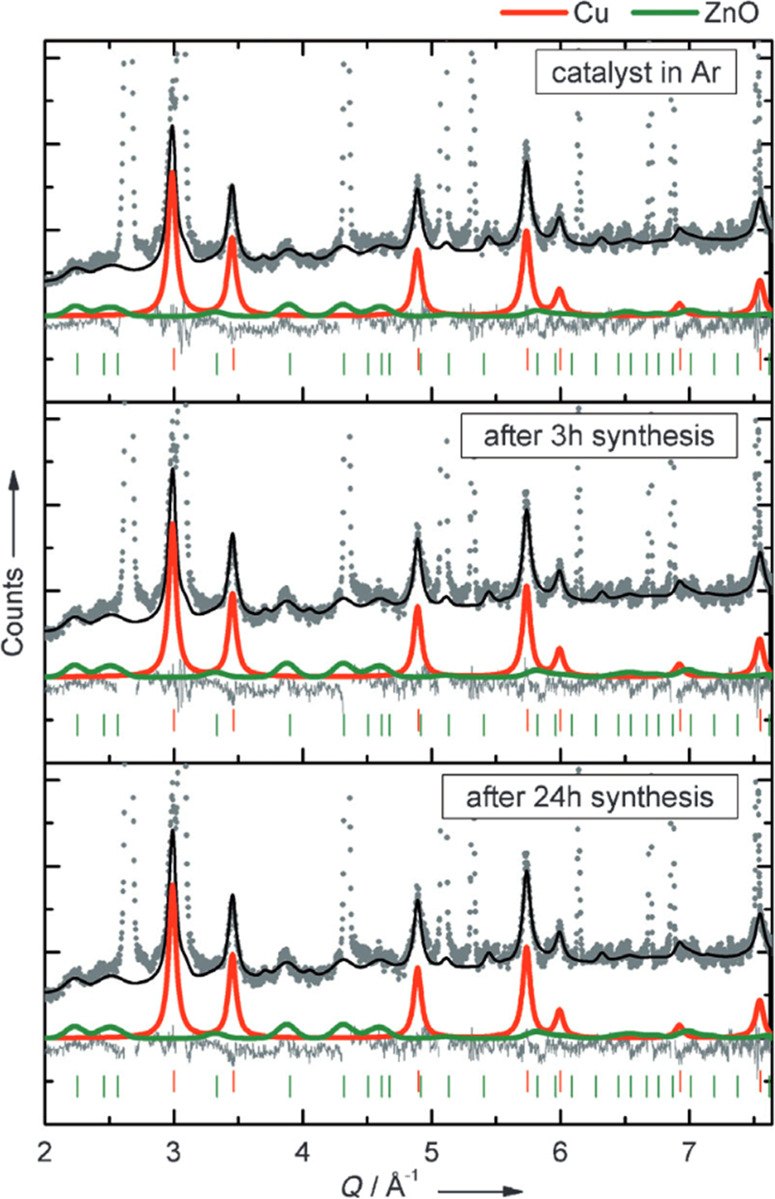
Rietveld fits of the catalyst before (0.1 MPa Ar, upper panel), at the beginning (center), and after 24 h of methanol synthesis (bottom) at 523 K and 6 MPa. Experimental data is shown in gray, and the calculated pattern of the catalyst as a black line. The thin gray line is the difference between the experimental and calculated patterns. The contribution of the Cu phase and ZnO is marked as red and green lines with tick marks at the positions of Bragg reflections. Additional strong peaks from the Al reactor wall were treated as peak-phase during Rietveld analysis and are excluded from the overall calculated profile shown here. Reproduced with permission from ref (287). Copyright 2013 Wiley-VCH.

It is generally agreed that the Cu–ZnO interface plays an important role in improving methanol selectivity. With the dynamic change of ZnO (as discussed above), the surface of the Cu phase should also change due to Cu–ZnO interaction. By applying ND, Kandemir et al. also found that metallic Cu was the major phase in the Cu/ZnO/Al_2_O_3_ catalyst, and with the increase of reaction time (over 24 h), the particle size of Cu slightly increased from 5.9 ± 0.1 to 6.4 ± 0.1 nm (Figure 55).^287^ In addition, stacking faults were observed in the Cu phase as evidenced by the shift of the (111), (200), (222), and (400) Cu peaks from *ex situ* ND data.^364^ However, the microstructure of the Cu phase with defects was relatively stable under working conditions. Stacking fault annealing and brass formation were only observed at temperatures higher than those used in the methanol synthesis process.

### 4.4 NPDF of Adsorbates and Reactions

Total scattering refers to the measurement and analysis of the complete diffraction pattern, including Bragg and diffuse components.^313^ This is an approach frequently applied to systems that exhibit short-range order, such as amorphous glasses, liquids, or highly disordered crystals.^365,366^ The sensitivity of neutron scattering to light elements, particularly ^1^H and ^2^H, means that the technique is well-suited to studies of catalysts. However, it has been relatively little exploited for this purpose to date. One example is the use of the technique to directly measure metal–hydrogen distances for hydrogen adsorbed on Raney nickel and on a supported platinum catalyst.^117^

**Figure 56:**
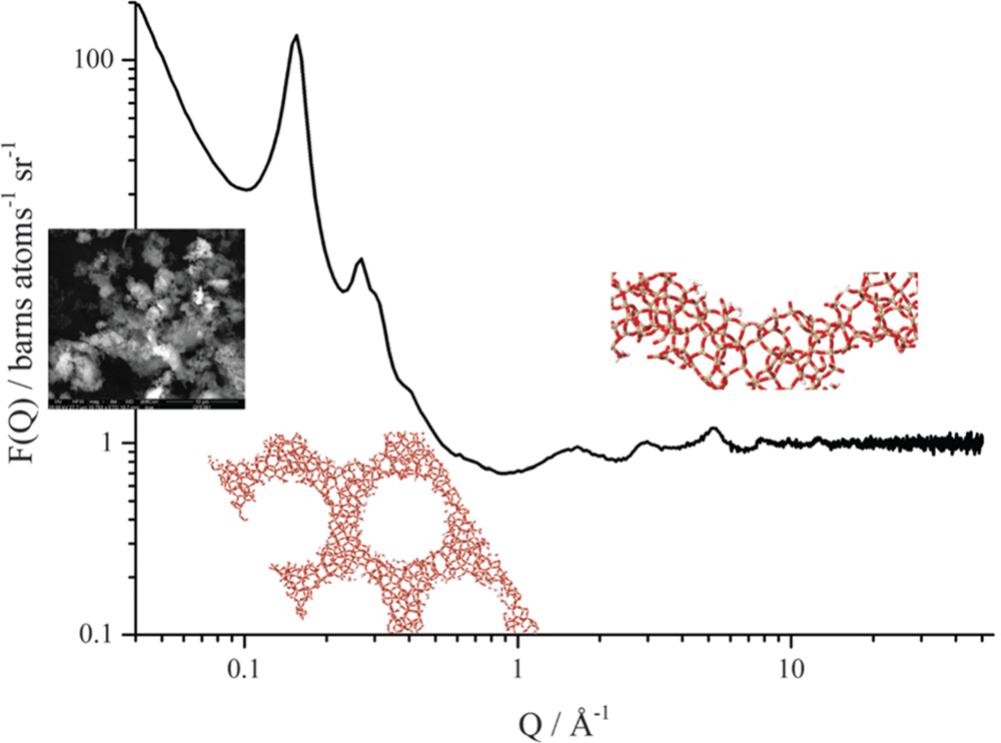
Total neutron scattering structure factor obtained for evacuated MCM-41 on NIMROD. In a single measurement, information about the size and shape of sample’s grains can be obtained (low *Q*-range), as well as unit cell size from Bragg scattering features and interatomic correlations within the sample, such as average distances between silicon and oxygen in MCM-41 (high *Q*-range). Reproduced with permission from ref (23). Copyright 2016 Royal Society of Chemistry.

A recent application is the study of the platinum catalyzed hydrogenation of aromatic molecules in MCM-41. Figure 56 shows the total scattering structure factor of evacuated 3 wt % Pt/MCM-41.^23^ The material comprises aggregates of grains that give rise to small angle (Porod) scattering at small *Q* values, columnar pores of ∼33 Å that give the sharp peaks at 0.1–1 Å^–1^ and the usual tetrahedral silica structure that gives the peaks at 1–10 Å^–1^. This illustrates the technique’s power: in a single measurement, information at the microscopic, mesoscopic, and macroscopic length scales can be obtained.

**Figure 57:**
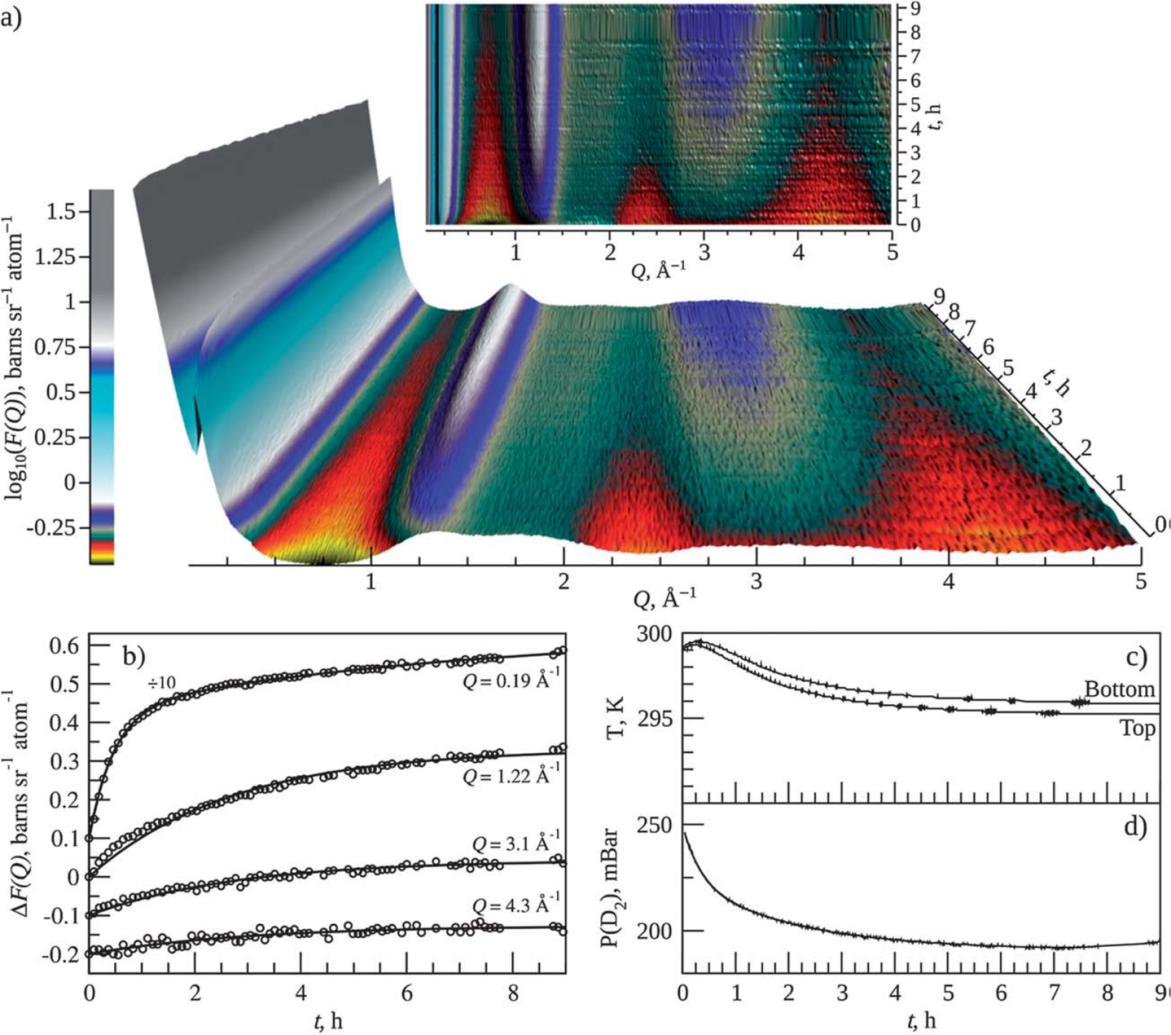
(a) *F*(*Q*)/time domain data over the course of the reaction. (b) Slices taken through (a) at specific *Q* values, and the corresponding exponential fits to the data. Curves are offset vertically for clarity. (c) Sample temperature as measured at the top and bottom extremes of the TiZr cell. (d) Pressure of D_2_ gas present in 4 L supply reservoir. Reproduced with permission from ref (24). Copyright 2013 Royal Society of Chemistry.

Introduction of deuterated benzene^24^ or deuterated toluene^23^ by capillary condensation followed by exposure to D_2_ results in the catalyzed hydrogenation of the aromatic to perdeutero cyclohexane and perdeutero methylcyclohexane, respectively. The reaction is relatively slow at room temperature, so it can be followed in real time, as shown in Figure 57a for benzene hydrogenation. By taking cuts at particular *Q* values with respect to time, the kinetics corresponding to different length scales in the system can be determined, Figure 57b.

The first diffraction peak (*Q* = 0.19 Å^–1^) exhibits both a fast (rate constant *k*_0_ = 2.146 h^–1^) and a slow (*k*_1_ = 0.138 h^–1^) component, while at *Q* = 1.2, 3.1, and 4.3 Å^–1^ there is an intermediate rate (*k*_2,3,4_ ≈ 0.35 h^–1^). The fast process (*k*_0_) is attributed to the dissociative adsorption of D_2_. This is consistent with the rapid and relatively large increase in temperature over the first 30 min following the introduction of the D_2_, Figure. 57c. The slow component (*k*_1_) also does not reflect a chemical change and is likely related to pore diffusion. The change at *Q* = 1.22 Å^–1^ reflects nearest-neighbor molecular interactions, while those at *Q* = 3.1 and 4.3 Å^–1^ are associated with atomistic chemical changes within the system. These three features evolve with similar time constants and are correlated with the reduction process and the formation of the cyclohexane product. These time constants suggest that the overall process is likely to be limited by liquid diffusion (*k*_1_), as this is the slowest rate observed, while the overall reaction rate is governed by the hydrogenation process (*k*_2,3,4_) rather than the dissociation of D_2_ (*k*_0_).

While the rate constants can be extracted from the data by conventional analysis of the kinetics, understanding what gives rise to these, particularly the liquid structure in a confined volume, is much more complex. Neutron total scattering is sensitive to these effects, and the liquid structure, both confined and in the bulk, has been determined by Empirical Potential Structure Refinement (EPSR).^367^ This is a Monte Carlo simulation of a representative box of molecules (hundreds to thousands) that uses a reference interatomic potential. A comparison between the experimental and calculated (from the model) structure factors is used to construct a perturbing potential (the empirical potential) that is applied to the Monte Carlo simulation. This ensures that the final model will reproduce the experimental data. This then allows properties of interest, such as site–site radial distribution functions, angular distribution functions and spatial probability densities to be extracted from the model.^368^ To provide additional constraints to the model, an NMR capability has been added to the experimental setup.^369,370^

The diffusion of benzene in MCM-41 has been investigated by QENS and molecular dynamics (MD) and is much slower than in the bulk.^371^ The MD model is largely in agreement with that obtained by EPSR;^372^ both show concentric layers in the MCM-41 pores. Notably, the nanoscale confinement of the liquid has a major effect on the spatial and orientational correlations observed between the molecules, when compared with the structure of the bulk liquid.^373^

The sensitivity of neutron scattering to light elements has been used to characterize other materials^374,375^ (N_2_, O_2_, D_2_, CD_4_) in MCM-41. Reactions involving these species have yet to be studied, but there is obvious potential to do so.

## 5 Other Neutron Scattering Techniques for Catalysis Research

### 5.1 Introduction to Quasielastic Neutron Scattering (QENS)

As with any INS measurement, a QENS experiment is concerned with obtaining the *S*(*Q*,*E*) as a function of momentum (*Q*) and energy transfer (*E*). However, QENS is specifically aimed at measuring the thermally activated stochastic motions. Therefore, unlike vibrational spectroscopy, which is best performed at the lowest practically attainable temperature, QENS is employed at finite temperatures (which could include ambient and higher temperatures). QENS measurements present especially stringent requirements to the energy resolution of the neutron spectrometer because the characteristic energy scale of the stochastic processes lies well below those of the intra- and even intermolecular vibrational motions. In other words, QENS is concerned with the dynamics on a much longer time scale than those probed in a vibrational INS measurement. The characteristic microscopic time associated with stochastic processes, τ, follows an Arrhenius law, τ(*T*) = τ_0_exp(*E*_a_/*T*), with an activation energy *E*_a_ and a prefactor τ_0_. The activation energy may be temperature-dependent, *E*_a_(*T*), leading to the non-Arrhenius temperature dependence of the τ(*T*).

Thus, a better energy resolution of the spectrometer allows measurements of the stochastic dynamics at lower temperatures. At the same time, access to higher energy transfers enables measurements of the stochastic dynamics over a broad temperature range. High energy-resolution backscattering neutron spectrometers, widely employed for QENS measurements, suffer from the limited range of accessible energy transfers. In contrast, broadband vibrational spectrometers do not have sufficient energy resolution for QENS measurements. The new spectrometer design for simultaneous QENS and vibrational INS studies will address this long-standing instrumentation challenge.^376^

In most QENS experiments, the scattering signal is dominated by the hydrogen atoms in the sample, as the neutron scattering cross section of protons is large compared to other elements. Besides, this cross section is predominantly incoherent. Thus, QENS probes single-particle (not collective) microscopic dynamics. For example, commonly encountered continuous (Fickian) diffusion gives rise to the QENS signal, which is a Lorentz function in energy, *S*(*Q*,*E*) = Γ(*Q*)/(π(Γ(*Q*)^2^ + *E*^2^)), with a half-width at half-maximum (HWHM) Γ(*Q*) = η*DQ*^2^, where *D* is the diffusion coefficient and η is the reduced Planck’s constant. Therefore, the diffusion coefficient can be directly determined from the slope of the Γ(*Q*) plotted as a function of *Q*^2^.

Another commonly encountered case is jump diffusion, where instead of moving continuously, a particle resides for a specific time, τ, between successive jumps. For jump diffusion, HWHM is often described by a Chudley-Elliot model: Γ(*Q*) = η*DQ*^2^/(1 + *τDQ*^2^), which is reduced to the continuous diffusion model when τ = 0. Because of the *D* = *L*^2^/(6τ) relationship between the diffusion coefficient, the residence time between jumps, and the jump length, *L*, the Γ(*Q*) can be expressed as a function of two different variables: (τ,*D*), (τ,*L*), or (*D*,*L*). Besides, for spatially constrained diffusion, or localized jumps the Γ(*Q*) can present as a *Q*-independent variable over a specific *Q* range.^25^

Often, the *S*(*Q*,*E*) is not described by a Lorentz function, especially for heterogeneous systems, but a “stretched” function. A numerical Fourier transformation of a stretched exponential, exp[−(*t*/τ(*Q*))^β(*Q*)^], which becomes a Lorentz function in the limiting case of β = 1, is commonly used for a “stretched” *S*(*Q*,*E*). Alternatively, the Cole–Cole relaxation function could describe a “stretched” *S*(*Q*,*E*) in an analytical form in the energy space.^377^ In the limiting case when the “stretching” parameter is vanishingly small, the Cole–Cole expression is also reduced to a Lorentz function. Besides being analytical in the energy space, another advantage of the Cole–Cole expression for *S*(*Q*,*E*) is that its average relaxation time does not depend on the value of the “stretching” parameter. In contrast, for a Fourier-transformed stretched exponential the average relaxation time depends on both τ and β, which increases the uncertainty in the calculated average relaxation time.

#### 5.1.1 QENS Instrumentation

QENS is a technique that uses very low energy neutrons to look at energy transfers in the range ±10 meV (±80 cm^–1^) and generally much smaller than this. The energy resolution of a spectrometer, Δ*E*, and the time scale τ of the motion are related by the Heisenberg uncertainty principle: Δ*E*τ ∼ ℏ, thus rapid motions require relaxed resolution, while slower motions require high resolution. The time scale to be probed can be separated into three regimes, each of which uses a different technique: for τ ∼ 10^–11^ s, Δ*E* is 10–100 μeV and direct geometry time-of-flight is used; for τ ∼ 10^–9^ s, Δ*E* is 0.3–20 μeV and backscattering crystal analyzer is used; for τ ∼ 10^–7^ s (and slower), Δ*E* is 0.005–1 μeV and neutron spin echo is used.^300^

The need for low energy neutrons means that this is an area where reactor sources excel, especially the ILL, as they have greater fluxes at these energies than spallation sources. However, spallation sources typically have much lower backgrounds than reactors and pulsed sources are particularly suited to ToF instruments. As a result, QENS is becoming an increasingly important part of the instrumentation suite at spallation sources.

ToF QENS spectrometers are conceptually similar to those used for vibrational spectroscopy (see Section 3.1) but require more sophisticated chopper systems to achieve the resolution needed. Examples used for studies of catalysis include IN5 at the ILL LET at ISIS and CNCS at SNS.^378−380^

The design of backscattering QENS spectrometers varies between reactors and spallation sources. At reactors, an incident wavelength is selected by a monochromator. The bandwidth is increased by either moving the monochromator to give a Doppler broadening to the incident beam or heating/cooling the monochromator crystal so as to change the lattice spacing and hence the selected wavelength. This results in high resolution (better than 1 μeV) but very restricted energy transfer range. A recent development (BATS on IN16B at the ILL) has extended the energy transfer range, albeit at the cost of flux.^381,382^ At spallation sources the instruments are indirect geometry spectrometers: a (relatively) broad band of energies is selected by a chopper system and the analysis is by Bragg reflection from a crystal, typically graphite (002)/(004) or Si(111)/(311). The higher order indices result in a wider energy transfer range but with lower resolution. Backscattering instruments at spallation sources (BASIS at SNS and OSIRIS at ISIS) are those that are most commonly used for catalyst studies.^382,383^

For the very slowest motions spin echo instruments are used. Here, the velocities of a beam of polarized neutrons incident on, and scattered from, a sample are coded as the number of Larmor precessions that the neutron spins undergo in a well-defined applied magnetic field. If the scattering at the sample is elastic and the precession fields before and after the sample are identical in magnitude and spatial extent, all neutrons will be in phase at the point in space where they leave the second precession region, the “echo”. If the scattering is not purely elastic, the spins will not be completely in phase, weakening the echo. The distribution of phases is then a measure of the distribution of the atomic velocities in the sample. The high resolution of this technique arises from the large number of precessions the neutron undergoes, ∼10^5^ precessions/m, hence the velocity can be measured to an accuracy of better than 10^–5^ consequently the energy transfer can be measured to similar accuracy. The technique is slow compared to direct geometry and backscattering instruments because the flux is low as a result of the need to polarize the neutrons. To date, it has been little used for catalysis studies, although it has considerable promise for the study of complex molecules in confinement, as shown by a study of isobutane in silicalite.^384^

The sample environment for QENS is similar to that used for other neutron techniques, e.g., INS and neutron powder diffraction. There are two key requirements: (i) as QENS measurements are routinely made as a function of temperature, the sample can must be suitable for the temperature range, and (ii) QENS is a *Q*-resolved technique. Thus, it is essential to avoid multiple scattering that will destroy the *Q* information. Annular aluminum cans are usually employed for samples that can be prepared off-line and are stable. The thickness of the annulus is chosen such that the sample scatters, at most, 10% of the incident neutrons. (This level of scattering means that multiple scattering is negligible). Air sensitive samples can be loaded into the cans in a glovebox and are usually sealed with indium wire. The indium seal limits the maximum temperature to ∼100 °C (mp In = 157 °C). For measurements where the sample needs to be treated *in situ* (e.g., diffusion of a gas through a zeolite), versions of the cells are available that allow gas loading either statically (by exposure to a gas reservoir) or under flow. For measurements above 100 °C, Cu gasket (Conflat) sealed steel or niobium cells are used. The disadvantage of these materials is that they introduce a significant number of Bragg peaks into the data, which reduces the coverage in *Q* and complicates the analysis.

#### 5.1.2 The Use of QENS in Catalysis

**Figure 58:**
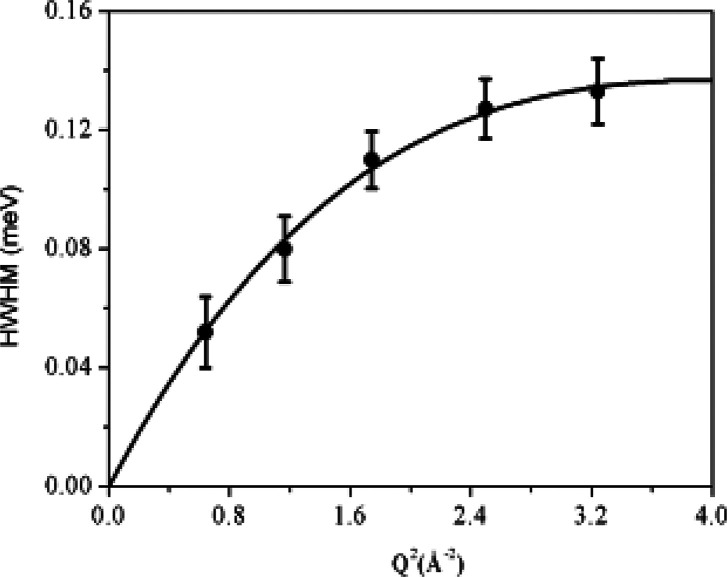
Variation of the half-width half-maximum as a function of *Q*^2^ for methanol adsorbed in MCM-41. The solid line is the fit to a Chudley-Elliot jump diffusion model. Reproduced with permission from ref (397). Copyright 2006 American Chemical Society.

Even though QENS measurements are commonly used for probing the geometry of the local atomic or molecular jumps, it is the translational (albeit suppressed by confinement) diffusivity that has been of prime interest in QENS studies of catalysis, with only a few exceptions^385−388^ concerned with rotational molecular motions. This is because the mass transport of reactants or products linked to translational diffusivity could be an essential step in a chemical process. Historically, most QENS studies of catalytically active materials have been concerned with the mobility of hydrocarbons in zeolites. Accordingly, several reviews in the existing literature focused on QENS application in catalytic science^29,389,390^ with an emphasis on zeolites and related materials. Early QENS measurements of catalytic materials involved studies of diffusion of methane,^391^ ethane and propane,^392^ and isobutane^393^ in ZSM-5 zeolite. Comparative studies of diffusion of longer *n*-alkanes in Na-ZSM and silicalite^394,395^ were carried out shortly afterward. A detailed comparison of diffusivities of *n*-alkanes in these MFI-type zeolites measured by QENS and NMR was subsequently presented.^396^ A comparison of methanol adsorbed in HZSM-5 and a mesoporous MCM-41 silica^397^ revealed much more restricted dynamics of methanol in the former matrix than in the latter. Figure 58 presents the variation of the QENS signal HWHM as a function of *Q*^2^ for methanol adsorbed in MCM-41, with the solid line indicating a fit to a Chudley-Elliot jump-diffusion model.

**Figure 59:**
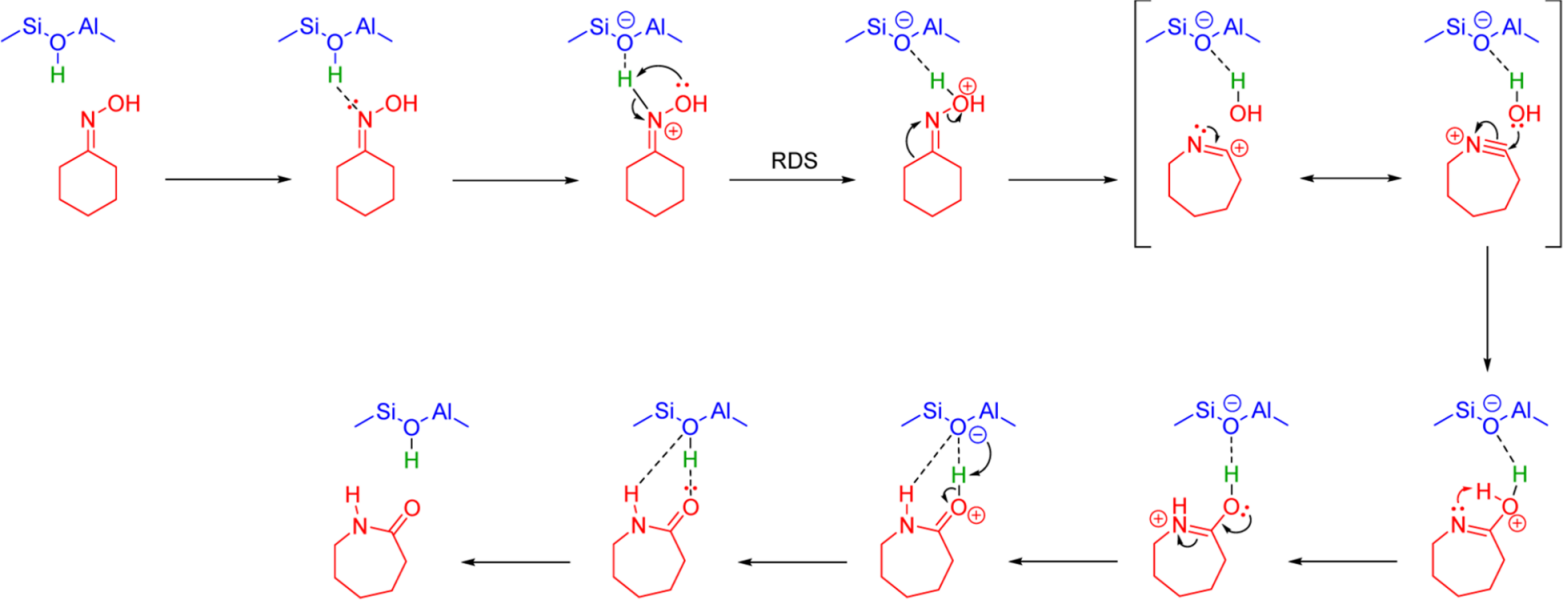
A representative mechanistic pathway for the acid-catalyzed Beckmann rearrangement of cyclohexanone oxime. Reproduced with permission from ref (268). Copyright 2017 American Chemical Society.

In general, mesoporous confinement does not impede the translational diffusivity to the extent that confinement in zeolites does. Further QENS studies of methanol mobility in H-ZSM5 and HY zeolites^263,398^ revealed immobilization of methanol in H-ZSM5, suggesting the framework methoxylation, whereas methanol in HY remained intact and mobile. The translational immobility of methanol in ZSM-5 on the time scale accessible to QENS was further confirmed in an experiment^399^ where isotropic methanol rotation was eventually observed in a sample that developed mesoporosity due to spending a long time at a high reaction temperature. The mobility of propylene in Na-ZSM5^400^ was also probed. Another early target of QENS studies was NaY zeolite material, in which diffusion of propane^401^ and acetylene^402^ was studied. These studies were followed by a detailed comparison of the mobility of propylene^403,404^ and acetylene^405^ between ZSM-5 and NaY zeolites. The systems less systematically investigated by QENS included *n*-octane in faujasite type X,^406^ ammonia diffusion in NH_3_-SCR zeolite^407^ and cyclohexanone oxime in SAPO-37 compared with Zeolite-Y and ZSM-5.^268^ In the latter case, QENS measurements provided information on the critical role of diffusion and interaction of the substrate with the desired active site. Specifically, QENS showed cyclohexanone oxime to access the internal sites of SAPO-37 and Zeolite-Y but not ZSM-5. Figure 59 presents a mechanistic pathway for the acid-catalyzed Beckmann rearrangement of cyclohexanone oxime to form ε-caprolactam, an important feedstock in the production of Nylon-6.

There are a couple of points concerning QENS studies of zeolites and related materials that are worth noting. First is the prominent role often played in such studies by MD simulations. Second are the notable recent attempts aimed at *operando* measurements, albeit at present realized only for homogeneous systems. For example, the complexation of NiCl_2_ with 2,2′-bipyridine was followed using QENS to monitor the progress of the reaction.^408^ Such QENS studies are expected to become more prevalent in the future.

Besides probing zeolites, another class of QENS studies relevant for catalytic science are measurements of dynamics of surface species, which form a distinct subfield with its own peculiarities within the extensive research on confined species. Only a few review articles have been published on surface species studied by QENS,^409^ and molecular dynamics simulations of such systems are considered more challenging, mainly due to some ambiguity in defining the two-dimensional parameters such as self-correlation functions and diffusivities.^410,411^ Following early work on the diffusion of hydrogen on the surface of nickel catalyst,^412,413^ QENS measurements of two-dimensional surface diffusion were reported for methane^414,415^ as well as butane and hexane^416^ on graphite, methane on magnesium oxide,^417^ and water on zirconium oxide.^418,419^ The QENS data analysis used in these studies had much in common with the approach utilized for two-dimensional layered materials, such as protons in (CsOH)(H_2_O)^420^ and water in clays,^421^ or the alternative approach applied to the diffusion of xenon on platinum^422^ and benzene on graphite.^423^ However, many QENS studies of surface adsorbates, e.g., gallium on alumina,^424^ water on SrF_2_ and ZnO,^425^ acetonitrile on TiO_2_,^426^ and cyclohexane and benzene on nickel,^427^ do not invoke a two-dimensional formalism. This is especially true for QENS studies of water on oxide surfaces, many of which aim at investigating not the well separated adsorbate molecules but the surface coverage levels at which the water adsorbate demonstrates some essential dynamic characteristics of bulk water, albeit without undergoing crystallization at any temperature.^428−431^ Interestingly, the dynamics of phenanthrenequinone molecules on onion-like carbon surfaces measured by QENS as a function of temperature and surface coverage^432^ bears qualitative resemblance to the dynamics of water molecules on oxide surfaces, suggesting the universality of some dynamics features across different classes of surface adsorbates. Finally, some QENS studies of surface water, particularly in minerals, attempt to analyze the dynamics of water molecules associated with specific surface atoms.^433,434^

In summary, QENS plays an increasing role in interrogating the diffusional dynamics of catalytically active reaction systems. Coincident molecular dynamics simulations are proving to be particularly insightful. Given the relevance of diffusional characteristics within confined geometries to selectivity profiles (for example, as encountered in hydrocarbon reactions in zeolite catalysts), this activity is anticipated to increase over the coming years.

### 5.2 SANS: Introduction and Its Application in Catalysis

Since Stuhrmann et al. demonstrated the feasibility of small-angle neutron scattering (SANS), it has become an important characterization method in the fields of polymer and colloid science because of the length scales it probes.^435^ Different from wide-angle diffraction methods, which probe atomic distances, SANS uses elastic neutron scattering at small scattering angles to study structures of various substances at a mesoscopic scale of 1–1000 nm. SANS is a powerful complementary method to small-angle X-ray scattering (SAXS), and both techniques share the same basic principles. Specifically, in a SANS setup, the incident neutron beam is monochromated and collimated before it hits the sample. The detector records the neutrons scattered by the sample and the nonscattered neutrons are absorbed by the beam stop in the center of the detector. Due to the differences in the physics of X-ray–matter and neutron–matter interaction, SANS has some unique and valuable features. For instance, the scattering lengths of hydrogen and deuterium have opposite signs, allowing the important technique of contrast variation/matching, which can selectively highlight different parts of samples. In addition, due to the strong scattering by magnetic moments, magnetic SANS has played a vital role in the fields of nanomagnetism.

**Figure 60:**
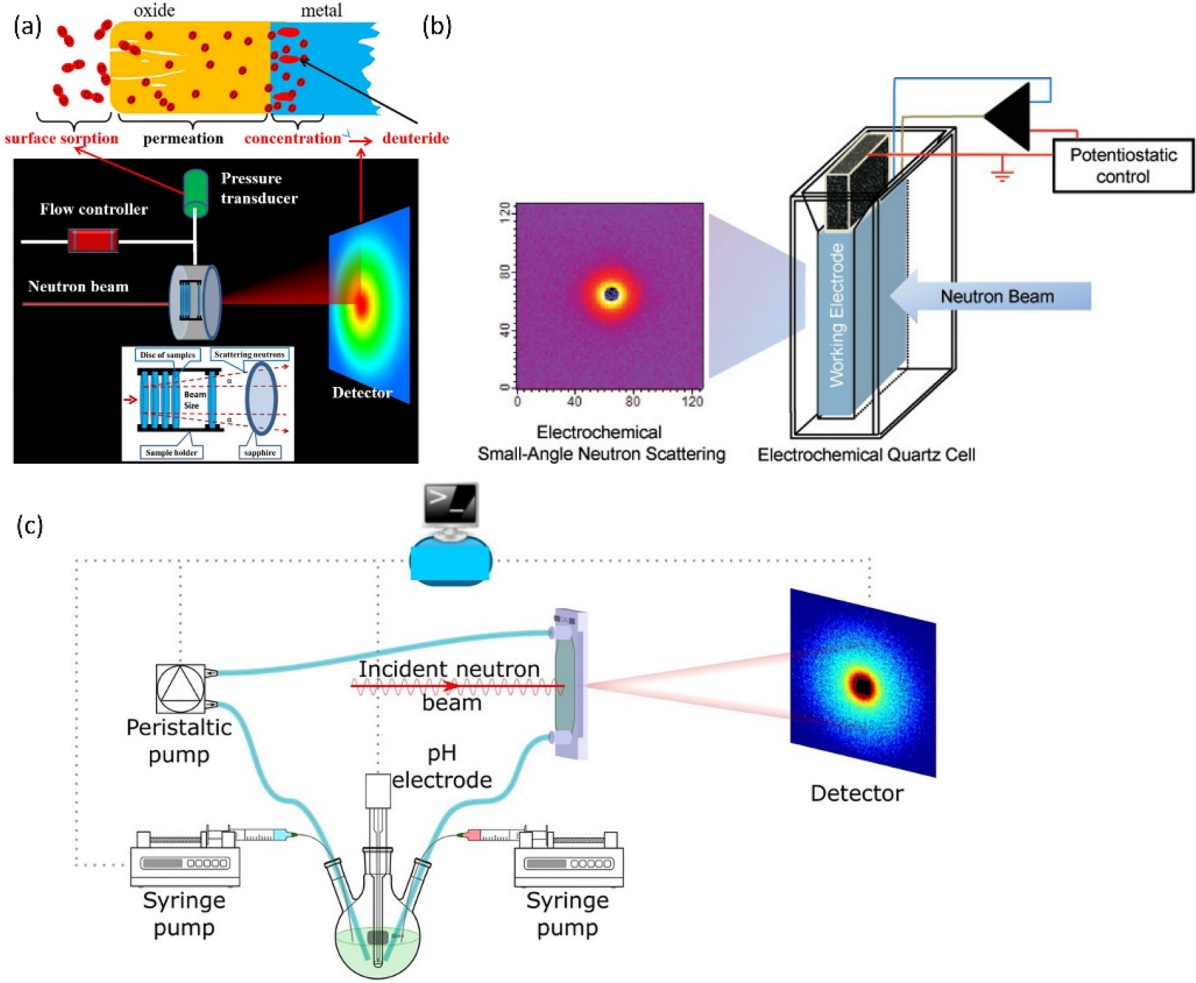
(a) A schematic diagram of the *in situ* SANS experiment of the deuterium-cerium reaction. Reproduced with permission from ref (436). Copyright 2020 American Chemical Society. The two ends of the reactor are sapphire windows with a neutron transmission of 95%. The 316L stainless steel cylinder is connected to the gas inlet/outlet. A multiplate 316L stainless steel sample holder is mounted in the middle of the reactor. Inset: Using the multiplate sample holder, the deuterium reaction of multiple wafers can be realized. Reproduced with permission from ref (437). Copyright 2021 Elsevier. (b) A schematic of the transmission SANS quartz cell with working electrode under potentiostatic control.^438,439^ Reproduced with permission from ref (438). Copyright 2012 American Chemical Society. (c) A schematic of a continuous flow setup. The experimental setup includes a reaction vessel, a pH electrode, a peristaltic pump, an observation cell, and two syringe pumps. Reproduced with permission from ref (440). Copyright 2018 Nature Publishing Group.

With the development of *in situ* setups, *in situ* SANS has been used to investigate several different systems, as demonstrated in Figure 60. By using *in situ* SANS and a custom reaction chamber (Figure 60a), Yan et al. studied deuteride formation on the surface of cerium (coated with a surface oxide layer).^436,437^ It was found that deuterides were formed as soon as deuterium was absorbed on the sample with a thin oxide layer, while for the sample with a thick oxide layer, the precipitation of deuterides was delayed.^436^ Prabhu et al. developed an *in situ* electrochemical SANS methodology, which enabled direct measurements of nanomaterial dispersion structure under redox reactions at the vitreous carbon electrode (Figure 60b).^438,439^ A feasibility test was performed on ZnO nanoparticles in 50 mmol/L NaCl deuterium oxide solution under bulk electrolysis at negative potentials.^438,439^ The *in situ* SANS results showed irreversible nanoparticle structural changes during the potential cycle.^438,439^ To follow the structural changes under continuous chemical reactions, Hayward et al. proposed to use the setup shown in Figure 60c for SANS experiments.^440^ The approach was validated by performing single and multiple potentiometric titrations on an aqueous anionic surfactant solution (oligo-oxyethylene alkylether carboxylic acid in D_2_O) with addition times varying from 1 s to 2 h.^440^

SANS has been used in the field of catalysis to provide information about the particle size and shape of catalytic systems. For instance, Larichev et al. showed the possibility of using SANS to determine the particle size of the active component in catalysts. They demonstrated that the combination of SANS and SAXS made it possible to obtain the distributions of particles of the deposited active component in a wide range of sizes.^441^ By employing SANS, Kim et al. were able to measure the structure of the γ-Al_2_O_3_ supports over four orders of length scales (from nanometer to micrometer) and showed that the catalytic activities and dispersion of Pt particles on the γ-Al_2_O_3_ support were dependent on the shape of the support.^442^ By using SANS, Acharya et al. found that the coke (a monolayer with an average composition of CH_0.3_) preferred to coat the 3.3 nm diameter capillary solid structures in a silica–alumina catalyst.^443^

Taking advantage of its sensitivity to pore filling, SANS has been utilized to study liquids (including water) and ions confined inside porous materials such as electrocatalysts. Boukhalfa et al. demonstrated the unique ability of SANS to monitor organic electrolyte ion adsorption in carbon pores as a function of the applied potential and pore size.^444,445^ The *in situ* SANS measurements revealed that ion adsorption was strongly enhanced in the smallest subnanometer pores, despite their incomplete wetting by the electrolyte solvent.^444,445^ Based on SANS results, Rother et al. were able to model the spatial distribution of confined supercritical CO_2_ in nanoporous silica aerogel.^446^ Chathoth et al. correlated the pore size distribution in nanoporous carbon aerogel with methane diffusion.^447^ The combined quasi-elastic and small-angle neutron scattering showed that at low pressures (≤2.76 MPa), the methane adsorption process began in small, subnanometer pores and moved progressively to bigger pores, leading to the initially increased diffusion coefficient with pressure.^447^ At higher pressures (>2.76 MPa), the effect of intermolecular collisions became important, resulting in a decrease in the diffusion coefficient.^447^ To develop automotive exhaust purification systems, Yoshimune et al. studied the evaporation process of immersed water from exhaust gas catalysts by applying *in situ* SANS.^448^ The time-resolved measurements showed that water started to evaporate from the secondary pores of Al_2_O_3_ supports in tens of seconds and subsequently from the primary pores in a hundred seconds and revealed that the drying rate depended on the secondary pore size of porous Al_2_O_3_.^448^

**Figure 61:**
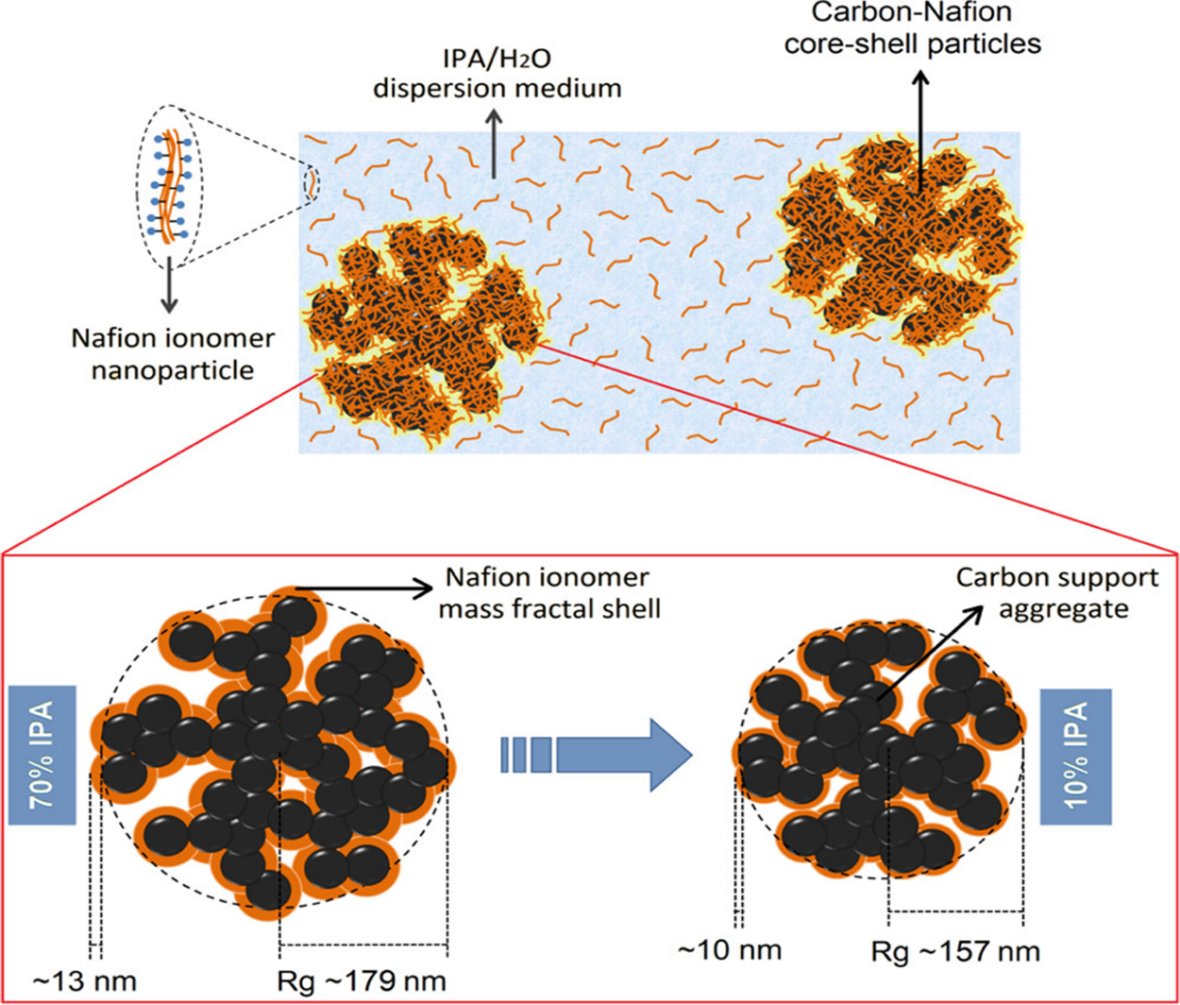
Schematic illustration of the colloidal structure of fuel cell catalyst ink prepared using IPA/H_2_O mixtures as DM. Reproduced with permission from ref (450). Copyright 2019 American Chemical Society.

The contrast-variation/matching capability of neutron scattering is particularly advantageous for studying catalytic reactions involving the interaction between catalysts and water. For instance, to overcome the diffusion loss in polymer electrolyte fuel cells that could occur when generated water overflows and hinders oxygen gas diffusion at the surface of the cathode catalyst Pt, Koizumi et al. investigated the microstructure and water distribution in carbon supported Pt catalysts by combining SANS and scanning electron microscopy (SEM).^449^ It was found that the Nafion ionomer over the carbon-supported Pt absorbed water at the 17 wt % level. The catalyst bound by the ionomer showed water repellence, whereas the catalyst powder without the ionomer was covered with water.^449^ To understand the influence of the quality of the dispersion medium on the hierarchical structure of the electrocatalysts layer of the proton-exchange membrane fuel cell, Balu et al. systematically investigated the effects of reducing the alcohol content in isopropyl alcohol/water (IPA/H_2_O) binary mixtures as dispersion medium (DM) on the structural evolution of water-rich catalyst ink using contrast-variation small angle and ultrasmall-angle neutron scattering techniques.^450^ The catalyst ink prepared using 70% IPA was shown to consist of randomly distributed globular carbon aggregates (mean radius of gyration of ∼178.9 nm) stabilized by an ionomer mass fractal shell (thickness of ∼13.0 nm), which was dispersed in the matrix of rodlike (∼1.3 nm radius and ∼35.0 nm length) negatively surface-charged ionomer NPs (as shown in Figure 61). While for DM formulations of lower IPA content, there was an increase in the ionomer NP radius and electrostatic repulsion and a decrease in the carbon aggregate size and ionomer shell thickness of the catalyst ink. The results suggested that adjustments of the DM composition could be used as a controlling parameter to tailor the hierarchical structure of the colloidal fuel cell catalyst ink and to further optimize the performance of the electrocatalysts layer.^450^

In brief, SANS has unique capabilities in determining the size and shape distribution of active metal species in supported catalysts, the formation and location of coking, and the adsorption, diffusion and confinement of ions and molecules in porous structures. With the development of *in situ* setups, chemical processes can be tracked *in situ* or by performing time-resolved measurements.^451^ In particular, using contrast variation SANS, different parts of a sample can be selectively highlighted by exchanging hydrogen for deuterium, which is especially useful for studying catalytic reactions that involve hydrogen species.

### 5.3 Neutron Imaging of Catalysts

**Figure 62:**
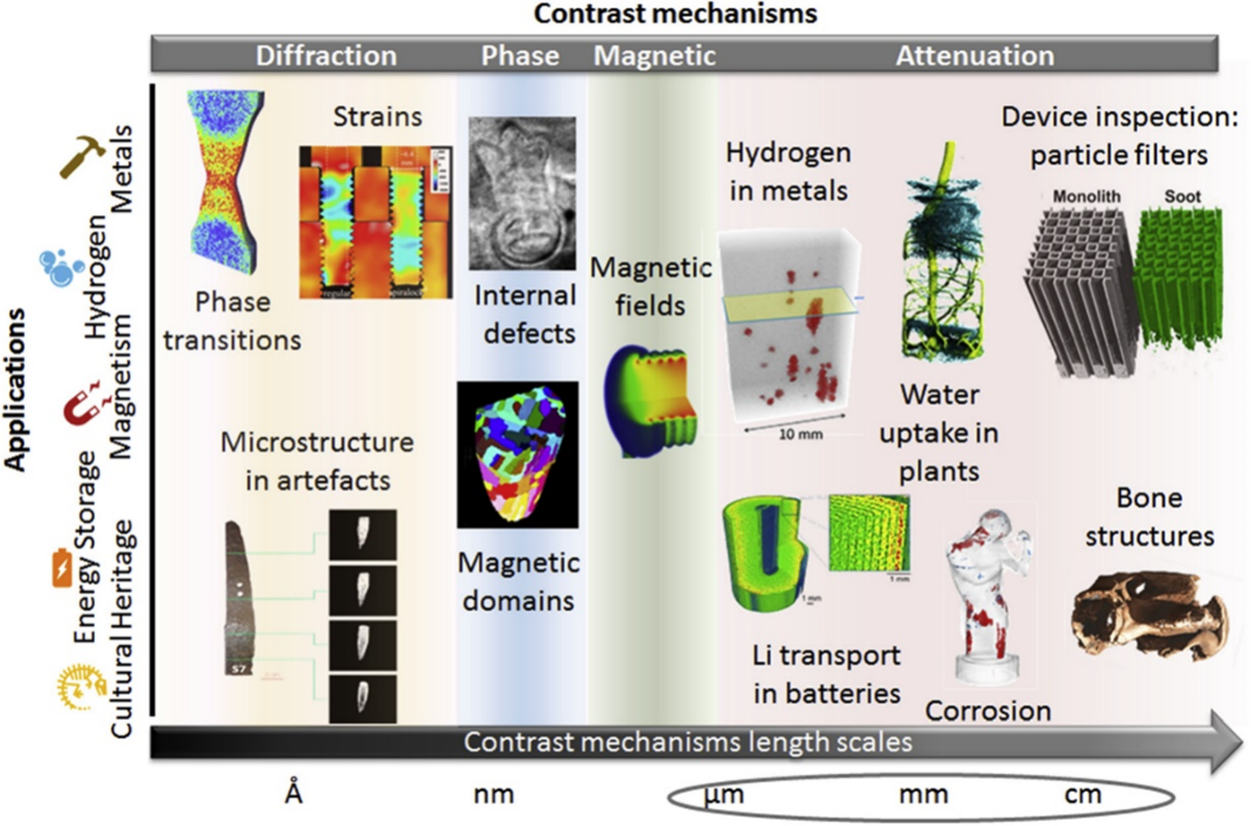
Different contrast mechanisms can be used to explore various length scales in materials and to study their properties and related processes. The relation between contrast mechanisms and different application fields is presented. The length scale shown on the lower axis relates to the corresponding contrast mechanism specified on the upper axis. For the attenuation-based image techniques the large length scale was emphasized by grouping the scales from μm to cm. Reproduced with permission from ref (455). Copyright 2018 Elsevier.

Neutron imaging has a long history,^452^ the first experiments were carried out in the 1930s, shortly after the discovery of the neutron by Chadwick in 1932. The current status is that all of the major neutron scattering facilities either already have or will have an imaging capability.^453^ The interest in the method arises from the range of contrast mechanisms, and hence the wide applicability of the technique,^454−456^ see Figure 62.

The spatial and temporal resolution are inversely related. State-of-the-art instruments can achieve ∼50 μm spatial resolution with an acquisition time of minutes,^455,457−459^ 5–10 μm resolution is possible with a limited field of view.^459,460^ High-speed imaging with 10–1000 ms time resolution with 200–500 μm spatial resolution is also possible.^461^

The major use of neutron imaging in catalysis-related research has been the study of water management in proton-exchange membrane fuel cells (PEM-FC).^462^ This is an ideal system for neutron imaging because the presence of water provides excellent contrast. In neutron imaging, most of the contrast arises from absorption and by scattering neutrons out of the beam. For the latter, the total cross section (coherent + incoherent) is the relevant quantity, and the 82 barn cross section of ^1^H makes it optimal for imaging.

The change in scattering caused by the loss of materials was used to follow the pyrolysis of biomass,^463^ the same methodology would be applicable to follow the calcination and drying of a zeolite or the activation of a catalyst.

In addition to 2D radiography, neutron computed tomography can provide a 3D reconstruction of an object. This requires significantly longer time to acquire the data but does allow the object to be better viewed. An example using particulate filters for a diesel engine^464^ is shown in the upper right corner of Figure 62.

The nuclear-spin conversion of *ortho* (*o*-H_2_) to *para* (*p*-H_2_) hydrogen is important in fields as diverse as NMR spectroscopy, neutron scattering, and hydrogen storage. In the liquid phase at 20 K, the process takes days. However, it is catalyzed by paramagnetic materials, commonly γ-Fe_2_O_3_. At energies below the *J* = 0 → *J* = 1 rotational transition at 117 cm^–1^ of H_2_, the cross sections of *o*-H_2_ and *p*-H_2_ are very different. By selecting neutrons with an energy of 10–250 cm^–1^, *o*-H_2_ and *p*-H_2_ are readily distinguished. This was used to provide both spatial and kinetic information on the process *in situ*.^465^ (A video of the process is available in the Supporting Information of ref (465).) The cross sections of ice and liquid water are also different at low energies, and this is used to distinguish between them in a fuel cell.^466^

The conversion of CO_2_ with hydrogen into methane is an important step in the power-to-gas concept for the seasonal storage of renewable energy. The process is the Sabatier reaction (4H_2_ + CO_2_ → 2H_2_O + CH_4_) and uses a supported nickel catalyst. By removing the water that is formed, the yield can be enhanced. This can be done by sorption catalysts made of Ni particles embedded in a 5A zeolite matrix. The water generated during the reaction is readily observed by neutron imaging.^467,468^ The technique provides quantitative results on the amount and location of water in the reactor. The difference in the cross section between ^1^H and ^2^H means a large contrast between protonated and deuterated species, which enables steady-state isotopic transient kinetic analysis (SSITKA) to be carried out.

**Figure 63:**
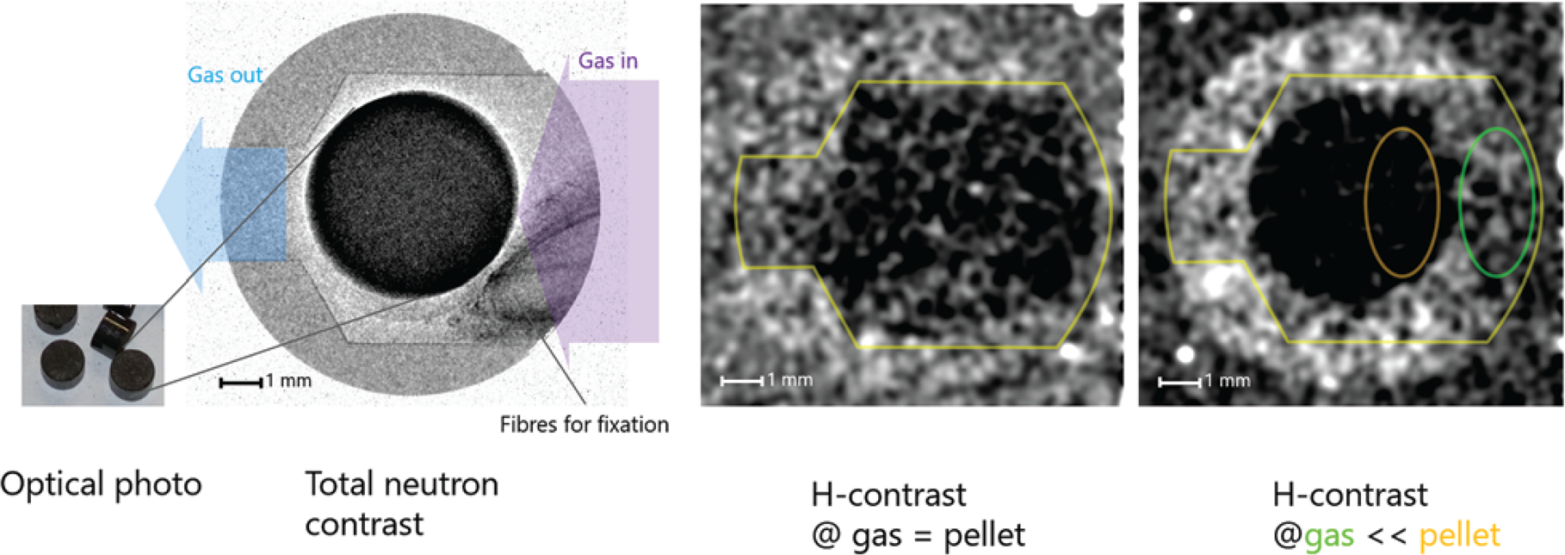
Neutron transmission image of a Cu/ZnO catalyst pellet (optical photo on the left) placed in an aluminum reactor (left large picture). The pellet’s cylindrical axis is aligned parallel to the neutron beam. Middle and right pictures show the neutron contrast image under different conditions, one with hardly any excess hydrogen adsorption in the catalyst, (middle) and one with marked adsorption in the catalyst exceeding that of the gas phase (right). The contrast is maximized for each image for better visibility. The yellow line is a guide to the eyes to indicate the reactor walls. Reproduced with permission from ref (469). Copyright 2020 Royal Society of Chemistry.

Although pivotal in heterogeneous hydrogenation reactions, the amount of hydrogen on catalysts during reactions is seldom known. Neutron imaging was used to follow and quantify hydrogen containing species in Cu/ZnO/Al_2_O_3_ catalysts during *operando* methanol synthesis.^469^Figure 63 shows the experimental arrangement and the catalyst pellet under low and high hydrogen conditions.

Steady-state measurements reveal that the amount of hydrogen-containing intermediates is related to the reaction yields of CO and methanol. Hydrogen–deuterium exchange experiments showed that hydrogen reduction modifies the catalyst so that, at operating temperatures, hydrogen is dynamically absorbed in the ZnO-nanoparticles. Thus, ZnO functions as a hydrogen reservoir, supplying hydrogen to the surface.

**Figure 64:**
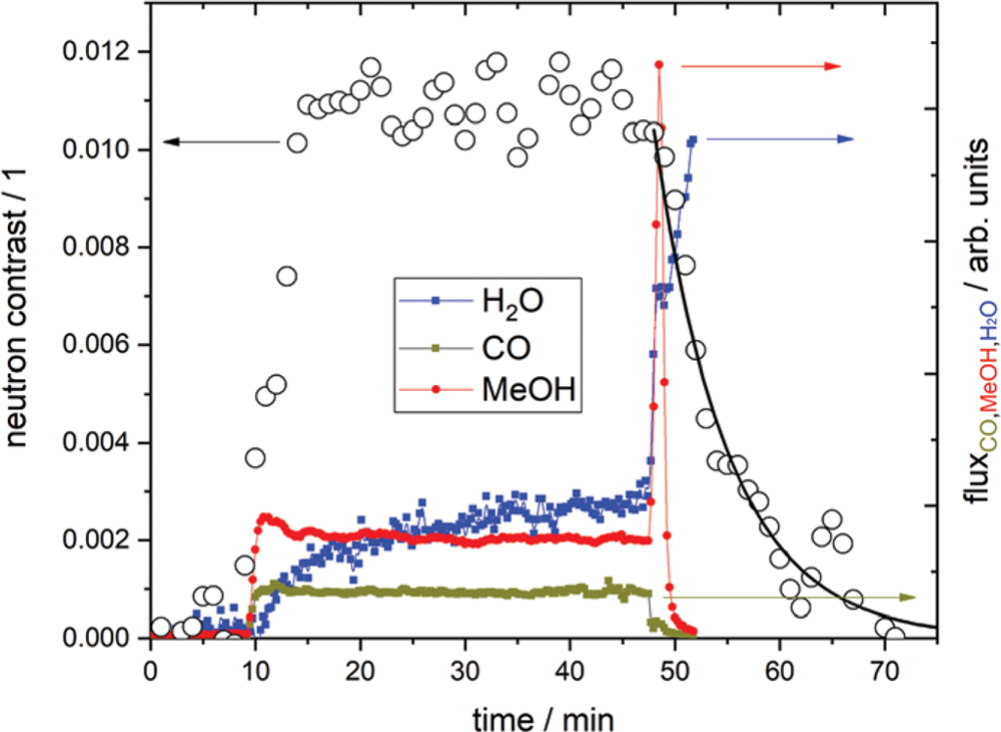
Time resolved *operando* measurements for a working methanol synthesis catalyst, Cu/ZnO/Al_2_O_3_, at 12 bar, 473 K, and H_2_:CO_2_ = 6. Reproduced with permission from ref (469). Copyright 2020 Royal Society of Chemistry.

Time resolved *operando* measurements are shown in Figure 64 for a working catalyst at 12 bar, 473 K, and with a H_2_:CO_2_ ratio of 6. The neutron contrast was used as a proxy for the amount of hydrogen on the catalyst. The surface reaches a steady-state rapidly after switching from H_2_ to H_2_/CO_2_ mixtures, as does the CO yield. The methanol yield has a brief maximum on the fresh sample, presumably because none of the sites are blocked. The water signal shows a slow increase with time. Switching off the CO_2_ and reducing the pressure to ambient conditions, cause the desorption of intermediates and products. The immediate decrease of the CO signal indicates that there is only little CO adsorbed. At the same time, the spike in methanol and water production demonstrate that a substantial number of methanol-related intermediates and the products themselves are adsorbed. This is also apparent by the slow decay of the neutron contrast (the line is a fit to an exponential function). The presence of surface intermediates agrees with the INS studies described earlier.^245^

In brief, neutron imaging of catalysts is in its infancy, but the potential is clear. The latest instruments that offer multiple contrast mechanisms, particularly wavelength resolved methods, combined with the use of H/D substitution, are ideally suited to studying catalytic processes. The technique is readily used *operando*. It will not achieve the atomic scale resolution possible with X-ray methods. Instead, it provides information about the location of hidden (e.g., by the can or support) materials within objects and how these change with time. Thermal and mass transport limitations mean that industrial chemical processes are carried out over relatively long (>seconds) times, and these are ideally suited to neutron imaging.

### 5.4 Multimodal Neutron Approaches: Integration of Two or More Neutron Techniques

Examples of using one or more neutron scattering techniques are less common than may initially be expected. In general chemistry, several examples of studies combining elastic and inelastic neutron scattering techniques are known. For instance, Rols and co-workers used a combination of the Institute Laue-Langevin’s D20 two-axis diffractometer and the IN6 time-of-flight spectrometer to obtain, respectively, the structure and generalized density of states of single-wall nanotubes.^470^ There are several examples of neutron techniques combined with photon-based spectroscopic probes such as infrared spectroscopy, for the characterization of heterogeneously catalyzed reaction systems. For example, Hawkins and co-workers used a combination of INS measurements complemented by infrared spectroscopy (in this case, DRIFTS) to evaluate the role of intermediates species in zeolite catalysis.^471^ A similar case of combining INS and DRIFTS was used to study the selective hydrogenation of acetylene over ceria surfaces.^247^ The combination of neutron scattering with online gas analysis was also demonstrated, such as *in situ* INS with a gas chromatograph,^472^ and *in situ* ND with mass spectrometry.^20^ However, there is a paucity of examples of multiple neutron techniques used in catalytic science. This is surprising, given the different perspectives and benefits that the wide range of neutron instrumentation can potentially provide.

#### 5.4.1 INS and QENS

One area of endeavor that is showing real promise, and is being increasingly taken up by several researchers, is to supplement INS investigations with QENS. INS provides access to the vibrational spectrum of a heterogeneous catalyst, which can be insightful in determining molecular/atomic entities retained within the catalyst matrix.^37^ Supplementing that perspective, QENS provides access to diffusional information on the atomic scale^300^ and is useful when examining samples that possess porous networks, such as zeolites or supported metal catalysts. In this way, QENS has found applications in assessing how mass transfer effects may be contributing to the performance of a particular catalytic system.^473^ Thus, a combined experimental approach has the potential to (i) discern what entities are engaged in the chemical transformation under consideration (INS) and (ii) determine whether the accumulation of such entities may be moderating mass diffusion within the solid matrix (QENS). Ultimately, these factors influence the critical parameters of catalytic activity and selectivity.

A single example is selected here to demonstrate the benefits of a combined INS and QENS investigation of a catalytic system: namely, the dynamics of 1-octene adsorption at 293 K on a ZSM-5 zeolite catalyst.^387^ The interaction of 1-octene with ZSM-5 is intended to be a model for gasoline-to-olefin chemistry that can play a role in fluidized catalytic cracking unit operations performed in petroleum refining facilities.^474^ The study is limited to a temperature of 293 K to assess the scope of the interaction between the linear alkene and the solid acid zeolite under conditions that avoid the formation of a wide number of product molecules, which would otherwise significantly complicate the analysis.

**Figure 65:**
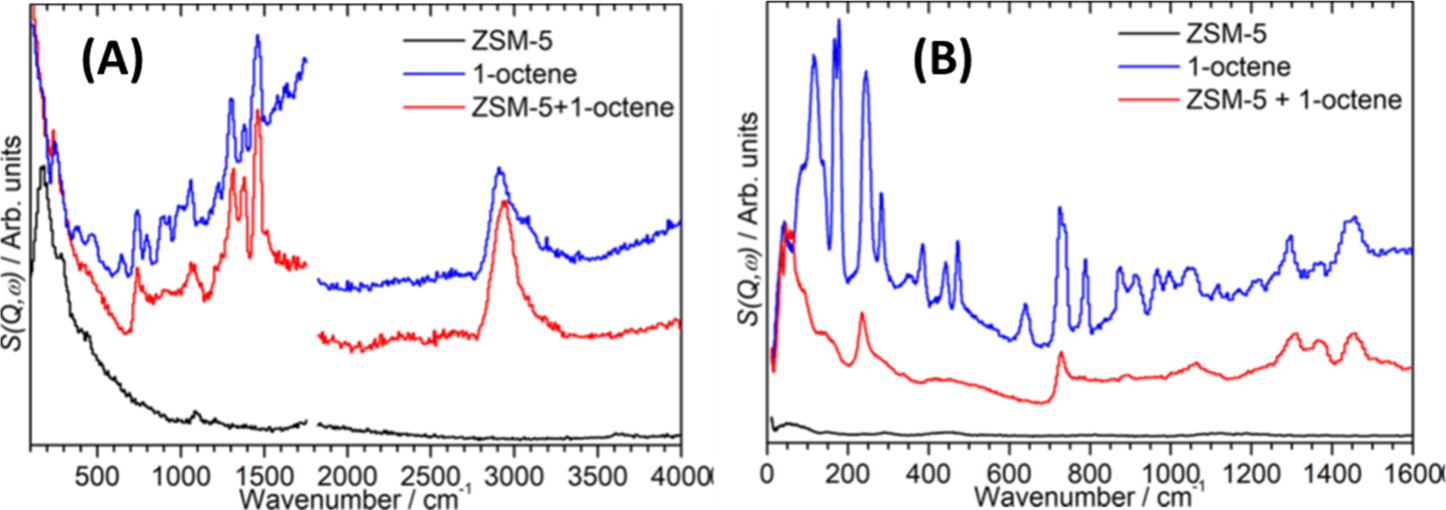
INS spectra (20–4000 cm^–1^) of clean ZSM-5 (black), pure 1-octene (blue), and 1-octene in ZSM-5 (red) recorded on (A) the MAPS spectrometer at incident energies of 2017 (left) and 5244 (right) cm^–1^, and on (B) the TOSCA spectrometer. Reproduced with permission from ref (387). Copyright 2019 American Chemical Society.

To obtain the vibrational spectrum at a fair resolution over the energy range 20–4000 cm^–1^, two INS spectrometers were utilized in the study:^31^ MAPS and TOSCA, both located at the ISIS Facility. Figure 65 presents a set of INS spectra recorded using the MAPS and TOSCA spectrometers. The increased resolution of TOSCA at low energies is readily apparent, as are the advantages of MAPS above ∼1200 cm^–1^.

On addition of the 1-octene to the zeolite, Figure 65A,B shows that the observed spectrum differs significantly from the spectra of the individual components, indicating chemisorption involving a degree of molecular rearrangement to be occurring. The intensity of the (C–H) stretch region above 3000 cm^–1^ is reduced relative to that observed for pure 1-octene, with the 2400–3600 cm^–1^ region of the ZSM-5/1-octene spectrum only showing contributions from the sp^3^ (C–H) stretch modes. Together with the complete disappearance of the (=CH_2_)-associated peaks at 639, 911, and 967 cm^–1^, the change in adsorption is attributed to the carbocation-forming protonation of the octene.

**Figure 66:**
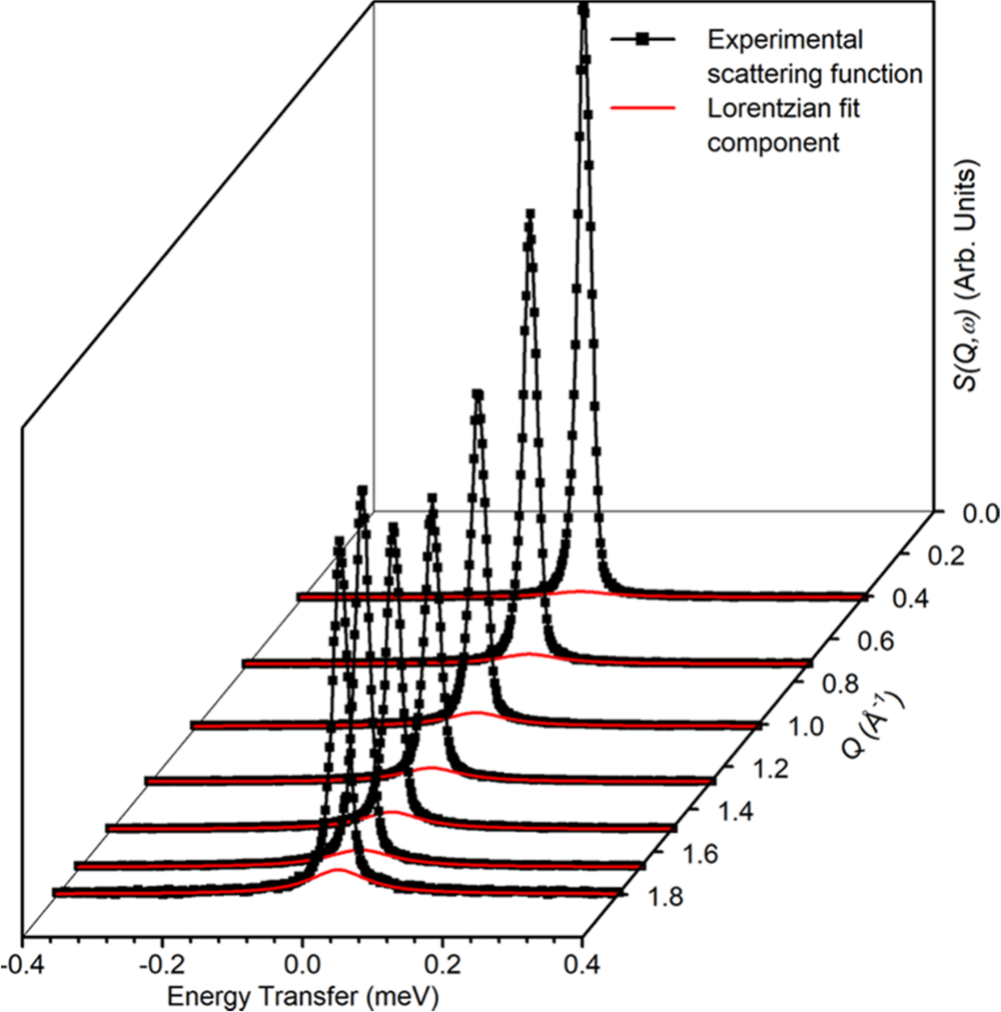
QENS spectra at selected *Q* values for 1-octene in ZSM-5 measured at 373 K following subtraction of the immobile zeolite contributions (black). The Lorentzian fit component modeling the quasielastic contribution at each *Q* value is also shown (red). Reproduced with permission from ref (387). Copyright 2019 American Chemical Society.

QENS spectra of ZSM-5 loaded with 1-octene at 293 K are presented in Figure 66 and exhibit a degree of quasielastic broadening that increases with the temperature of the QENS acquisition, indicating that motion occurs on the ∼2–50 ps time scales visible to the OSIRIS spectrometer^383^ utilized for these measurements. The identification of the exact motion responsible for the quasielastic intensity observed is simplified by the results of the vibrational analysis completed above. Since the hydrocarbon is a long chain constrained within zeolite pores whose diameter is less than the chain length, only a few types of rotation are physically possible: namely, the reorientation of the terminal methyl groups around their C–C bond, either continuously or as a series of 120° jumps between equivalent orientations, or the uniaxial rotation of the entire alkyl chain.^387^

Localized motions in a QENS spectrum may be characterized by deriving the Elastic Incoherent Structure Factor (EISF), a parameter that defines the fraction of the total scattering intensity that is elastic.^300,473^ Analysis of the experimental EISF values from Figure 66 as a function of momentum transfer (*Q*) indicates uniaxial rotation to be responsible for the majority of this quasielastic character and that contributions of other motions are likely to be negligible.^387^

From these combined results, the interaction of ZSM-5 and 1-octene at 293 K is characterized by the catalytic oligomerization of the 1-octene that leads to the formation of long-chain linear hydrocarbons that are large enough to be immobilized within the zeolite pores. This level of detailed and precise information on the selected adsorption system would only be achievable by applying the two neutron scattering techniques described.

#### 5.4.2 INS and ND

Combining both INS and ND is another area with promise for catalysis studies as they can tackle both the chemical nature (INS) and structure (ND) of catalysts and reaction species. As shown in the INS and ND sections, the structural evolution of the Ru/C12A7:e^–^ electride catalyst was followed by both *in situ* ND and INS during ammonia synthesis conditions.^20^ While the ND result (Figure 51) from the NOMAD beamline indicates the inclusion of extra species into the cage of the electride support, the INS spectra (Figure 28) obtained from the VISION beamline, aided with computational modeling, provide unambiguous evidence for the formation of hydride species in the C12A7 cages.

**Figure 67:**
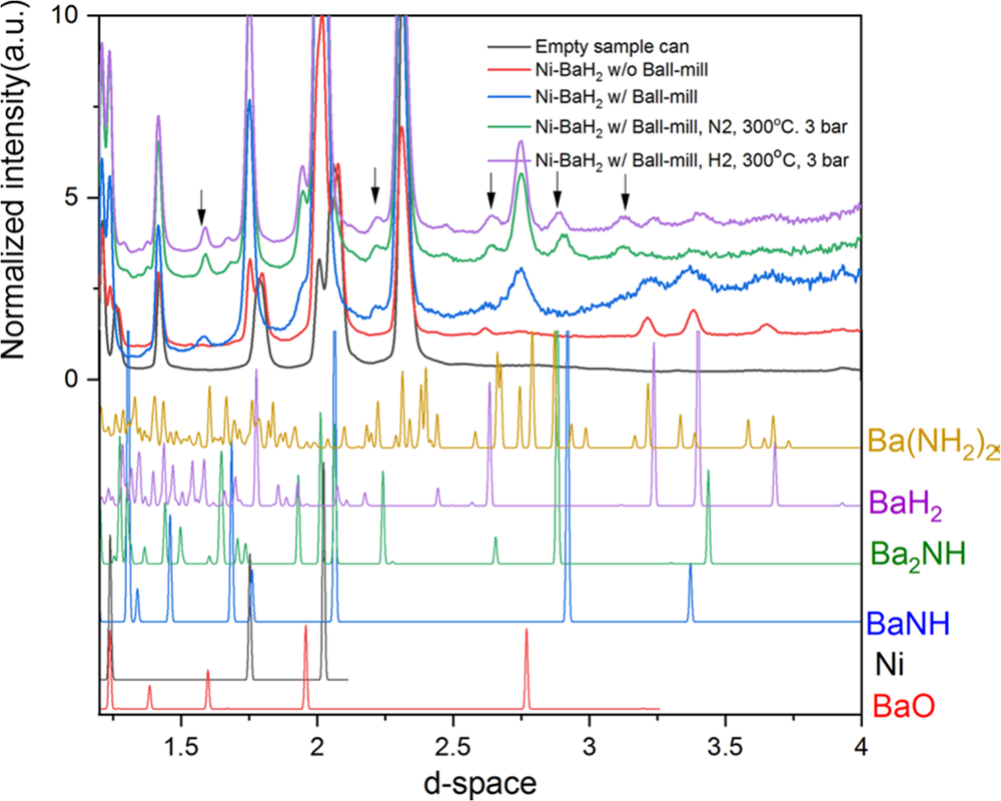
Neutron diffraction patterns collected at VISION for Ni/BaH_2_ with and without ball milling, and *in situ* during the N_2_–H_2_ cycling process. The peaks from several standards including Ba(NH_2_)_2_, BaH_2_, Ba_2_NH, BaNH, Ni, and BaO are also shown at the bottom. Reproduced with permission from ref (101). Copyright 2021 Springer. Corresponding *in situ* INS spectra are shown in Figure 29.

The power of combining INS and ND was recently demonstrated even at a single beamline—VISION at SNS for studying a Ni/BaH_2_ catalyst during N_2_–H_2_ chemical looping for ammonia synthesis.^101^ The INS spectra collected during the looping process are shown in Figure 29 in Section 3.3.1, and the corresponding ND patterns collected simultaneously are exhibited in Figure 67. The few extra diffraction peaks appearing at ∼1.68, 2.22, 2.64, 2.88, and 3.12 Å (indicated by arrows) after the N_2_ reaction step could be due to the formation of different types of NH species. By comparing the simulated patterns from BaH_2_, BaNH, Ba_2_NH, and Ba(NH_2_)_2_, it is clear that BaNH and BaH_2_ can account for most of the observed peaks, confirming the formation of BaNH in the nitridation step, as also observed in the INS spectra in Figure 29. The extra diffraction peaks remain after the H_2_ step, although peaks due to BaH_2_ also show up, suggesting a partial regeneration of the starting hydride material. This is again consistent with the INS results, where the hydrogenation of NH_*x*_ species is more difficult than the N_2_ fixation (nitridation) step. Interestingly, the diffraction intensity decreases after the N_2_ reaction step and regains after the H_2_ step, suggesting H abstraction and addition into the sample since the ND intensity is mainly from the incoherent scattering of H atoms.

In brief, the outcomes from the above few exemplar case studies of combining two neutron techniques are inspirational in encouraging more multitechnique neutron scattering investigations on other catalytic materials, adsorption, and/or reaction systems.

## 6 Conclusions and Perspectives

In this review, we have provided an overview of the progress made mainly in the past decade or so by neutron scattering studies of heterogeneous catalysis. It demonstrates that neutron scattering has provided significant insights into understanding the role of various hydrogen and hydrogenous species in catalytic reactions and played a unique role in revealing the structures of heterogeneous catalysts, including light atoms and neighboring elements. In each section, we have reviewed the theoretical fundamentals and the catalytic applications of major neutron scattering techniques, including inelastic neutron scattering (INS), neutron diffraction (ND), quasi-elastic neutron scattering (QENS), small angle neutron scattering (SANS), neutron imaging along with the combination of multiple neutron techniques. The examples provided show that INS can deliver distinctive perspectives on the chemical nature of adsorbates and sometimes surface reaction intermediates on catalysts, complementary to other techniques such as infrared and Raman spectroscopies. ND’s strengths are the location of light elements and distinguishing neighboring elements in the periodic table. It also gives better data at higher diffraction angles with stronger intensity than X-ray techniques. QENS excels in understanding the transport motions of hydrogen-containing species on catalyst surfaces and in catalyst pores at fast time scales (ps–ns). SANS is particularly powerful in studying ions and molecules’ adsorption, diffusion, location, and confinement in porous structures enabled by contrast variation. Neutron imaging is still in its infancy and has seen its major use in the study of water management in fuel cells and potentially electrochemistry at a spatial resolution down to the mm scale. The coupling of two or more neutron techniques, such as INS-ND and INS-QENS, is not common but has shown its power in catalysis research and should be encouraged.

It is noteworthy that computational modeling is indispensable to interpreting and understanding neutron scattering results. Lattice dynamics or molecular dynamics based on DFT have been used to help assignment of INS peaks and reveal the structural origin behind the experimental observations; the reverse Monte Carlo method has been employed to interpret the complicated neutron diffraction patterns measured on a disordered or nanomaterial and to search for structural models that can reproduce the total scattering intensities. Thanks to the recent advances in exascale computers and quantum chemistry, it is expected that the coupling of modeling and data science with experiments could further revolutionize the field of neutron scattering for catalyst research.

At the outset of this review, the aim was to provide a balanced overview of the state of neutron scattering of catalysis. However, it is apparent that neutron spectroscopy, i.e. INS, has dominated the catalysis research field over other neutron techniques and continues to do so. Why this is the case is not obvious. In part, this is because much of heterogeneous catalysis involves shuttling hydrogen atoms from one molecule to another and INS is especially sensitive to hydrogen. The transparency of most catalysts and supports to neutrons is an added advantage as it enables a wide spectral range to be accessed. However, we believe that the major reason is history: the first chemists to use neutron scattering came largely from a physical chemistry background, so it gravitated toward spectroscopic applications. Indirect geometry instruments provide data that is at least conceptually similar to that from infrared and Raman spectrometers, so these were the instruments of choice. The realization that INS could produce novel and useful information inspired others and spawned a virtuous circle of increasing usage, which resulted in the extensive sample environment options now available at INS beamlines.

With the ever-increasing drive for *in situ*/*operando* studies in catalysis, it is imperative that catalysis researchers and neutron scientists work closely together to develop and advance sample environments adapted for different neutron beamlines. Some promising developments have been recently made, for example, at the NOMAD beamline in SNS, as shown in Section 4.1. Neutron diffraction is one of the few neutron techniques where *in situ*/*operando* studies of catalysis have been demonstrated. However, demonstration examples with neutron imaging, QENS, and INS with direct geometry spectrometers do exist and provide a basis for further development.

As with any technique, there are threats as well as opportunities. The biggest problem faced by neutron scattering is the reduction in the number of neutron sources (mostly reactors) as a result of obsolescence or nuclear proliferation concerns. Closing sources increases the demand on the operating facilities and makes obtaining beam time more challenging. The continuing increase in brightness of synchrotron sources means that inelastic X-ray scattering is becoming competitive with INS for some applications. There is also an increasing expectation of “black box” operation, where no knowledge of the mechanics of the experiment are needed. This approach relies on the availability of experts to ensure that the results are reliable. These are becoming scarcer.

One area that has not been discussed is the use of polarized neutrons. As noted in Section 2, neutron scattering can be coherent or incoherent. For ^1^H, it is the incoherent scattering that is dominant; for most other elements (and isotopes), the coherent scattering is larger than the incoherent scattering. For hydrogenous materials, it is generally assumed that the coherent scattering can be either neglected (INS) or subtracted out (QENS). The use of polarized neutrons enables the coherent and incoherent scattering to be analyzed separately. Recent work suggests that for QENS, this assumption may not be completely valid.^475^ Generating polarized neutrons is a very inefficient process, so the technique is very flux-demanding but offers the possibility of better agreement between experiment and MD calculations for QENS.

Overall, it is clear from this review that neutron scattering in its various forms provides a useful supplement to more traditional catalyst characterization methods. Given the prominence of hydrogen as a fuel within the emerging Net Zero agenda (including the use of hydrogen chemical vectors such as ammonia and methanol) and the suitability of neutron techniques to monitor hydrogen, the recently realized expansion of neutron methods in catalysis research is expected to increase over the coming years. That said, the limited number of neutron facilities around the globe needs to be recognized as a potentially rate-limiting parameter. Investment in existing and new facilities and *in situ*/*operando* sample environments is necessary to continue facilitating advances in catalytic science and energy research required by our modern society.

## Supporting Information Available

Neutron sources listed in Table S1; INS studies of catalysts, adsorbates, and reaction species are briefly summarized in Table S2 (PDF)

## ABBREVIATIONS

CSNS: China Spallation Neutron Source
DFT: Density Functional Theory
DGS: Direct Geometry Spectrometer
DM: Dispersion Medium
DME: Dimethyl Ether
EISF: Elastic Incoherent Structure Factor
ESS: European Spallation Source
FTS: Fischer–Tropsch Synthesis
HEO: High-Entropy Oxide
IGS: Indirect Geometry Spectrometer
ILL: Institut Laue Langevin (France)
INS: Inelastic Neutron Scattering
IPA: Isopropyl Alcohol
ISIS: ISIS Neutron and Muon Source (UK)
MD: Molecular Dynamics
MTH: Methanol-To-Hydrocarbon
ND: Neutron Diffraction
NMR: Nuclear Magnetic Resonance
NPs: Nanoparticles
NPDF: Neutron powder diffractometer
NVS: Neutron Vibrational Spectroscopy
ORNL: Oak Ridge National Laboratory
PDF: Pair Distribution Function
PEM-FC: Proton-Exchange Membrane Fuel Cell
QENS: Quasi-elastic Neutron Scattering
SANS: Small-Angle Neutron Scattering
SAXS: Small-Angle X-ray Scattering
SEM: Scanning Electron Microscopy
SINQ: Swiss Spallation Neutron Source (Switzerland)
SNS: Spallation Neutron Source (USA)
SSITKA: Steady-State Isotopic Transient Kinetic Analysis
STS: Second Target Station at SNS (USA)
TAS: Triple Axis Spectrometer
THF: Tetrahydrofuran
ToF: Time-of-Flight
TOS: Time on Stream
WGS: Water–Gas Shift
