# Supplementary material for: Neutron Scattering Studies of Heterogeneous Catalysis
Source: Chem Rev. 2023 Jun 14;123(13):8638–700. doi: 10.1021/acs.chemrev.3c00101 (PMC10347434; doi:10.1021/acs.chemrev.3c00101)
Supplement: Supplementary file 1 — cr3c00101_si_001.pdf [file cr3c00101_si_001.pdf]

Supporting Information for:

Neutron Scattering Studies of Heterogeneous Catalysis

Xinbin Yu[1], Yongqiang Cheng[2], Yuanyuan Li[1], Felipe Polo-Garzon[1], Jue Liu[2], Eugene Mamontov[2], Meijun Li[3], David Lennon[4]*, Stewart F. Parker[5]*, Anibal J. Ramirez-Cuesta[6]*, and Zili Wu[1,7]*

1. Chemical Sciences Division, Oak Ridge National Laboratory, Oak Ridge, Tennessee 37381, United States.
2. Neutron Scattering Division, Oak Ridge National Laboratory, Oak Ridge, Tennessee 37831, United States.
3. Manufacturing Science Division, Oak Ridge National Laboratory, Oak Ridge, Tennessee 37831, United States.
4. School of Chemistry, Joseph Black Building, University of Glasgow, Glasgow, G12 8QQ, United Kingdom.
5. ISIS Pulsed Neutron and Muon Facility, STFC Rutherford Appleton Laboratory, Chilton, Didcot, Oxon, OX11 0QX, United Kingdom.
6. Neutron Technologies Division, Oak Ridge National Laboratory, Oak Ridge, Tennessee 37831, United States
7. Center for Nanophase Materials Sciences, Oak Ridge National Laboratory, Oak Ridge, Tennessee 37831, United States

* Corresponding authors: DL, email: David.Lennon@glasgow.ac.uk; SFP, email: stewart.parker@stfc.ac.uk; AJRC, email: ramirezcueaj@ornl.gov; ZW, email: wuz1@ornl.gov

Table S1. List of current neutron sources with location and type.

Name	Location	Type
Institute for Energy Technology (IFE)	Kjeller, Norway	Reactor
European Spallation Source (ESS)	Lund, Sweden	Spallation source
ISIS Pulsed Neutron Source	Oxford, UK	Spallation source
Reactor Institute Delft	TU Delft, Delft, Netherlands	Reactor
Helmholtz-Zentrum Berlin (HZB)	Berlin, Germany	
Heinz Maier-Leibnitz Zentrum (MLZ)	Garching, Germany	
TRIGA User Facility	Johannes Gutenberg-Universitat Mainz, Mainz, Germany	
National Centre for Nuclear Research – MARIA	Otwock-Swierk, Poland	
Laboratoire Léon Brillouin (LLB)	Gif-sur-Yvette, France	
Institut Laue-Langevin (ILL)	Grenoble, France	
Nuclear Physics Institute (NPI)	Prague, Czech Republic	
ESS Bilbao	Bilbao, Spain	
SINQ - Paul Scherrer Institut (PSI)	Villigen, Switzerland	Spallation source
Atominstitut - TU Wien	Vienna, Austria	Reactor
Budapest Neutron Centre BNC – AEKI	Budapest, Hungary	
TRIGA, Reactor Infrastructure Centre (RIC)	Slovenia, Ljubljana, Slovenia	
Portuguese Research Reactor (RPI)	Sacavem, Portugal	
Demokritos	Athens, Greece	
Petersburg Nuclear Physics Institute	Gatchina, Russia	
Joint Institute for Nuclear Research (FLNP/JINR)	Dubna, Russia	
Nuclear Energy Corporation of South Africa	Pelindaba, South Africa	
Instituto Peruano de Energía Nuclear	Lima, Peru	
Laboratório de Metrologia de Nêutrons (LN)	Rio de Janeiro, Brazil	

Laboratorio Argentino de Haces de Neutrones (LAHN)	Buenos Aires, Argentina	
Centro Atómico Bariloche	Bariloche, Argentina	
TRIUMF	Vancouver, Canada	
McMaster Nuclear Reactor	Hamilton, Canada	
Canadian Neutron Beam Centre	Chalk River, Canada	
Los Alamos Neutron Science Centre (LANSCE)	Los Alamos, USA	
University of Missouri Research Reactor (MURR)	Columbia, USA	
The Low Energy Neutron Source of Indiana University (LENS)	Bloomington, Indiana, USA	
Oak Ridge National Laboratory Neutron Sciences	Oak Ridge, Tennessee, USA	Spallation source
NIST Centre for Neutron Research	Gaithersburg, Maryland, USA	
MIT Nuclear Reactor	Cambridge, Massachusetts, USA	Reactor
Kalpakkam Mini reactor (KAMINI)	Kalpakkam, Mumbai, India	
China Mianyang Research Reactor (CMRR)	Mianyang, China	Spallation source
CARR	Beijing, China	
High-flux Advanced Neutron Application Reactor (HANARO)	Taejon, Korea	Reactor
Kyoto University Research Reactor Institute (KURRI)	Kyoto, Japan	Spallation source
Institute of Materials Structure Science (IMSS)	Tsukuba, Japan	
CROSS-Tokai	Tokai, Japan	
Reactor Triga Puspati (RTP)	Malaysia	
National Nuclear Energy Agency of Indonesia (BATAN)	Indonesia	Reactor
Australian Centre for Neutron Scattering & National Deuteration Facility - ACNS & NDF	Kirrawee, Australia	

Table S2. Observed INS frequencies for H-containing species over various catalysts

	Catalyst	Species	INS bands (cm^{-1})	Assignment	ref
**Hydride**	CeO_2	Ce-H	400-650	Ce−H deformation of surface hydride, possible additional contribution from bulk CeH_3-like local structure	1
			750-1100	Ce−H deformation in bulk hydride with CeH_2-like and CeH_3-like local structures	
			1300−1800	Combination and overtone of deformation modes of peaks at 400-650 and 750-1100 cm^{-1}	
	ZnO	Zn-H	829	Bending mode of Zn-H	2
			1665	Symmetric stretching mode of Zn-H	
	Lindlar catalyst (5% Pd/$CaCO_3$)	Pd-H	484, 968	β-PdH	3
			552	α-PdH	
	Pd		500	Pd-H	4
	Pd black		740/820, 970	H in C_{3v} sites at the surface(Pd–H stretch)	5
	Pd(5%)/C, Pd(4.5%)Pt(0.5%)/C, Pd(4.5%)Pt(0.5%)Fe(5%)/C		480	β-PdH	6
	20% Pd on activated carbon (Pd/AC)		480	β-PdH	7
			~560	α-PdH	
	20% Pd on carbon black (Pd/CB)		470	Sub-surface hydrogen	
			485, 1100, 1640	β-PdH	
			760/820	Asymmetric Pd–H stretch modes of hydrogen in a threefold surface site on (111) facets	
			980	Symmetric Pd–H stretch modes of hydrogen in a threefold surface site on (111) facets	
			2150	H at the on-top site of Pd	
	Pt black	Pt-H	532, 621	H bound on two- or threefold sites	8

	Pt/C		520, 950 and part of the intensity at 1300	H on Pt(1 1 1) faces	9
			640	Doubly degenerate asymmetric stretch of H on Pt(1 0 0) faces	
			550	Doubly degenerate asymmetric stretch of H on Pt(1 0 0) faces	
			470	Bending mode of H on the on-top site	
			2079	Pt–H stretch mode	
	Pt/Al_2O_3		470, 535, 590, 670, 750, 800−1200	Pt−H vibrations of n-fold coordinated (bridged, hollow, and 4-fold coordinated) Pt−H species	10
	Pt(58 %)/C fuel cell catalyst		480	On-top Pt-H bending mode	11
	Pt/Al_2O_3		240-850	Bending mode of all Pt-H	12
			775-1650	Stretching modes of multi-folded Pt-H	
			1650-1810, 1930-2000	Stretching modes of interfacial Pt-H	
			2065-2275	Stretching modes of top Pt-H	
	Ni-alumina/silica	Ni-H	~1000	Ni-H species	13
	$CeNi_xH_yO_z$		870	Ni-H	14
	Ni foam		900 and 1030	Ni(111)-H	15
			400-800	Ni-non (111)-H	
	Raney-Ni		600	The symmetric stretching of μ_4–H species adsorbed on (100) facets	16
			800	The antisymmetric stretching of μ_3–H species adsorbed on (110) facets	
			940	The antisymmetric stretching of μ_3–H species adsorbed on (111) facets	
			1100	The symmetric stretching of μ_3–H species adsorbed on (110) facets	

			1130	The symmetric stretching of μ_3–H species adsorbed on (111) facets	
			1800	The stretching vibrations of μ_1–H species (on-top hydrogen); the bending mode is expected between 800 and 1130 cm^{-1}, and is thus hidden by the more intense features due to multiply bound hydrogen	
	Skeletal Co	Co-H	988 and 1102	H bound in the threefold-site (*i.e.* Co_3H)	17
	Raney Co		620, 900, 1145	H on Co	18
	Co foam		725		
	CoCuMn/C		800, 1650		
	Raney-Co		~250	Hydrogen on 4-fold sites with D4h symmetry (i.e., 101 plane); Co_4–H symmetric stretching vibrations	16
			~573	Hydrogen on 3-fold sites with C3v symmetry (i.e., 001 plane); Co_3–H antisymmetric stretching vibrations.	
			~637	Hydrogen on 3-fold sites with C3v symmetry (i.e., 101 plane); Co_3–H antisymmetric stretching vibrations. Probably some contribution from 4-fold sites (‘‘hollow sites’’) with D4h symmetry: Co_2–H antisymmetric stretching vibrations.	
			~782	Hydrogen on 3-fold sites with C3v symmetry (i.e., 101 plane); Co_2–H asymmetric stretching vibrations.	
			~894	Hydrogen on 3-fold sites with C3v symmetry (i.e., 001 plane); Co_2–H antisymmetric stretching vibrations.	
			~1100	Hydrogen on 3-fold sites with C3v symmetry (i.e., 001 and 101 planes); Co_3–H symmetric stretching vibrations.	
	CuH	Cu-H	1070, 2080	CuH	19
	CuH/pyridine-D5		1060	CuH	20
	$CuCrFeO_x$		770-780	Cu-H	21

	γ-Mo_2N	Mo-H	658, 986, and 1324	μ^6-Mo_6H_{sub}	22
	BaH_2	Ba-H	400–700	Vibrations of the 5-coordinated H in an approximate square-pyramidal structure	23
			700–1100	Vibrations of the 4-coordinated H in a tetragonal structure	
**H_2**	Co/C	H_2/HD	46	H_2 coordinated to Co	18
			116	H_2 physisorbed on graphite	
	Co foam		49	H_2 coordinated to Co	
	CoNa-A zeolite		123	Perpendicular vibration of H_2 adsorbed in CoNa-A	24
	Graphite		156	Out-of-plane vibration of H_2 adsorbed on graphite	
			120	Out-of-plane vibration of HD adsorbed on graphite	
	Ca exchanged Na-Y		32.3 and 45.3	Rotational transitions of H_2 coordinated to cations in zeolites	25
	Co exchanged AlPO		58.4		26
	Fe-ZSM5		±4.2, 8.3	H_2 molecules at Fe-ZSM5	27
**Hydroxide**	$CeNi_{0.2}$	Ce-OH	660	Ce(Ni)-OH deformation band	28
	cubic $H_{0.4}WO_3$	M-OH	1145-1170	M-O-H deformation	29
	H_xVO_2	V-OH	909 and 1083	Orthogonal δ-V-OH bending modes	30
	$H_xV_9Mo_6O_{40}$ (x=7.8 and 17.5)	V-OH, Mo-OH	~1081	Combination of Mo-OH and V-OH deformation mode	31
	$H_{0.35}MoO_3$	Mo-OH	968	-OH deformation vibration band	32
	$H_{0.34}MoO_3$		1267	-OH deformation vibration band	29
	monoclinic phases H_xMoO_3 (*e.g.* x=0.93, 1.68, 2.0)	$-OH_2$	1600	H-O-H deformation vibration	29
	Cu/MgO (CM)	MgO-OH	3600	O–H stretch of surface hydroxyl on the metal oxide	33
		Mg-OH	685	M–O–H bend of surface hydroxyl on the metal oxide	
			765	Mg–O–H bend of surface hydroxyl	
	Al_2O_3-promoted Cu/ZnO (CZ)	M–O–H	760	M–O–H bend of surface hydroxyl on the metal oxide	

		Cu-OH	3430	O–H stretch of surface hydroxyl on Cu	
			920	Cu–O–H bend of surface hydroxyl on Cu	
	Raney Co	-OH	3630, ~ 3400	O–H stretch	18
			850, 1700	Co–O–H bend	
			890, 3720	OH on Al_2O_3	
			420, 3550	Co-OH	
	CoCuMn/C		3590	O–H stretch mode	
	Co/C		3490	Co-OH	
	5%Pd/C		887	Pd-OH	3
	$Pd/CaCO_3$		1637	H-O-H scissor mode	
	Al_2O_3		550, 640, 905	Deformation modes of hydroxyl groups at the alumina surface	10
	Pt/Al_2O_3		200 - 1200	Bending and deformation modes of the −OH groups at the Al_2O_3 surface	12
**Peroxide**	Au/TiO_2	-OOH	1230	δ_{as}-(OOH) mode of the hydrogen peroxide species	34
**Sulfhydryl**	MoS_2	-S-H	1400	-S-H	35
	MoS_2		650	-SH bending band	36
	WS_2		694		
	RuS_2		600, 710		
**Amino**	γ-Mo_2N	-NH	800 and 832	κ^1-NH_{surf}	22
	BaNH	NH^{2-}	583	Librational mode of NH^{2-} ions in the tetragonal low-temperature phase (probably coupled to a translation)	37
	$K_{0.97}(NH_4)_{0.03}Br$	NH_4^+	242	Librational mode of NH_4^+	38
**Hydrocarbons**	bulk copper formate	C-H	~1080	Out-of-plane C-H deformation mode	39
			~1389	In-plane C-H deformation mode	
	Al_2O_3-promoted Cu/ZnO (CZ)	Methoxy	95	Methyl torsion of adsorbed methoxy	33
			1160	Methyl rock of adsorbed methoxy	
			1450	OC–H bending modes of adsorbed methoxy	
			1460	OC–H bending modes of adsorbed methoxy	
			2940	C–H stretch of adsorbed methoxy	

		Methoxy and formate	2970	C–H stretch of adsorbed methoxy and formate	
		Formate	1055	Out-of-plane O_2C–H bend of adsorbed formate	
			1375	In-plane O_2C–H bend of adsorbed formate	
	Cu/MgO (CM)	Methoxy	95	Methyl torsion of adsorbed methoxy	
			1165	Methyl rock of adsorbed methoxy	
			1450	OC–H bending modes of adsorbed methoxy	
			1460	OC–H bending modes of adsorbed methoxy	
			2940	C–H stretch of adsorbed methoxy	
		Methoxy and formate	2970	C–H stretch of adsorbed methoxy and formate	
		Formate	1375	In-plane O_2C–H bend of adsorbed formate	
	CeO_2	Methyl	300	Methyl torsion in ethane	40
	Ni/Al_2O_3	C-H	840	Aromatic out-of-plane C-H bending mode	41
			1200	Aromatic in-plane C-H bending mode	
			3050	sp^2 carbon C-H stretch mode	
		C-C and C-H	1410	Coupled C-C stretch and C-H bend	
		C-C	620	sp^2 carbon network deformation mode	
	Na-exchanged zeolite X	Methanol	1580, 1450	$-CH_3$ and -OH in-plane deformation	42
			1160, 1115	$-CH_3$ rocking modes, possibly combined with -OH bending modes	
			1018	Stretching vibration of C-O	
			650, 760	Deformation vibration modes of H-bonded -OH	
			263, 122, 95	Twisting mode of $-CH_3$ and translation of methanol and methanol-cation cluster	
	η-Al_2O_3		84	Methyl torsion	43
			1170	CH_3 rock	
			1460	CH_3 deformation modes	

			2600	Combination of the CH_3 rock and the CH_3 deformation modes of the chemisorbed methoxy species	
	Cu_2O	Formate	208–225	Torsional mode of formate bridging two Cu atoms	44
			1090	Out-of-plane C–H bends	
			1399	In-plane C–H bends	
	CuO		208–225	Torsional mode of formate bridging two Cu atoms	
			1079	Out-of-plane C–H bends	
			1381	In-plane C–H bends	
			3056	C–H stretch	
	H_2-reduced CuO		208–225	Torsional mode of formate bridging two Cu atoms	
			1084	Out-of-plane C–H bends	
			1383	In-plane C–H bends	
	MFM-102-NO_2	Acetylene	< 201	Translational modes of C_2H_2 molecules	45
			645	Asymmetric C−H bending mode of C_2H_2	
			766	Symmetric C−H bending mode of C_2H_2	
	Skeletal Co	Ethylene	300	CH_3 torsion from a metal-bound methyl species	
			2940	C–H stretch from a saturated hydrocarbon	
	Raney-Co	Trans-$CD_3CH_2NH_2$	245	NH_2 rock	46
			375	CCN bend	
			664	CH_2 rock	
			745	CD_3/bend	
			891	NH_2 wag	
			1036	CC stretch	
			1132	CN stretch	
			1293	CH_2 twist	
			1359	CH_2 wag	
			1455	CH_2 scission	
			1575	NH_2 scission	
	5%Pd/C	C	645-1008	Out-of-plane aryl C-H deformation bands of the activated carbon supports	3

			1049-1291	In-plane aryl C-H deformation bands of the activated carbon supports	
	Pd on activated carbon (Pd/AC)		600	Graphene deformations	7
			750–900	Out-of-plane C–H bend	
			1050–1300	In-plane C–H bend	
	Pd	CH_3	302	Torsion of CH_3 having C_{3v} symmetry, bound to the on-top site on Pd(111) facets	5

References

(1) Wu, Z. L.; Cheng, Y. Q.; Tao, F.; Daemen, L.; Foo, G. S.; Nguyen, L.; Zhang, X. Y.; Beste, A.; Ramirez-Cuesta, A. J. Direct Neutron Spectroscopy Observation of Cerium Hydride Species on a Cerium Oxide Catalyst. *J. Am. Chem. Soc.* **2017**, *139*, 9721-9727.

(2) Kiss, J.; Witt, A.; Meyer, B.; Marx, D. Methanol Synthesis on ZnO (000 1). I. Hydrogen Coverage, Charge State of Oxygen Vacancies, and Chemical Reactivity. *J. Chem. Phys.* **2009**, *130*, 184706.

(3) Albers, P. W.; Möbus, K.; Frost, C. D.; Parker, S. F. Characterization of β-palladium Hydride Formation in the Lindlar Catalyst and in Carbon-supported Palladium. *J. Phys. Chem. C* **2011**, *115*, 24485-24493.

(4) Akiba, H.; Kofu, M.; Kobayashi, H.; Kitagawa, H.; Ikeda, K.; Otomo, T.; Yamamuro, O. Nanometer-size Effect on Hydrogen Sites in Palladium Lattice. *J. Am. Chem. Soc.* **2016**, *138*, 10238-10243.

(5) Albers, P. W.; Parker, S. F. Applications of Neutron Scattering in Technical Catalysis: Characterisation of Hydrogenous Species on/in Unsupported and Supported Palladium. *Top. Catal.* **2021**, *64*, 603-613.

(6) Möbus, K.; Grünewald, E.; Wieland, S.; Parker, S.; Albers, P. Palladium-catalyzed Selective Hydrogenation of Nitroarenes: Influence of Platinum and Iron on Activity, Particle Morphology and Formation of β-Palladium Hydride. *J. Catal.* **2014**, *311*, 153-160.

(7) Parker, S. F.; Walker, H. C.; Callear, S. K.; Grünewald, E.; Petzold, T.; Wolf, D.; Möbus, K.; Adam, J.; Wieland, S. D.; Jiménez-Ruiz, M. The Effect of Particle Size, Morphology and Support on the Formation of Palladium Hydride in Commercial Catalysts. *Chem. Sci.* **2019**, *10*, 480-489.

(8) Rush, J. J.; Cavanagh, R. R.; Kelley, R. D.; Rowe, J. M. Interaction of Vibrating H Atoms on the Surface of Platinum Particles by Isotope Dilution Neutron Spectroscopy. *J. Chem. Phys.* **1985**, *83*, 5339-5341.

(9) Parker, S. F.; Frost, C. D.; Telling, M.; Albers, P.; Lopez, M.; Seitz, K. Characterisation of the Adsorption Sites of Hydrogen on Pt/C Fuel Cell Catalysts. *Catal. Today* **2006**, *114*, 418-421.

(10) Carosso, M.; Vottero, E.; Lazzarini, A.; Morandi, S.; Manzoli, M.; Lomachenko, K. A.; Ruiz, M. J.; Pellegrini, R.; Lamberti, C.; Piovano, A. Dynamics of Reactive Species and Reactant-induced Reconstruction of Pt Clusters in Pt/Al_2O_3 Catalysts. *ACS Catal.* **2019**, *9*, 7124-7136.

(11) Parker, S. F.; Mukhopadhyay, S.; Jiménez-Ruiz, M.; Albers, P. W. Adsorbed States of Hydrogen on Platinum: A New Perspective. *Chem. - Eur. J.* **2019**, *25*, 6496-6499.

(12) Vottero, E.; Carosso, M.; Ricchebuono, A.; Jiménez-Ruiz, M.; Pellegrini, R.; Chizallet, C.; Raybaud, P.; Groppo, E.; Piovano, A. Evidence for H_2-Induced Ductility in a Pt/Al_2O_3 Catalyst. *ACS Catal.* **2022**, *12*, 5979-5989.

(13) Terreni, J.; Sambalova, O.; Borgschulte, A.; Rudić, S.; Parker, S. F.; Ramirez-Cuesta, A. J. Volatile Hydrogen Intermediates of CO_2 Methanation by Inelastic Neutron Scattering. *Catalysts* **2020**, *10*, 433.
(14) Fang, W.; Pirez, C.; Paul, S.; Capron, M.; Jobic, H.; Dumeignil, F.; Jalowiecki-Duhamel, L. Room Temperature Hydrogen Production from Ethanol over $CeNi_XH_ZO_Y$ Nano-Oxyhydride Catalysts. *ChemCatChem* **2013**, *5*, 2207-2216.
(15) Jobic, H.; Renouprez, A. Inelastic Neutron Scattering Spectroscopy of Hydrogen Adsorbed on Raney Nickel. *J. Chem. Soc., Faraday Trans. 1* **1984**, *80*, 1991-1997.
(16) Chojecki, A.; Jobic, H.; Jentys, A.; Müller, T. E.; Lercher, J. A. Inelastic Neutron Scattering of Hydrogen and Butyronitrile Adsorbed on Raney-Co Catalysts. *Catal. Lett.* **2004**, *97*, 155-162.
(17) Jones, E.; Inns, D. R.; Dann, S. E.; Silverwood, I. P.; Kondrat, S. A. Characterisation of Ethylene Adsorption on Model Skeletal Cobalt Catalysts by Inelastic and Quasi-elastic Neutron Scattering. *Catalysis Communications* **2022**, *163*, 106409.
(18) Davidson, A. L.; Lennon, D.; Webb, P. B.; Albers, P. W.; Berweiler, M.; Poss, R.; Roos, M.; Reinsdorf, A.; Wolf, D.; Parker, S. F. The Characterisation of Hydrogen on Nickel and Cobalt Catalysts. *Top. Catal.* **2021**, *64*, 644-659.
(19) Bennett, E. L.; Wilson, T.; Murphy, P. J.; Refson, K.; Hannon, A. C.; Imberti, S.; Callear, S. K.; Chass, G. A.; Parker, S. F. Structure and Spectroscopy of CuH Prepared *via* Borohydride Reduction. *Acta. Crystallogr. B. Struct. Sci. Cryst. Eng. Mater.* **2015**, *71*, 608-612.
(20) Bennett, E.; Wilson, T.; Murphy, P. J.; Refson, K.; Hannon, A. C.; Imberti, S.; Callear, S. K.; Chass, G. A.; Parker, S. F. How the Surface Structure Determines the Properties of CuH. *Inorg. Chem.* **2015**, *54*, 2213-2220.
(21) Polo-Garzon, F.; Fung, V.; Nguyen, L.; Tang, Y.; Tao, F.; Cheng, Y. Q.; Daemen, L. L.; Ramirez-Cuesta, A. J.; Foo, G. S.; Zhu, M. H. Elucidation of the Reaction Mechanism for High-Temperature Water Gas Shift over an Industrial-Type Copper–Chromium–Iron Oxide Catalyst. *J. Am. Chem. Soc.* **2019**, *141*, 7990-7999.
(22) Wyvratt, B. M.; Gaudet, J. R.; Pardue, D. B.; Marton, A.; Rudic, S.; Mader, E. A.; Cundari, T. R.; Mayer, J. M.; Thompson, L. T. Reactivity of Hydrogen on and in Nanostructured Molybdenum Nitride: Crotonaldehyde Hydrogenation. *ACS Catal.* **2016**, *6*, 5797-5806.
(23) Moon, J.; Cheng, Y. q.; Daemen, L.; Novak, E.; Ramirez-Cuesta, A. J.; Wu, Z. L. On the Structural Transformation of Ni/BaH_2 during a N_2-H_2 Chemical Looping Process for Ammonia Synthesis: A Joint In Situ Inelastic Neutron Scattering and First-Principles Simulation Study. *Top. Catal.* **2021**, *64*, 685-692.
(24) Nicol, J. M.; Eckert, J.; Howard, J. Dynamics of Molecular Hydrogen Adsorbed in CoNa-A Zeolite. *J. Phys. Chem.* **1988**, *92*, 7117-7121.
(25) Eckert, J.; Nicol, J. M.; Howard, J.; Trouw, F. R. Adsorption of Hydrogen in Ca-exchanged Na-A Zeolites Probed by Inelastic Neutron Scattering Spectroscopy. *J. Phys. Chem.* **1996**, *100*, 10646-10651.
(26) Ramirez-Cuesta, A. J.; Mitchell, P. C. H.; Parker, S. F. An Inelastic Neutron Scattering Study of the Interaction of Dihydrogen with the Cobalt Site of a Cobalt Aluminophosphate Catalyst: Two-Dimensional Quantum Rotation of Adsorbed Dihydrogen. *J. Mol. Catal. A: Chem.* **2001**, *167*, 217-224.
(27) Mojet, B. L.; Eckert, J.; van Santen, R. A.; Albinati, A.; Lechner, R. E. Evidence for Chemisorbed Molecular Hydrogen in Fe-ZSM5 from Inelastic Neutron Scattering. *J. Am. Chem. Soc.* **2001**, *123*, 8147-8148.
(28) Lamonier, C.; Payen, E.; Mitchell, P. C. H.; Parker, S. F.; Mayers, J.; Tomkinson, J. Hydrogen Species in Cerium—Nickel Oxides Catalysts: Inelastic and Compton Neutron Scattering Studies. In *Studies in Surface Science and Catalysis*, Vol. 130; Elsevier, 2000; pp 3161-3166.
(29) Dickens, P. G.; Birtill, J. J.; Wright, C. J. Elastic and Inelastic Neutron Studies of Hydrogen Molybdenum Bronzes. *J. Solid State Chem.* **1979**, *28*, 185-193.

(30) Chippindale, A. M.; Dickens, P. G.; Powell, A. V. Synthesis, Characterization, and Inelastic Neutron Scattering Sudy of Hydrogen Insertion Compounds of VO_2 (rutile). *J. Solid State Chem.* **1991**, *93*, 526-533.
(31) Slade, R. C. T.; Ramanan, A.; Nicol, J. M.; Ritter, C. Synthesis, Characterization and Inelastic Neutron Scattering Spectra of Hydrogen Insertion Compounds of the Mixed VMo Oxide $V_9Mo_6O_{40}$. *Mater. Res. Bull.* **1988**, *23*, 647-651.
(32) Slade, R. C. T.; Ramanan, A.; Hirst, P. R.; Pressman, H. A. Inelastic Neutron Scattering Spectra of Hydrogen and Ammonium Insertion Compounds of Metal Oxides MO_3 (M= Mo and/or W). *Mater. Res. Bull.* **1988**, *23*, 793-798.
(33) Kandemir, T.; Friedrich, M.; Parker, S. F.; Studt, F.; Lennon, D.; Schlögl, R.; Behrens, M. Different Routes to Methanol: Inelastic Neutron Scattering Spectroscopy of Adsorbates on Supported Copper Catalysts. *Phys. Chem. Chem. Phys.* **2016**, *18*, 17253-17258.
(34) Sivadinarayana, C.; Choudhary, T. V.; Daemen, L. L.; Eckert, J.; Goodman, D. W. The Nature of the Surface Species Formed on Au/TiO_2 during the Reaction of H_2 and O_2: An Inelastic Neutron Scattering Study. *J. Am. Chem. Soc.* **2004**, *126*, 38-39.
(35) Mitchell, P. C.; Green, D. A.; Payen, E.; Evans, A. C. Hydrogen in Molybdenum and Cobalt Sulfide Catalysts. A Neutron Compton Scattering Study on the ISIS Electronvolt Spectrometer. *J. Chem. Soc., Faraday Trans.* **1995**, *91*, 4467-4469.
(36) Jobic, H.; Clugnet, G.; Lacroix, M.; Yuan, S. B.; Mirodatos, C.; Breysse, M. Identification of New Hydrogen Species Adsorbed on Ruthenium Sulfide by Neutron Spectroscopy. *J. Am. Chem. Soc.* **1993**, *115*, 3654-3657.
(37) Eβmann, R.; Jacobs, H.; Tomkinson, J. Neutron Vibrational Spectroscopy of Imide Ions (NH_2−) in Bariumimide (BaNH). *J. Alloys Compd.* **1993**, *191*, 131-134.
(38) Tomkinson, J.; Dasannacharya, B. A.; Goyal, P. S.; Chakravarthy, R. Localised Dynamics of a Dipolar Glass $(NH_4)_xK_{1-x}I$. *J. Chem. Soc., Faraday Trans.* **1991**, *87*, 3431-3433.
(39) Tijm, P. J. A.; Waller, F. J.; Brown, D. M. Methanol Technology Developments for the New Millennium. *Appl. Catal., A* **2001**, *221*, 275-282.
(40) Moon, J.; Cheng, Y.; Daemen, L. L.; Li, M. J.; Polo-Garzon, F.; Ramirez-Cuesta, A. J.; Wu, Z. L. Discriminating the Role of Surface Hydride and Hydroxyl for Acetylene Semihydrogenation over Ceria through in situ Neutron and Infrared Spectroscopy. *ACS Catal.* **2020**, *10*, 5278-5287.
(41) McFarlane, A. R.; Silverwood, I. P.; Warringham, R.; Norris, E. L.; Ormerod, R. M.; Frost, C. D.; Parker, S. F.; Lennon, D. The Application of Inelastic Neutron Scattering to Investigate the 'Dry' Reforming of Methane over an Alumina-supported Nickel Catalyst Operating under Conditions Where Filamentous Carbon Formation is Prevalent. *RSC Adv.* **2013**, *3*, 16577-16589.
(42) Schenkel, R.; Jentys, A.; Parker, S. F.; Lercher, J. A. Investigation of the Adsorption of Methanol on Alkali Metal Cation Exchanged Zeolite X by Inelastic Neutron Scattering. *J. Phys. Chem. B* **2004**, *108*, 7902-7910.
(43) McInroy, A. R.; Lundie, D. T.; Winfield, J. M.; Dudman, C. C.; Jones, P.; Parker, S. F.; Taylor, J. W.; Lennon, D. An Infrared and Inelastic Neutron Scattering Spectroscopic Investigation on the Interaction of η-Alumina and Methanol. *Phys. Chem. Chem. Phys.* **2005**, *7*, 3093-3101.
(44) Poulston, S.; Holroyd, R. P.; Bowker, M.; Parker, S. F.; Mitchell, P. C. H. An Inelastic Neutron Scattering Study of Formate on Copper Surfaces. *Surf. Sci.* **1998**, *402*, 599-603.
(45) Duong, T. D.; Sapchenko, S. A.; Da Silva, I.; Godfrey, H. G.; Cheng, Y.; Daemen, L. L.; Manuel, P.; Ramirez-Cuesta, A. J.; Yang, S.; Schröder, M. Optimal Binding of Acetylene to a Nitro-decorated Metal–organic Framework. *J. Am. Chem. Soc.* **2018**, *140*, 16006-16009.
(46) Schärringer, P.; Müller, T. E.; Jentys, A.; Lercher, J. A. Identification of Reaction Intermediates during Hydrogenation of CD_3CN on Raney-Co. *J. Catal.* **2009**, *263*, 34-41.
